# New Anti-Inflammatory Metabolites by Microbial Transformation of Medrysone

**DOI:** 10.1371/journal.pone.0153951

**Published:** 2016-04-22

**Authors:** Saira Bano, Atia-tul- Wahab, Sammer Yousuf, Almas Jabeen, Mohammad Ahmed Mesaik, Atta-ur- Rahman, M. Iqbal Choudhary

**Affiliations:** 1 H. E. J. Research Institute of Chemistry, International Center for Chemical and Biological Sciences, University of Karachi, Karachi, 75270, Pakistan; 2 Dr. Panjwani Center for Molecular Medicine and Drug Research, International Center for Chemical and Biological Sciences, University of Karachi, Karachi, 75270, Pakistan; 3 Department of Biochemistry, Faculty of Science, King Abdulaziz University, Jeddah, 21412, Saudi Arabia; 4 Tabuk Medical College, University of Tabuk, Tabuk, 71491, Saudi Arabia; Aligarh Muslim University, INDIA

## Abstract

Microbial transformation of the anti-inflammatory steroid medrysone (**1**) was carried out for the first time with the filamentous fungi *Cunninghamella blakesleeana* (ATCC 8688a), *Neurospora crassa* (ATCC 18419), and *Rhizopus stolonifer* (TSY 0471). The objective was to evaluate the anti-inflammatory potential of the substrate (**1**) and its metabolites. This yielded seven new metabolites, 14α-hydroxy-6α-methylpregn-4-ene-3,11,20-trione (**2**), 6β-hydroxy-6α-methylpregn-4-ene-3,11,20-trione (**3**), 15β-hydroxy-6α-methylpregn-4-ene-3,11,20-trione (**4**), 6β,17α-dihydroxy-6α-methylpregn-4-ene-3,11,20-trione (**5**), 6β,20*S*-dihydroxy-6α-methylpregn-4-ene-3,11-dione (**6**), 11β,16β-dihydroxy-6α-methylpregn-4-ene-3,11-dione (**7**), and 15β,20*R*-dihydroxy-6α-methylpregn-4-ene-3,11-dione (**8**). Single-crystal X-ray diffraction technique unambiguously established the structures of the metabolites **2**, **4**, **6**, and **8**. Fungal transformation of **1** yielded oxidation at the C-6β, -11β, -14α, -15β, -16β positions. Various cellular anti-inflammatory assays, including inhibition of phagocyte oxidative burst, T-cell proliferation, and cytokine were performed. Among all the tested compounds, metabolite **6** (IC_50_ **=** 30.3 μg/mL) moderately inhibited the reactive oxygen species (ROS) produced from zymosan-induced human whole blood cells. Compounds **1**, **4**, **5**, **7**, and **8** strongly inhibited the proliferation of T-cells with IC_50_ values between <0.2–10.4 μg/mL. Compound **7** was found to be the most potent inhibitor (IC_50_ < 0.2 μg/mL), whereas compounds **2**, **3**, and **6** showed moderate levels of inhibition (IC_50_ = 14.6–20.0 μg/mL). Compounds **1**, and **7** also inhibited the production of pro-inflammatory cytokine TNF-α. All these compounds were found to be non-toxic to 3T3 cells (mouse fibroblast), and also showed no activity when tested against HeLa (human epithelial carcinoma), or against PC3 (prostate cancer) cancer cell lines.

## Introduction

Microbial transformation is an effective tool for structural derivatizations that are difficult to achieve by conventional chemical methods. Microbial systems are also extensively employed in the study of drug metabolism and bioremediation [1, 2].

Steroids are among the most widely marketed pharmaceutical products. Several steroids are used as anabolic, contraceptive, anti-androgenic, anti-inflammatory, and anti-cancer agents. Microbial hydroxylation of steroids is an efficient method for the synthesis of new hydroxysteroids with high stereo- and regio-selectivity, and for study of the steroidal metabolism [1, 3–5]. The bioconversion of steroids was initiated in 1950. In 1952, progesterone was converted into 11α-hydroxyprogesterone, which was later used as an intermediate in the synthesis of cortisone [6].

During the last two decades, we have been focusing on the structural modifications of various classes of steroids in search of their new bioactive analogues [7–22]. The aim of the current study was to synthesize new anti-inflammatory metabolites of a synthetic corticosteroid, medrysone (**1**) by biotransformation. Medrysone (11β-hydroxy-6α-methylpregn-4-ene-3,20-dione) is an anti-inflammatory agent used in ophthalmic treatments. This is the first report of the microbial transformation of medrysone (**1**) with the filamentous fungi, *C*. *blakesleeana* (ATCC 8688a), *N*. *crassa* (ATCC 18419), and *R*. *stolonifer* (TSY 0471). Fermentation of medrysone with these fungi yielded seven new metabolites **2–8**. Various cellular assays, such as phagocyte oxidative burst, T-cell proliferation, and cytokines analysis, were performed on the substrate **1**, and metabolites **2–8** to evaluate their anti-inflammatory potential.

## Experimental

### General

Medrysone (**1**) was obtained from Sigma-Aldrich. Precoated TLC plates (silica gel, 20×20, 0.25 mm thick PF_254_, Merck, Germany) were used for thin layer chromatography, ceric sulfate solution was used as staining reagent. Column chromatography was performed on silica gel (70–230 mesh, Merck). Recycling preparative HPLC separation was performed on a JAI LC-908W instrument, equipped with YMC L-80 (4–5 μm, 20−50 mm i.d.) using MeOH-H_2_O as the mobile phase, with UV detection at 254 nm. Electron impact mass spectra (EI-MS) and high resolution electron impact mass spectra (HREI-MS) were recorded on JEOL JMS600H mass spectrometer (JEOL, Akishima, Japan). Electrospray ionization mass spectra (ESI-MS) and high resolution electrospray ionization mass spectra (HRESI-MS) were measured on QSTAR XL mass spectrometer (Applied Biosystem/ MDS Sciex, Darmstadt, Germany). ^1^H- and ^13^C-NMR spectra were recorded on a Bruker Avance 300 and 600 MHz NMR spectrometers (Bruker, Zurich, Switzerland) in CDCl_3_, CD_3_OD and DMSO-*d*_*6*_. Melting points (m.p.) were determined on Buchi-560 (Büchi, Flawil, Switzerland) apparatus. Hitachi U-3200 (Hitachi, Tokyo, Japan) spectrophotometer was used to collect UV spectra (nm). FT-IR-8900 (Shimadzu, Japan) and Bruker VECTOR 22 (Bruker, France) spectrophotometers were employed to obtain infrared (IR) spectra (cm^-1^). Digital polarimeter JASCO P-2000 (JASCO, Japan) was used to measure optical rotations in chloroform or methanol. Bruker SMART APEX II single-crystal X-ray diffractometer, fitted with CCD detector, was used to collect X-ray data [23]. SAINT program was used to analyze data, solved with the aid of direct methods [24], and refined with the help of SHELXTL-PC package [25]. The figures were plotted by ORTEP program [26].

### Microorganisms and culture conditions

Fungal cultures for biotransformation were purchased from the American Type Culture Collection (ATCC, Virginia, USA), and National Institute of Health Sciences (TSY, Tokyo, Japan). Stock cultures of fungi were stored on Sabouraud dextrose agar (SDA) at 4°C.

Glucose (60.0 g), glycerol (60.0 mL), peptone (30.0 g), yeast extract (30.0 g), KH_2_PO_4_ (30.0 g), and NaCl (30.0 g) were added to distilled H_2_O (6.0 L) to prepare the 6.0 L of media for *C*. *blakesleeana* (ATCC 8688a).

The following ingredients were used for the media preparation of *R*. *stolonifer* (TSY 0471): glucose (80.0 g), peptone (20.0 g), KH_2_PO_4_ (20.0 g), and yeast extract (12.0 g), pH 5.6 in distilled water (4.0 L).

The culture medium (6.0 L) for *N*. *crassa* (ATCC 18419) was prepared by adding the following ingredients: glucose (90.0 g), sucrose (90.0 g), peptone (30.0 g), KH_2_PO_4_ (6.0 g), KCl (5.0 g), MgSO_4_ (3.0 g), and FeSO_4_ (0.06 g mL) into distilled water (6 L).

### General fermentation and extraction protocol

Biotransformation studies were carried out by using stage II fermentation protocol [27]. Stage I cultured flasks were prepared by transferring the spores from 3 day old slants, which were then incubated for 4 days on a rotary shaker (128 rpm) at 25–28°C. Aliquots (5 mL) from the stage I cultured flask were then transferred to the remaining flasks, and incubated on a rotary shaker (128 rpm) at 25–28°C. After 2 days, compound **1** was dissolved in acetone, and evenly distributed among all the flasks. Fermentation was continued and time course studies were performed after different time intervals to assess the degree of transformation. After completion of 12–14 days, the broth was filtered to separate mycelia and washed with dichloromethane. The filtrate was then extracted with the same solvent *i*.*e*. dichloromethane. The solvent was dried with anhydrous sodium sulfate (Na_2_SO_4_), and evaporated under reduced pressure to obtain the crude extract. Two parallel control experiments were also performed as positive (media with compound only), and negative (media with fungus only) controls.

#### Fermentation of medrysone (1) with *C*. *blakesleeana* (ATCC 8688a)

**Fig 1:**
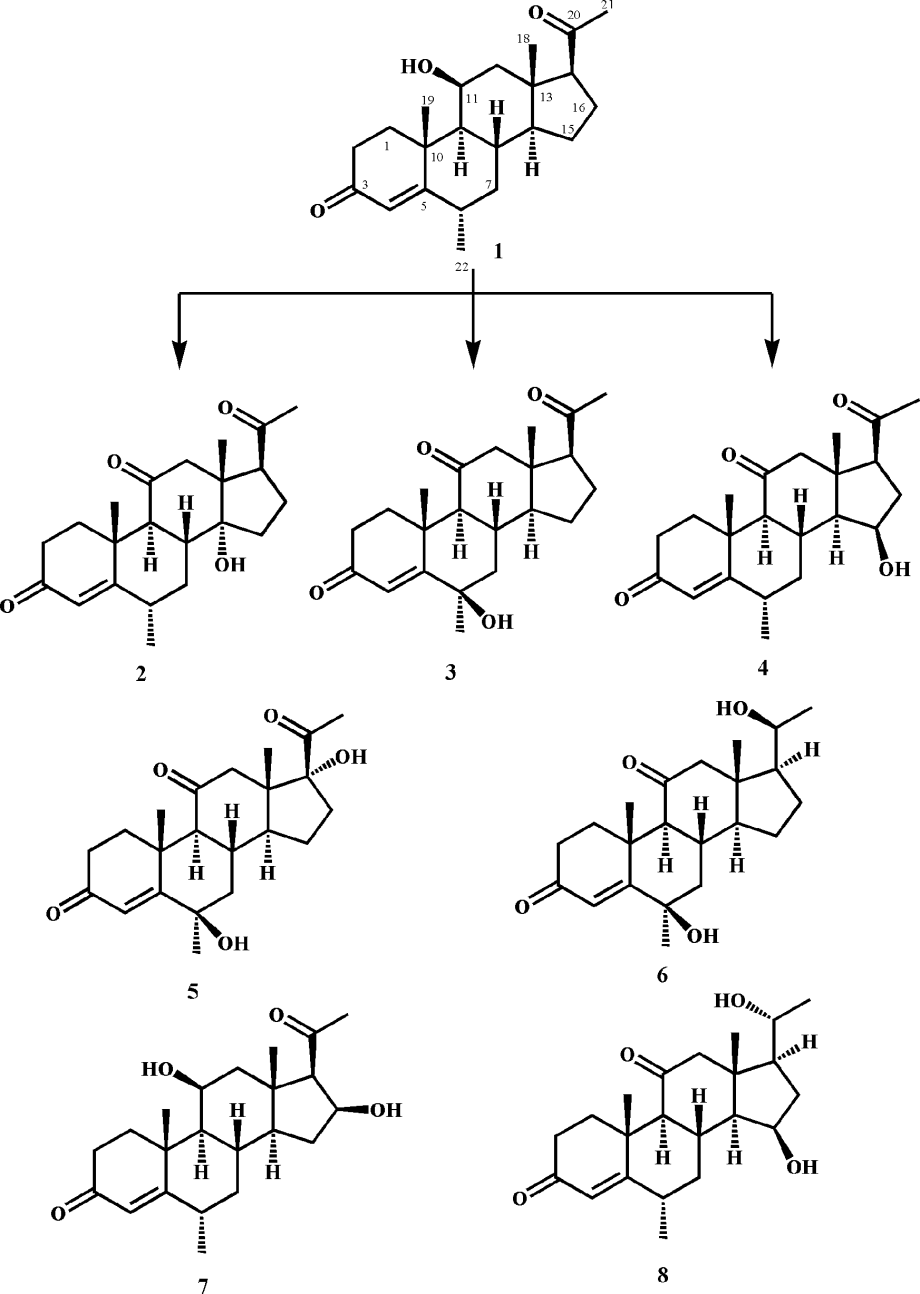
Biotransformation of medrysone (1) with *Cunninghamella blakesleeana*.

Compound **1** (900 mg/30 mL acetone) was distributed in a total of 60 flasks containing the culture of *C*. *blakesleeana* (ATCC 8688a) and left on a rotary shaker for 8 days at 27°C. The medium was separated from the mycelium by filtration. The filtered medium was then extracted with dichloromethane (6 × 3 L), dried over anhydrous sodium sulfate (Na_2_SO_4_), and evaporated on a rotary evaporator to afford a brown crude extract (0.90 g). The extract was subjected to gradient elution with acetone and petroleum ether to obtain four main fractions (1–4). These fractions were purified by using recycling reverse phase HPLC to obtain compounds **2–8** (Fig 1). Fraction 1 was subjected to recycling RP-HPLC (L80, MeOH: H_2_O = 2:1, 4 mL/min) to afford pure compounds **2** (17 mg, R_t_: 38 min), and **3** (14 mg, R_t_: 38 min). Compounds **4** (10 mg, R_t_: 38 min), and **5** (15 mg, R_t_: 36 min) were obtained from fraction 2 by using recycling RP-HPLC (L80, MeOH: H_2_O = 2:1, 4 mL/min). Similarly, fraction 3 yielded compounds **6** (5.8 mg, R_t_: 36 min), and **7** (6 mg, R_t_: 42 min) by using recycling RP-HPLC (L80, MeOH: H_2_O = 2:1, 4 mL/min). Compound **8** (11 mg, R_t_: 70 min) was obtained from fraction 4 by using recycling RP-HPLC (L80, MeOH: H_2_O = 1:1, 4 mL/min).

#### 14α-Hydroxy-6α-methylpregn-4-ene-3,11,20-trione (2)

Colorless crystalline solid: m.p.: 209–210°C; ${\left[\alpha \right]}_{\text{D}}^{25}$: +251 (*c* = 0.1, CHCl_3_); UV (MeOH) λ_max_ nm (log ⊰): 248 (6.0); IR (KBr) ν_max_ cm^-1^: 3466 (OH), 1700 (C = O), 1662 (C = C = O); ^1^H-NMR (CDCl_3_, 300 MHz): Table 1; ^13^C-NMR (CDCl_3_, 150 MHz): Table 2; EI-MS *m/z* (rel. int., %): 358 [M^+^] (84.3), 340 (69), 269 (37), 177 (100), 161 (63), 136 (73), 43 (62); HREI-MS *m/z* (mol. formula, calcd. value): 358.2128 (C_22_H_30_O_4_, 358.2139); Single-crystal X-ray diffraction data: Empirical formula = C_22_H_30_O_4_, M_r_ = 358.46, Crystal system: Orthorhombic, space group: P2_1_2_1_2_1_, Unit cell dimensions: **a** = 6.1067(8) Å, **b** = 13.813(2) Å, **c** = 23.065(3) Å, Volume: 1945.6(5) Å^3^, Z = 4, *ρ*_calc_ = 1.224 Mg/m^3^, F(000) 776, Crystal size: 0.32 × 0.10 × 0.10 mm, θ range for data collection: 1.72 to 25.50°, Total 11,616 reflections were collected, out of which 3,620 reflections were judged observed (R_int_ = 0.0509). Final R indices were, R_1_ = 0.0587 for [I>2σ (I)], wR_2_ = 0.1333, R indices were R_1_ = 0.0982, wR_2_ = 0.1581 for all data, largest difference peak and hole: 0.471 and -0.185 e. Å^-3^. Crystallographic data for compound **2** was deposited in the Cambridge Crystallographic Data Center (CCDC 1053672).

**Table 1:** ^1^H-NMR chemical shift data of compounds 1–8 (δ in ppm, *J* in Hz).

Carbon	1^a^	2^b^	3^b^	4^b^	5^b^	6^c^	7^a^	8^a^
1	2.16, m; 1.93, m	2.78, m; 1.77, m	2.34, m; 1.58, m	2.73, m; 1.65, m;	2.78, m; 1.65, m	2.82, m; 1.58, m	2.18, m; 1.93, m	2.65, m; 1.72, m
2	2.45, m; 2.33, m	2.44, m; 2.30, m	2.55, m; 2.33, m	2.48, m; 2.28, m	2.59, m; 2.28, m	2.57, m; 2.36, m	2.45, m; 2.33, m	2.54, m; 2.23, m
3	-	-	-	-	-	-	-	-
4	5.69, s	5.77, s	6.00, s	5.80, s	5.98, s	6.00, s	5.60, s	5.74, s
5	-	-	-	-	-	-	-	-
6	-	2.44, m	-	2.43, m	-	-	2.61, m	2.55, m
7	2.04, m; 0.82, m	1.76, m; 1.32, m	2.02, dd (*J*_7β, 7α_ = 13.8, *J*_7β, 8β_ = 3.0); 1.35, m	2.23, m; 1.05, m	1.98, m; 1.45, m	2.01, m; 1.33, m	2.02, m; 0.83, m	2.30, m; 1.08, m
8	2.08, m	2.25, m	2.40, m	2.40, m	1.59, m	2.33, m	2.09, m	2.42, m
9	1.07, m	2.52, m	2.70, m	1.97, m	2.07, m	1.85, m	1.10, dd (*J*_9α, 8β_ = 11.3, *J*_9α, 11α_ = 3.2)	2.19, m
10	-	-	-	-	-	-	-	-
11	3.50, br. s	-	-	-	-	-	4.34, br. d (*J*_11α, 12α_ = 2.9)	-
12	2.23, dd (*J*_12β, 12α_ = 13.8, *J*_12β, 11α_ = 2.7); 1.64, m	3.15, d (*J*_12α,12β_ = 12.3); 2.35, d (*J*_12β,12α_ = 12.3)	2.74, d (*J*_12α, 12β_ = 12.3); 2.45, d (*J*_12β, 12α_ = 12.3)	2.58, overlapped d (*J*_12β,12α_ = 12.0): 2.48, d (*J*_12α,12β_ = 12.0)	2.97, d (*J*_12α, 12β_ = 12.6); 2.12, d (*J*_12β, 12α_ = 12.6)	2.49, d (*J*_12α, 12β_ = 12.5): 2.22, d (*J*_12β, 12α_ = 12.5)	2.15, m; 1.73, dd (*J*_12α 12β_ = 13.5, *J*_12α, 11α_ = 2.9)	2.37, d (*J*_12α_, _12β_ = 12.3); 2.32, d (*J*_12β_, _12α_ = 12.3)
13	-	-	-	-	-	-	-	-
14	2.39, m	-	1.70, m	1.60, m	2.44, m	1.66, m	1.54, m	1.62, m
15	1.75, m; 1.33, m	1.83, m; 1.63, m	2.25, m; 1.44, m	4.40, m	1.92, m; 1.40, m	2.04, m; 1.68, m	1.83, m; 1.62, m	4.32, t (*J*_15α, 14α/ 16α_ = 5.7)
16	2.14, m; 1.63, m	1.93, m	1.84, m	2.32, m; 2.36, m	2.59, m; 2.27, m	1.84, m; 1.33, m	4.72, t (*J*_16α,15αβ/17α_ = 7.3 Hz)	2.57, m; 1.70, m
17	-	3.38, t (*J*_17α. 16 α, β_ = 8.4)	2.70, m	2.66, m	-	1.57, m	2.49, m	1.52, m
18	0.86, s	0.72, s	0.65, s	0.87, s	0.56, s	0.60, s	0.88, s	0.90, s
19	1.44, s	1.39, s	1.59, s	1.43, s	1.49, s	1.60, s	1.45, s	1.45, s
20	-	-	-	-	-	3.70, m	-	3.66, m
21	2.11, s	2.09, s	2.09, s	2.11, s	2.17, s	1.20, d (*J*_21,20β_ = 6.1)	2.17, s	1.11, d (*J*_21, 20β_ = 6.3)
22	1.06, d (*J*_22, 6β_ = 6.3)	1.10, d (*J*_22, 6β_ = 6.3)	1.42, s	1.09, d (*J*_22, 6β_ = 6.6)	1.29, s	1.42, s	1.05, d (*J*_22,6a_ = 6.4)	1.17, d (*J*_21, 6β_ = 6.0)

^**a**^ 300 MHz, CD_3_OD

^**b**^ 300 MHz, CDCl_3_

^**c**^ 600 MHz, CDCl_3_

**Table 2:** ^13^C-NMR chemical shift data of compounds 1–8.

Carbon	1^a^	2^b^	3^c^	4^d^	5^e^	6^b^	7^e^	8^e^
1	35.8	35.0	36.4	34.9	37.3	36.3	35.7	35.7
2	34.3	33.2	33.7	33.5	34.4	33.6	34.3	34.2
3	202.7	199.9	200.7	199.9	203.5	200.9	202.7	202.7
4	119.8	122.0	123.6	122.1	124.0	123.5	119.9	122.1
5	179.6	171.0	168.5	171.6	172.1	168.8	179.4	176.2
6	34.5	33.3	71.0	33.2	71.2	71.1	34.5	34.7
7	43.4	36.1	45.7	40.1	47.1	45.7	43.3	41.4
8	32.6	40.0	31.9	32.7	33.0	31.7	32.2	33.8
9	57.4	56.9	62.5^f^	63.1	63.2	62.3	57.3	63.6
10	40.9	38.0	38.4	38.6	39.6	38.3	40.9	39.9
11	68.6	209.0	207.9	207.6	212.3^f^	209.3	68.4	211.9
12	48.6	50.5	56.7	57.7	51.8	57.1	48.6	58.8
13	44.5	51.0	46.9	46.6	47.2	45.7	46.9	46.4
14	58.6	83.5	54.7	59.3	50.4	54.5	56.4	60.0
15	25.3	32.4	23.9	69.2	24.1	25.8	36.4	69.4
16	23.6	22.1	23.4	36.7	34.2	23.6	72.5	40.8
17	64.9	58.0	62.1^f^	62.0	90.0	57.0	74.9	58.2
18	16.1	18.9	14.3	16.7	16.2	13.6	17.5	16.1
19	22.6	18.2	19.6	18.0	19.9	19.6	22.6	18.6
20	212.5	208.5	207.7	206.6	212.3^f^	69.4	210.5	70.2
21	31.4	31.0	31.2	31.2	27.3	23.4	31.9	23.8
22	18.5	18.8	29.2	18.3	28.9	29.2	18.5	18.5

^**a**^ 125 MHz, CD_3_OD

^**b**^ 150 MHz, CDCl_3_

^**c**^ 100 MHz, CDCl_3_

^**d**^ 125 MHz, CDCl_3_

^**e**^ 150 MHz, CD_3_OD

#### 6β-Hydroxy-6α-methylpregn-4-ene-3,11,20-trione (3)

Colorless amorphous solid; ${\left[\alpha \right]}_{\text{D}}^{25}$: +32 (*c* = 0.1, CHCl_3_); UV (MeOH) λ_max_ nm (log ⊰): 248 (6.0); IR (CHCl_3_) ν_max_ cm^-1^: 3479 (OH), 1707 (C = O), 1678 (C = C = O); ^1^H-NMR (CDCl_3_, 300 MHz): Table 1; ^13^C-NMR (CDCl_3,_ 100 MHz): Table 2; EI-MS *m/z* (rel. int., %): 358 [*M*^+^] (4), 315 (100), 175 (5), 149 (7), 123 (6), 85 (8), 43 (39); HREI-MS *m/z* (mol. formula, calcd value): 358.2115 (C_22_H_30_O_4_, 358.2139).

#### 15β-Hydroxy-6α-methylpregn-4-ene-3,11,20-trione (4)

Colorless crystalline solid; m.p.: 185–186°C; ${\left[\alpha \right]}_{\text{D}}^{25}$: +28 (*c* = 0.14, CHCl_3_); UV (MeOH) λ_max_ nm (log ⊰): 248 (5.9); IR (CHCl_3_) ν_max_ cm^-1^: 3423 (OH), 1703 (C = O), 1658 (C = C = O); ^1^H-NMR (CDCl_3_, 300 MHz): Table 1; ^13^C-NMR (CDCl_3_, 125 MHz): Table 2; EI-MS *m/z* (rel. int., %) 358 [*M*^+^] (63), 343 (44), 259 (24), 161 (30), 136 (100), 121 (25), 43 (62); HREI-MS *m/z* (mol. formula, calcd. value): 358.2126 (C_22_H_30_O_4_, 358.2139); Single-crystal X-ray diffraction data: Empirical formula = C_22_H_30_O_4_, *Mr* = 358.46, Crystal system: Triclinic, space group: P_1_, Unit cell dimensions: **a** = 6.5208(6) Å, **α** = 97.416(2)°, **b** = 7.4210(6) Å, **β** = 101.439(2)°, **c** = 10.9061(9) Å, **γ** = 107.064(2)°, Volume: 484.52(7) Å^3^, Z = 1, *ρ*_calc_ = 1.229 mg/m^3^, F(000): 194, Crystal size: 0.55 × 0.38 × 0.30 mm, *θ* range for data collection: 1.94 to 28.29°. Total 6,591 reflections were collected, out of which 4,741 reflections were judged observed (*R*_int_ = 0.0118). Final R indices were, R_1_ = 0.0461 for [I>2s*σ* (I)], wR_2_ = 0.1169, R indices were R_1_ = 0.0503, wR_2_ = 0.1213 for all data, largest difference peak and hole: 0.295 and -0.187 e. Å^-3^. Crystallographic data for compound **4** was deposited in the Cambridge Crystallographic Data Center (CCDC 1402957).

#### 6β,17α-Dihydroxy-6α-methylpregn-4-ene-3,11,20-trione (5)

Colorless crystalline solid; m.p.: 289–291°C; ${\left[\alpha \right]}_{\text{D}}^{25}$: -81 (*c* = 0.05, CHCl_3_); UV (MeOH) λ_max_ nm (log ⊰): 248 (6.3); IR (CHCl_3_) ν_max_ cm^-1^: 3450 (OH), 1699 (C = C = O); ^1^H-NMR (CDCl_3_, 300 MHz): Table 1; ^13^C-NMR (CD_3_OD, 150 MHz): Table 2; EI-MS *m/z* (rel. int., %): 374 [*M*^+^] (58), 356 (68), 303 (54), 270 (62), 255 (63), 220 (45), 175 (42), 43 (100); HREI-MS *m/z* (mol. formula, calcd. value): 374.2068 (C_22_H_30_O_5_, 374.2088).

#### 6β,20*S*-Dihydroxy-6α-methylpregn-4-ene-3,11-dione (6)

Colorless crystalline solid; m.p.: 229–230°C; ${\left[\alpha \right]}_{\text{D}}^{25}$: +126 (*c* = 0.1, CHCl_3_); UV (MeOH) λ_max_ nm (log ⊰): 247 (5.7); IR (CHCl_3_) ν_max_ cm^-1^: 3445 (OH), 1742 (C = O), 1662 (C = C = O); ^1^H-NMR (CDCl_3_, 600 MHz): Table 1; ^13^C-NMR (CDCl_3_, 150 MHz): Table 2; HRESI-MS [M+H]^+^ *m/z*: 361.2382 (C_22_H_32_O_4_+H requires 361.2378); Single-crystal X-ray diffraction data: Empirical formula = C_22_H_32_O_4_, *Mr* = 360.48, Crystal system: Orthorhombic, space group: P2_1_2_1_2_1_, Unit cell dimensions: **a** = 6.0392(8) Å, **α** = 90°, **b** = 13.5095(16) Å, **β** = 90°, **c** = 23.795(3) Å, **γ** = 90°, Volume 1941.3(4) Å ^3^, Z = 4, *ρ*_calc_ = 1.233 mg/m^3^, F(000): 784, Crystal size: 0.50 × 0.24 × 0.13 mm, *θ* range for data collection: 1.71 to 25.49°. Total 11,559 reflections were collected, out of which 2,108 reflections were judged observed (*R*_int_ = 0.1006). Final R indices were R_1_ = 0.0548 for for [I>2si*σ* (I)], wR_2_ = 0.0933, R indices were R_1_ = 0.1153, wR_2_ = 0.1163 for all data largest difference peak and hole: 0.142 and -0.123 e. Å^-3^. Crystallographic data for compound **6** was deposited in the Cambridge Crystallographic Data Center (CCDC 1053673).

#### 11β,16β -Dihydroxy-6α-methylpregn-4-ene-3,11-dione (7)

Colorless crystalline solid; ${\left[\alpha \right]}_{\text{D}}^{25}$: +375 (*c* = 0.1, CHCl_3_); UV (MeOH) λ_max_ nm (log ⊰): 247 (6.0); IR (CHCl_3_) ν_max_ cm^-1^: 3402 (OH), 1703 (C = O), 1662 (C = C = O); ^1^H-NMR (CD_3_OD, 300 MHz): Table 1; ^13^C-NMR (CD_3_OD, 150 MHz): Table 2; HRESI-MS [M+H]^+^ *m/z*: 361.2374 (C_22_H_32_O_4_+H requires 360.2378).

#### 15β,20*R*-Dihydroxy-6α-methylpregn-4-ene-3,11-dione (8)

Colorless solid; m.p.: 274–275°C; ${\left[\alpha \right]}_{\text{D}}^{25}$: +117 (*c* = 0.1, CHCl_3_); UV (MeOH) λ_max_ nm (log ⊰): 247 (6.0); IR (CHCl_3_) ν_max_ cm^-1^: 3431 (OH), 1703 (C = O), 1662 (C = C = O); ^1^H-NMR (CD_3_OD, 300 MHz): Table 1; ^13^C-NMR (CD_3_OD, 150 MHz): Table 2; EI-MS *m/z* (rel. int., %): 360 [M^+^] (17), 342 (9), 225 (9), 177 (16), 136 (55), 105 (33), 91 (61), 55 (100); HREI-MS *m/z* (mol. formula, calcd. value): 360.2295 (C_22_H_32_O_4_, 360.2295); Single-crystal X-ray diffraction data: Empirical formula = C_22_ H_34_ O_5_, *Mr* = 378.49, Crystal system: Monoclinic, space group: P2_1_, Unit cell dimensions: **a** = 9.3482(14) Å, **α** = 90°, **b** = 9.4131(14) Å, **β** = 102.433(3°, **c** = 12.0508(17) Å, **γ** = 90°, Volume 1035.5(3) Å^3^, Z = 2, *ρ*_calc_ = 1.214 mg/m^3^, F(000): 412, Crystal size: 0.45 × 0.38 × 0.16 mm, *θ* range for data collection: 1.73 to 25.49°. Total 6,011 reflections were collected, out of which 3,508 were judged observed (*R*_int_ = 0.0152). Final R indices were R_1_ = 0.0378 for [I>2s*σ* (I)], wR_2_ = 0.0899, R indices were R_1_ = 0.0434, wR_2_ = 0.0936 for all data, largest difference peak and hole: 0.205 and -0.144 e. Å^-3^. Crystallographic data for compound **8** was deposited in the Cambridge Crystallographic Data Center (CCDC 1053674).

#### Fermentation of medrysone (1) with *R*. *stolonifer* (TSY 0471)

**Fig 2:**
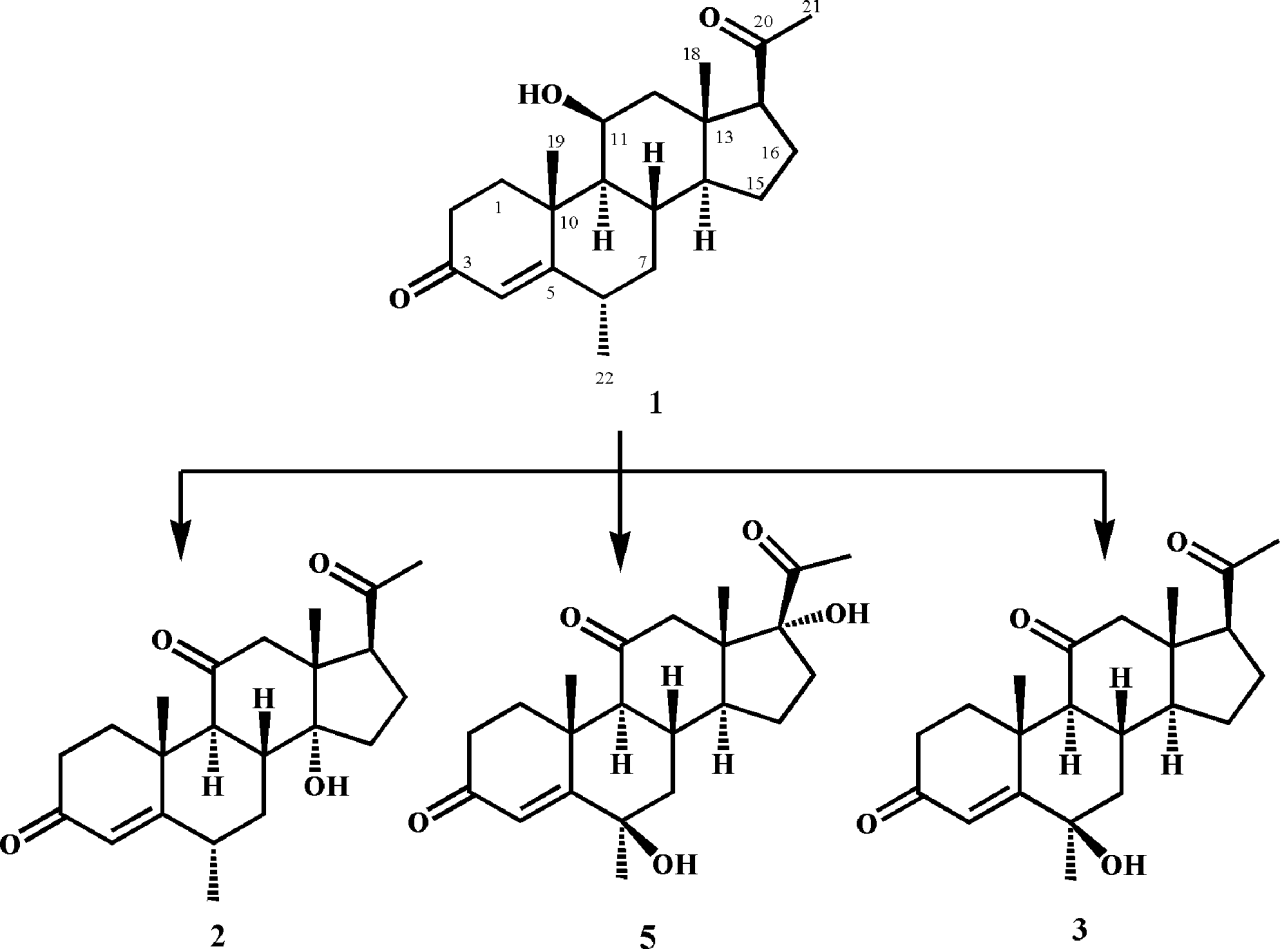
Biotransformation of medrysone (1) with *Rhizopus stolonifer*.

Compound **1 (**600 mg/ 20 mL of acetone) was evenly transferred among 40 flasks containing 4 days old culture of *R*. *stolonifer* TSY (0471). After 14 days of fermentation, the biomass was separated from the medium by filtration and then washed with dichloromethane. The medium was then extracted with the same solvent *i*.*e*. dichloromethane, dried with anhydrous sodium sulfate (Na_2_SO_4_), and evaporated to obtain a brown gum (1.4 g). The gum was then subjected to silica gel column chromatography with the mobile phase consisting of petroleum ether and acetone. Three main fractions (1–3) were obtained and purified to acquire compounds **2**, **3**, and **5** by recycling RPHPLC (L80, MeOH: H_2_O = 2:1, 4 mL/min) (Fig 2).

#### Fermentation of medrysone (1) with *N*. *crassa* (ATCC 18419)

**Fig 3:**
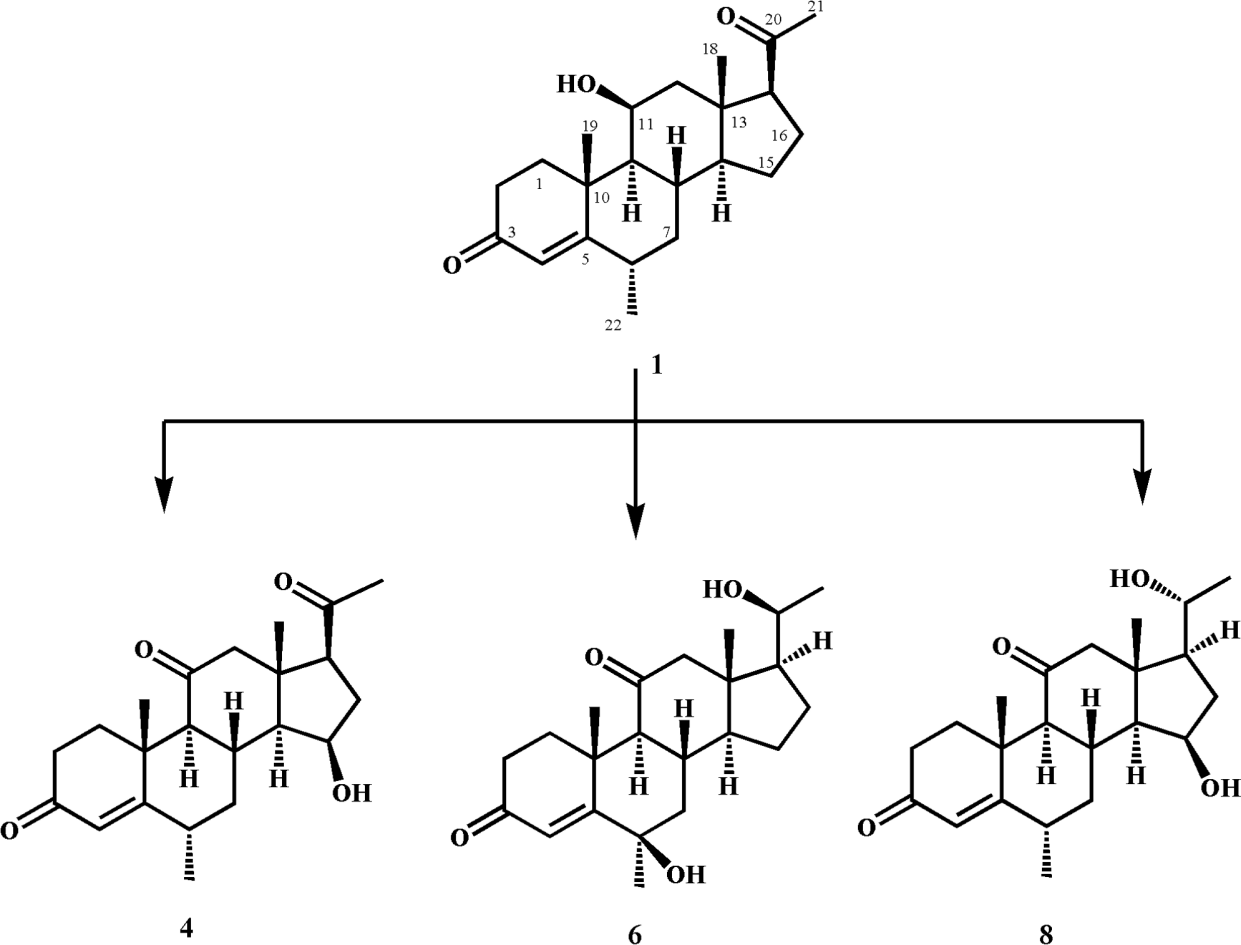
Biotransformation of medrysone (1) with *Neurospora crassa*.

Compound **1** (900 mg/ 60 mL of acetone) was distributed among 60 flasks, containing the fungal culture of *N*. *crassa* (ATCC 18419). These flasks were then kept on a shaker at 27°C for 15 days. After fermentation, the medium was filtered and extracted with dichloromethane. The extract was dried over anhydrous sodium sulfate (Na_2_SO_4_), and evaporated under reduced pressure to obtain brown a gummy material. The crude mass was then subjected to column chromatography using petroleum ether and acetone as the mobile phase. Three main fractions were thus purified to yield compounds **4**, **6**, and **8** by recycling RPHPLC (L80, MeOH: H_2_O = 2:1, 4 mL/min) (Fig 3).

### Biological activity

#### Oxidative burst inhibition assay

Oxidative burst assay was carried out by chemiluminescence technique using luminol as a probe to determine the effect of test compounds on the production of reactive oxygen species (ROS) from phagocytes, employing Helfand *et al*. method with slight modifications [28]. Briefly, 25 μL of whole blood was diluted in Hanks balanced salt solution (HBSS++) supplemented with calcium and magnesium and incubated with three different concentrations (1, 10, and 100 μg/mL) of the test compounds for 15 min at 37°C, followed by addition of 25 μL (20 mg mL^-1^) of zymosan (Sigma Chemical Co., St. Louis, MO, USA) and 25 μL (7 × 10^5^ M) of luminol (Sigma Chemical Co., St. Louis, MO, USA). HBSS^++^ alone was run as a negative control. The level of ROS was measured as RLU (relative light units) with a luminometer (Labsystem Luminoskan RS, Finland). The luminometer was used in repeated scan mode with 50 seconds interval, and one second point measuring time.

#### T-Cell proliferation inhibition assay

The T-Cell proliferation inhibitory activity of compounds was evaluated by following the reported method of Nielsen *et al*. (1998) with slight modifications [29]. Peripheral blood mononuclear cells (PBMC) were isolated from healthy human donors with their consent by Ficoll-Hypaque density gradient centrifugation. The study was carried out after approval from independent ethics committee, ICCBS-UoK, No: ICCBS/IEC-008-BC-2015/Protocol/1.0.

The cells were adjusted to a final concentration of 2 × 10^6^ cells/mL in 5% FBS (fetal bovine serum) containing RPMI 1640 media and added with 5 μg/mL phytohemagglutinin (PHA) in 96-well round bottom plates. Each compound was added in triplicate using four concentrations (0.2, 1, 5, and 25 μg/mL). Positive control wells contained cells and PHA, whereas cells alone served as a negative control. The plate was incubated in 5% CO_2_ at 37°C for 72 h, and cells were pulsed with 25 μL of tritiated thymidine (0.5 μci/well). The incubation was continued for further 18 h. The cells were finally harvested on a glass fiber filter, and the plate was read as count per minute (CPM) using β-scintillation counter.

#### Cytokine inhibition assay

THP-1 (Human monocytic leukemia cells) was purchased from ECACC (85011428, European Collection of Cell Cultures, UK). The cells were maintained in endotoxin free RPMI-1640 containing 5.5 mmol/L glucose (BioM Laboratories, Chemical Division, Malaysia), 50 μmol/L mercaptoethanol Merck (Damstadt, Germany), 10% FBS (fetal bovine serum), 2 mmol/L L-glutamine (PAA Laboratories, GmbH, Pasching, Austria), 1 mmol/L sodium pyruvate (GIBCO, Grand Island, N.Y., U.S.A.), and 10 mmol/L HEPES (MP Biomedicals, lllkirch, France). Upon reaching 70% confluency, 2.5×10^5^ cells/mL cells were plated in 24 well tissue culture plates. The cells were differentiated using 20 ng/mL phorbol myristate acetate (PMA) (SERVA, Heidelberg, Germany) for 24 hours. The cells were stimulated by adding 50 ng/mL of *Escherichia coli* lipopolysaccharide B (DIFCO Laboratories, U.S.A.) and treated with compound (25 μg/mL). The incubation was continued for 4 hours at 37°C in 5% CO_2_. The supernatants collected were analyzed for the level of pro-inflammatory cytokine TNF-α, and quantification was performed by human TNF-α kit (R&D Systems, Minneapolis, U.S.A.), according to manufacturer’s instructions.

#### Cytotoxicity assay

The cytotoxicity of the test compounds against various cell lines was evaluated by MTT (3-[4, 5-dimethylthiazole-2-yl]-2, 5-diphenyl-tetrazolium bromide] colorimetric assay in 96-well flat-bottomed microplates [30]. The prostate cancer PC3 (ATCC CRL-1435), and mouse fibroblast 3T3 (ATCC CRL-1658) cell lines were purchased from the American Type Culture Collection (ATCC, Virginia, USA). The human epithelial adenocarcinoma HeLa cells were kindly provided by Prof. Dr. Anwar Ali Siddiqui from Aga Khan University, Karachi, Pakistan. Dulbecco’s Modified Eagle Medium (DMEM), added with 5% of fetal bovine serum (FBS), 100 IU/mL of penicillin, and 100 μg/mL of streptomycin was used for culturing of the HeLa (human epithelial carcinoma), PC3 (prostate cancer), and 3T3 (mouse fibroblast) cell lines. The cells were grown in 75 cm^3^ flask and incubated at 37°C in 5% CO_2_ incubator. After incubation 100 μL/well cells were introduced in 96-well plate with the concentration of 5×10^4^ cells/mL and incubated again on the same parameters mentioned above. After 24 h incubation, the old media was removed and various concentrations of test compounds (1–30 μM), diluted in 200 μL of fresh media, were added. The plates were further incubated for 48 h, followed by addition of 200 μL MTT (0.5 mg/mL). After 4 h of incubation, 100 μL of DMSO was introduced into each well. The absorbance was measured by microplate reader (Spectra Max plus, Molecular Devices, CA, USA) at 570 nm for the extent of MTT reduction to formazan within cells. The cytotoxicity was recorded as concentrations causing 50% growth inhibition (IC_50_) for HeLa, PC3, and 3T3 cell lines. The results (% inhibition) were processed by using Soft- Max Pro software (Molecular Devices, CA, USA).

$$\%\;\text{Inhibition}=\frac{100-(\text{OD of test compound}-\text{OD of negative control})}{\left(\text{OD of positive control}-\text{OD of negative control}\right)}\times 100$$

## Results and Discussion

Microbial transformation of anti-inflammatory corticosteroid, medrysone [11β-hydroxy-6α-methylpregn-4-ene-3,20-dione (C_22_H_32_O_3_)] (**1**), was carried out for the first time. Fermentation of **1** with *C*. *blakesleeana* (ATCC 8688a), *R*. *stolonifer* (TSY 0471) and *N*. *crassa* (ATCC 18419) yielded seven new metabolites **2–8**. Previously we have reported the biotransformation of several steroids for the synthesis of new anti-inflammatory compounds [31–33].

### Structure elucidation

**Fig 4:**
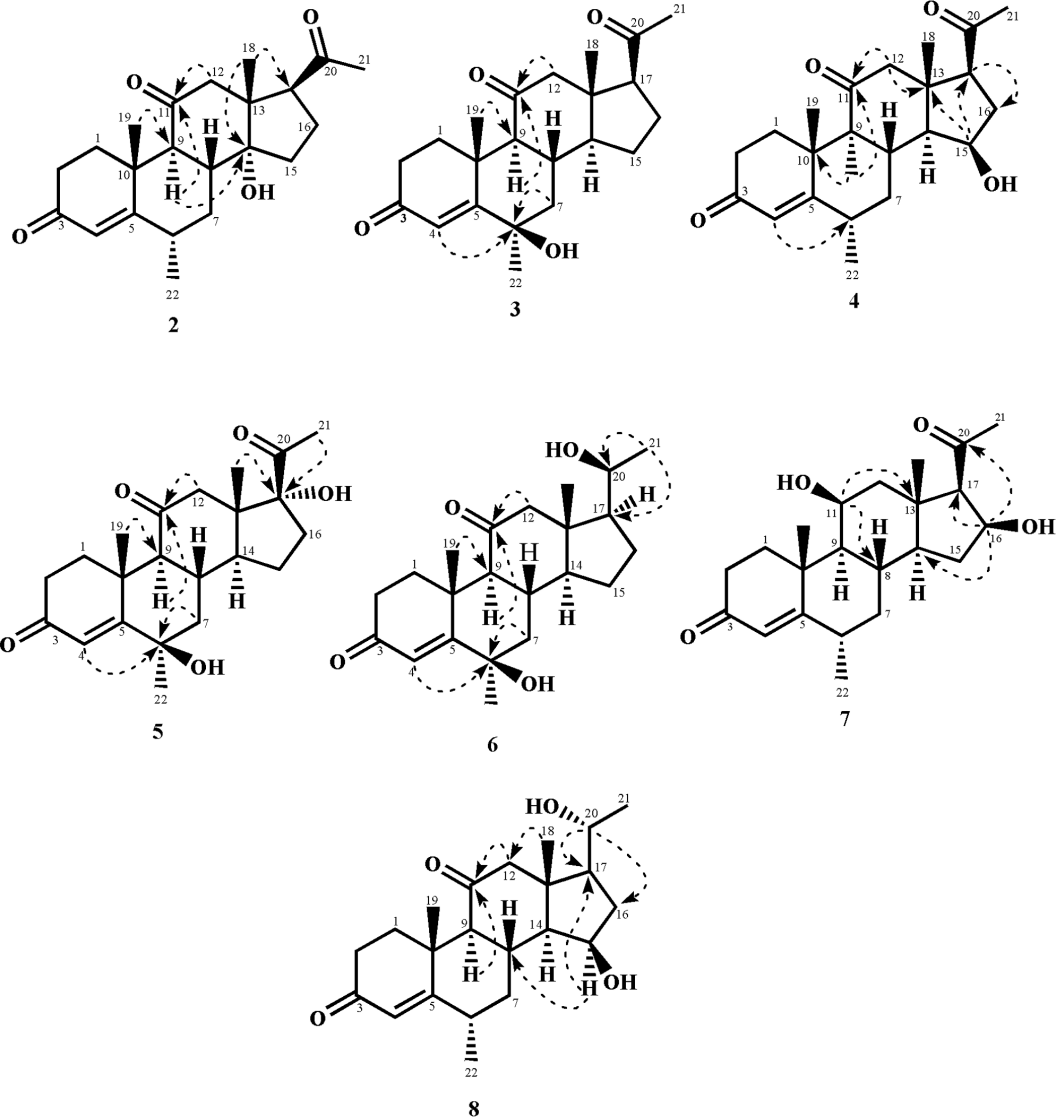
Key HMBC (H→C) correlations in new metabolites 2–8.

**Fig 5:**
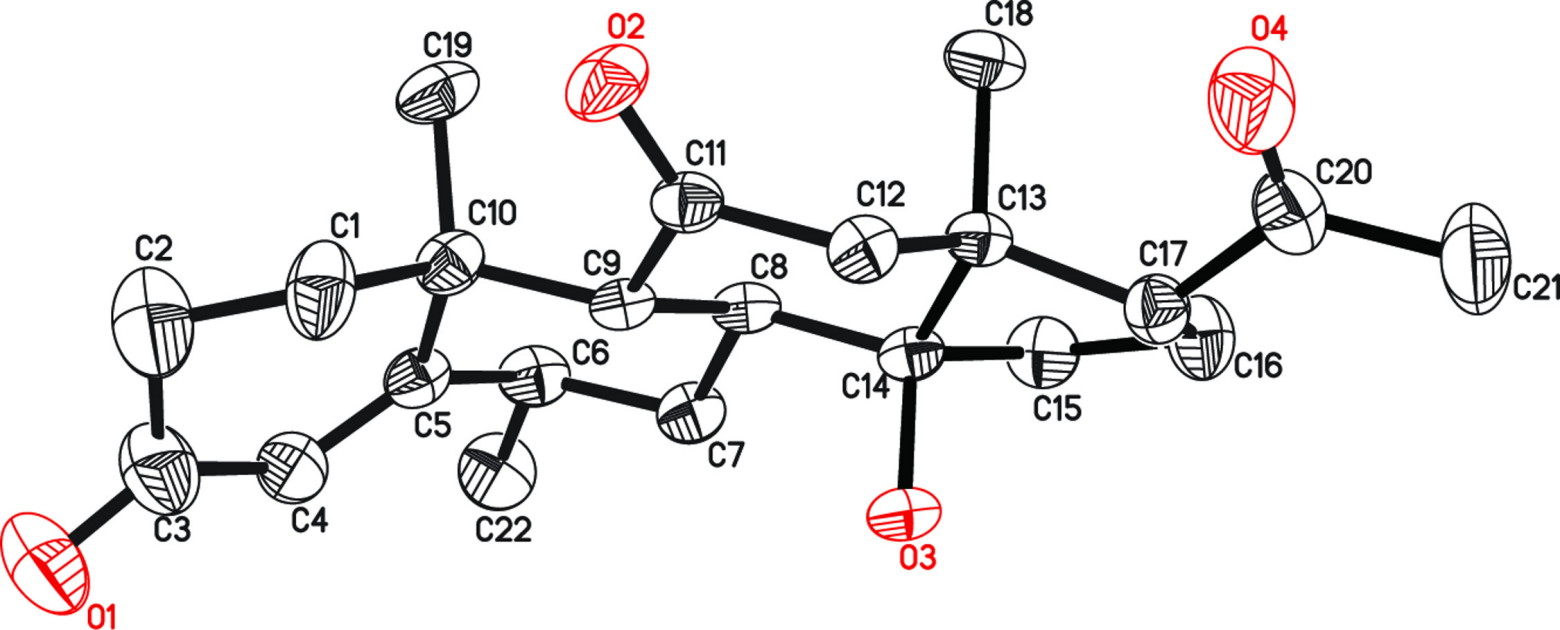
ORTEP Diagram of metabolite 2. Hydrogen atoms are omitted for clarity.

The HREI-MS of metabolite **2** showed an M^+^ peak at *m/z* 358.2128 indicating the formula C_22_H_30_O_4_ (calcd. 358.2139), consistent with eight degrees of unsaturation. The IR absorbance at 3466, 1700, and 1662 cm^-1^ indicated the presence of hydroxyl, ketonic carbonyl, and α,β-unsaturated ketonic carbonyl moieties, respectively. The ^1^H-NMR spectrum (Table 1) showed the C-12 methylene protons as AB doublets at δ 3.15 (*J*_12α, 12β_ = 12.3 Hz) and 2.35 (*J*_12β, 12α_ = 12.3 Hz), indicating an oxidation at C-11. The ^13^C-NMR spectrum (Table 2) further showed the presence of two additional quaternary carbons at δ 209.0 (C = O) and δ 83.5 (C-OH). The ^*2*^*J* HMBC correlations of H_2_-12 (δ 3.15, 2.35) and H-9 (δ 2.52) with a ketonic carbon (δ 209.0) and ^*3*^*J* correlation of H-19 (δ 1.39) with C-9 (δ 56.9) indicated the presence of a ketonic carbonyl at C-11. Furthermore, the presence of an OH group at C-14 was deduced from the ^*3*^*J* HMBC correlations of H-18 (δ 0.72) with C-14 (δ 83.5) and C-17 (δ 58.0) (Fig 4). The structure of compound **2** was unambiguously deduced by single-crystal X-ray diffraction analysis. The ORTEP diagram indicated that the molecule consists of four fused rings, *i*.*e*. A (C-1—C-5/C-10), B (C-5—C-10), C (C-8—C-9/C-11—C-14) and D (C-13—C-17). Ring A exists in a half chair conformation. *Trans* fused rings B, C, and D exist in chair/chair, and envelope conformations, respectively. The acetyl substituent, attached to C-17, adopts *pseudo equitorial* orientations. In contrast, the C-14 hydroxyl substituent has α (*axial*) orientation (Fig 5). Finally the new metabolite **2** was identified as 14α-hydroxy-6α-methylpregn-4-ene-3,11,20-trione.

**Fig 6:**
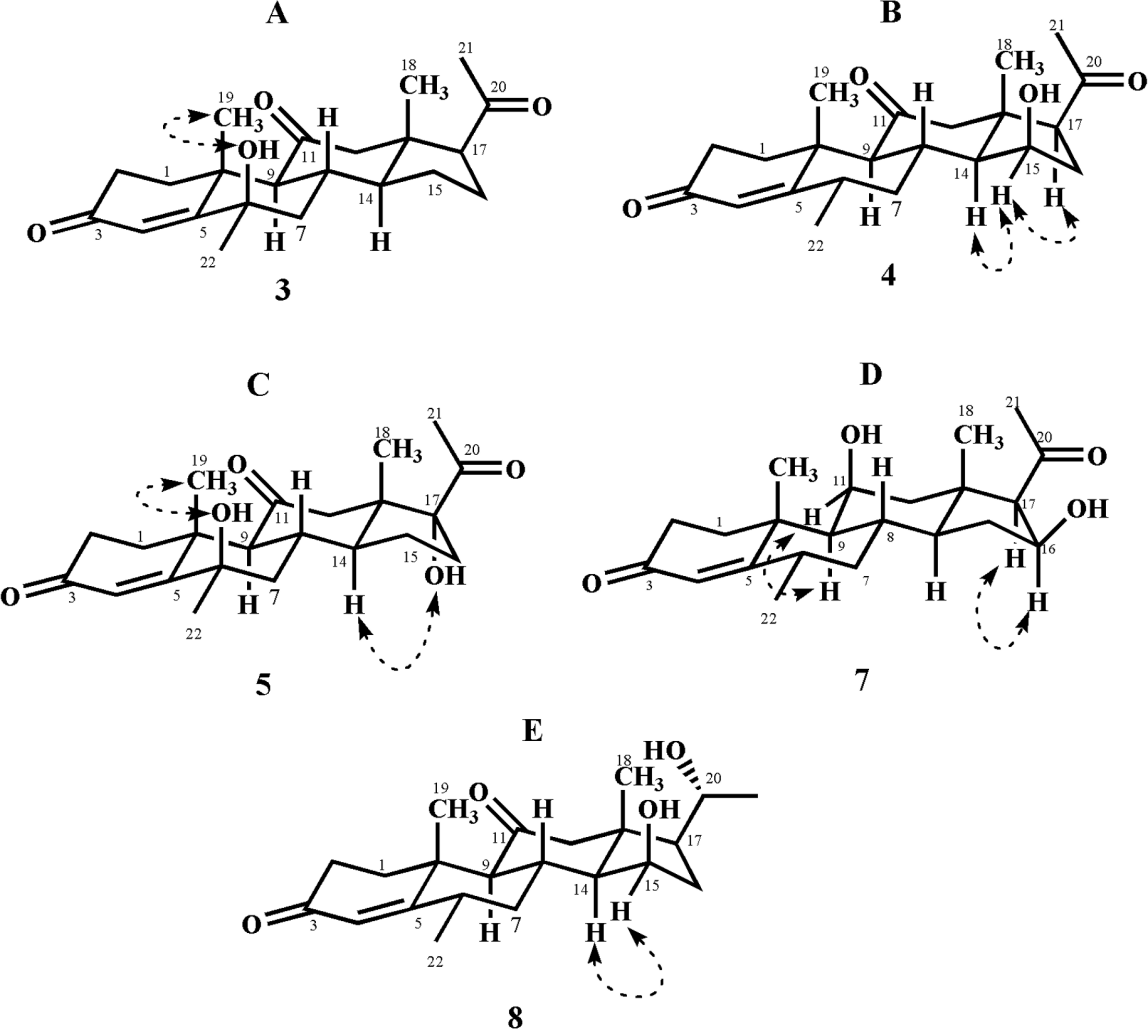
Key NOESY correlations in new metabolites (A) NOESY correlation in metabolite **3** supporting β-OH (*axial*) at C-6 (B) NOESY correlations in metabolite **4** supporting β- OH at C-15 (C) NOESY correlations in metabolite **5** supporting β-OH (*axial*) at C-6, and α-OH at C-17 (D) NOESY correlations in metabolites **7** supporting β-OH at C- 11(*axial*), and C-16; and (E) NOESY correlation in metabolite **8** supporting β-OH at C-15.

The HREI-MS of metabolite **3** supported the formula C_22_H_30_O_4_ [M^+^ = *m/z* 358.2115 (calcd. 358.2139)] with eight degrees of unsaturation. The IR spectrum showed the presence of hydroxyl (3479 cm^-1^), ketonic carbonyl (1707 cm^-1^), and α,β-unsaturated ketonic carbonyl groups (1678 cm^-1^). The ^1^H-NMR spectrum (Table 1) showed a downfield shift of H-9 signal (δ 2.70), and the appearance of two downfield AB doublets for H_2_-12 at δ 2.74 (*J*_12α,12β_ = 12.3 Hz) and 2.45 (*J*_12β,12α_ = 12.3 Hz) which indicated a ketonic carbonyl at C-11. Furthermore, the appearance of CH_3_-22 as a singlet (instead of a doublet) indicated the hydroxylation at vicinal C-6. The ^13^C-NMR spectrum (Table 2) showed two new quaternary carbon signals at δ 207.9 and 71.0. ^2^*J* HMBC correlations of H-9 (δ 2.70) and H_2_-12 (δ 2.74, 2.45) with δ 207.9. This supported the presence of a ketonic functionality at C-11. Similarly, ^3^*J* HMBC of H-4 (δ 6.00), and ^2^*J* correlations of H_2_-7 (δ 2.02, 1.35) with C-6 (δ 71.0) indicated the presence of an OH hydroxyl group at C-6 (Fig 4). The stereochemistry of C-6 was deduced as β on the basis of NOESY correlations (DMSO-*d*_*6*_) between OH (δ 4.88) and H-19 (δ 1.26) (Fig 6A). The structure of new metabolite **3** was thus deduced as 6β-hydroxy-6α-methylpregn-4-ene-3,11,20-trione.

**Fig 7:**
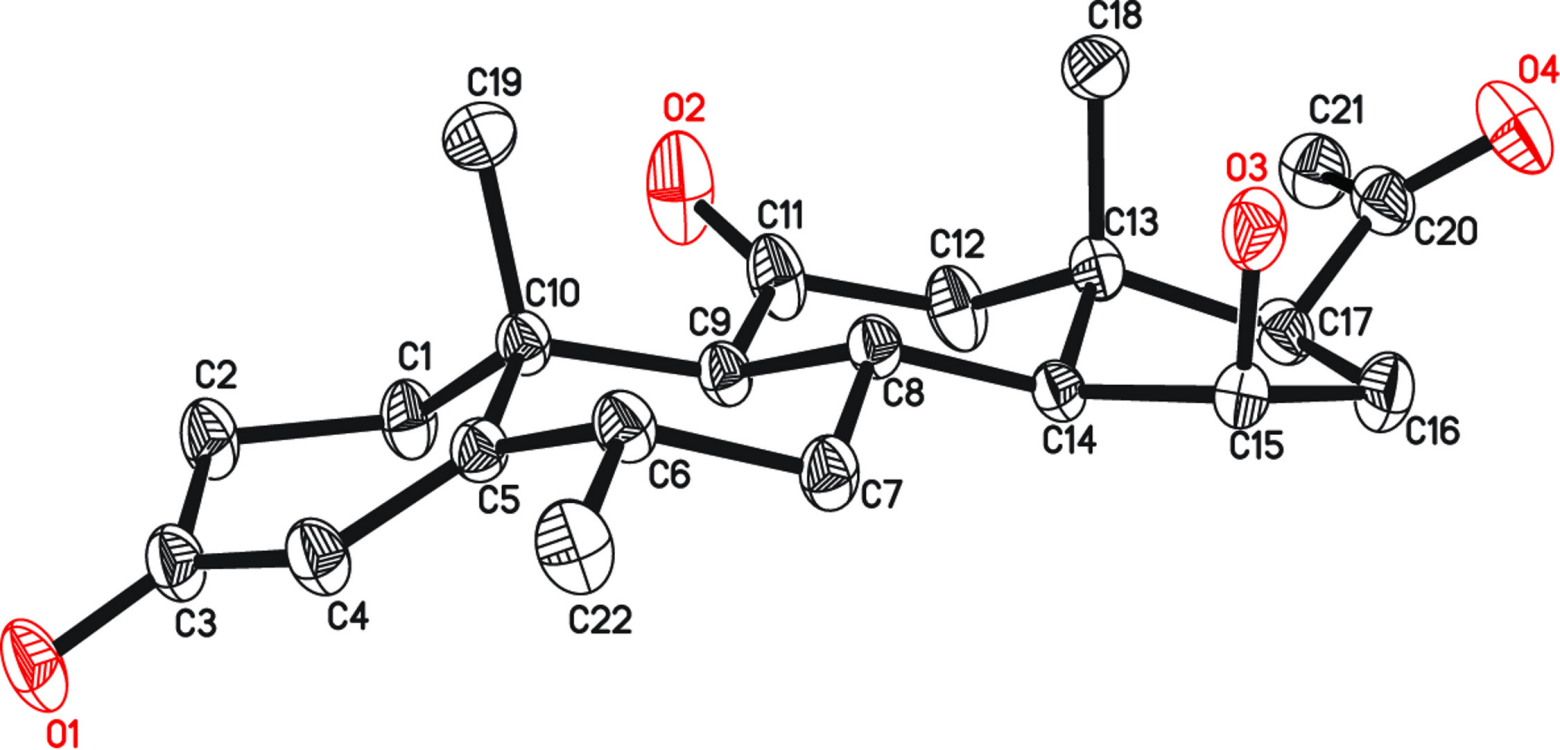
ORTEP Diagram of metabolite 4 representing final X-ray structure. Hydrogen atoms are omitted for clarity.

The HREI-MS of metabolite **4** supported the molecular formula C_22_H_30_O_4_ [M^+^ = *m/z* 358.2126 (Calcd. 358.2139)] with eight degrees of unsaturation. The IR absorptions at 3423, 1703, and 1658 cm^-1^ were due to the hydroxyl, ketonic carbonyl, and α,β-unsaturated ketonic carbonyl moieties, respectively. The ^1^H-NMR spectrum (Table 1) showed an additional downfield methine signal at δ 4.40, indicating hydroxylation at that position. The two AB doublets of H_2_-12 at δ 2.58 (*J*_12β,12α_ = 12.0 Hz) and 2.48 (*J*_12α,12β_ = 12.0 Hz), showed oxidation of the C-11 OH into a ketonic carbonyl. The ^13^C-NMR (Table 2) spectrum showed an additional downfield methine signal at δ 69.2, indicating that hydroxylation had occurred at that carbon. A new ketonic carbonyl carbon signal also appeared at δ 207.6 in the spectrum. The ^2^*J* HMBC correlations of H-9 (δ 1.97) with C-11 (δ 207.6) and C-10 (δ 38.6) supported a ketonic group at C-11. Furthermore, the ^3^*J* HMBC correlations of H-15 (δ 4.40) with C-13 (δ 46.6) and C-17 (δ 62.0) supported an OH at C-15 (Fig 4). NOESY correlations of H-15 (δ 4.40) with H-14 (δ 1.60) and H-17 (δ 2.66) supported the stereochemistry of OH group as C-15 as β (*pseudo axial*) (Fig 6B). Single-crystal X-ray diffraction analysis was carried out to unambiguously deduce the structure of metabolite **4**. The X-ray studies showed that the molecule consists of four fused rings, *i*.*e*. ring A (C-1—C-5/C-10), B (C-5—C-10), C (C-8—C-9/C-11—C-14) and D (C-13—C-17). Ring A exists in a half-chair conformation. *Trans* fused rings B, C and D exist in chair, chair and envelope conformations, respectively. The acetyl substituent attached at C17 adopts a *pseudo axial* orientation, whereas the OH at C-15 is β (*pseudo-axial*) (Fig 7). Compound **4** was characterized as a new metabolite, 15β-hydroxy-6α-methylpregn-4-ene-3,11,20-trione.

The HREI-MS of metabolite **5** showed the M^+^ peak at *m/z* 374.2068, in agreement with the formula C_22_H_30_O_5_ (calcd. 374.2088) with eight degrees of unsaturation. The IR spectrum showed absorptions at 3450 and 1699 cm^-1^ for hydroxyl and α,β-unsaturated ketonic carbonyl moieties, respectively. The ^1^H-NMR spectrum (Table 1) showed a downfield shift of H-9 signal (δ 2.07), and the presence of AB doublets of C-12 methylene protons at δ 2.97 (*J*_12β, 12α_ = 12.6 Hz), and 2.12 (*J*_12α, 12β_ = 12.6 Hz), supported the presence of a ketonic carbonyl at C-11. Secondly, the appearance of CH_3_-22 as a singlet indicated a hydroxylation at the C-6 position. The ^13^C-NMR spectrum (Table 2) showed three additional quaternary carbons resonating at δ 71.2, 212.3, and 90.0. ^2^*J* HMBC correlations of H-9 (δ 2.07) and H_2_-12 (δ 2.97, 2.12) with C-11 (δ 212.3) supported the presence of a ketonic carbonyl at C-11. The position of the hydroxyl group at C-6 was inferred from ^3^*J* and ^2^*J* HMBC correlations of H-4 (δ 5.98) and H_2_-7 (δ 1.98, 1.45) with C-6 (δ 71.2). An OH at C-17 was deduced on the basis of ^3^*J* HMBC correlations of H-18 (δ 0.56, s), and H-21 (δ 2.17) with C-17 (δ 90.0) (Fig 4). The stereochemistry of the OH-group at C-6 was deduced as β on the basis of NOESY correlations (DMSO-*d*_*6*_) between OH (δ 4.88) and H-19 (δ 1.25). Similarly, the stereochemistry of the OH at C-17 was deduced as *α* on the basis of NOESY correlation (DMSO-*d*_*6*_) between OH (δ 5.64) and H-14 (δ 2.07) (Fig 6C). Compound **5** was thus identified as a new metabolite (6β,17α-dihydroxy-6α-methylpregn-4-ene-3,11,20-trione).

**Fig 8:**
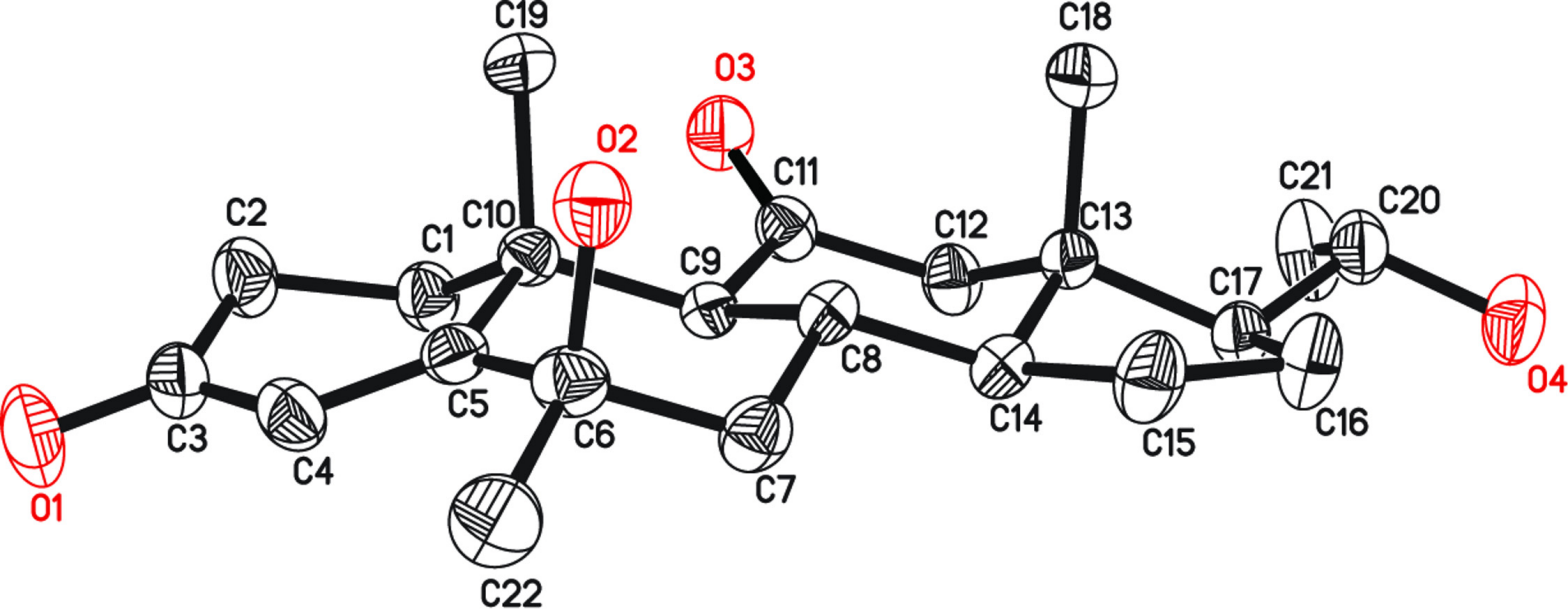
ORTEP Diagram of metabolite 6, representing final X-ray structure.

The ESI-MS of **6** exhibited the [M+H]^+^ at *m/z* 361.2382 (C_22_H_32_O_4_+H, requires 361.2378), 16 a.m.u. greater than that of substrate **1**, suggesting the multiple oxidation of the substrate. The IR spectrum showed absorptions at 3445, 1742, and 1662 cm^-1^ for hydroxyl, ketonic carbonyl, and α,β-unsaturated ketonic carbonyl moieties, respectively. The downfield shift of H-9 (δ 1.85) and the appearance of the C-12 methylene as AB doublets at δ 2.49 (*J*_12α, 12β_ = 12.5 Hz), and 2.22 (*J*_12β. 12α_ = 12.5 Hz) indicated oxidation at C-11 (Table 1). Furthermore, the CH_3_-22 appeared as a singlet, due to hydroxylation at C-6. The appearance of CH_3_-21 as a doublet, supported the reduction of the C-20 ketonic functionality into an OH. The ^13^C-NMR spectrum (Table 2) showed two new quaternary carbons *i*.*e*. C-11 (δ 209.3) and C-6 (δ 71.1). The presence of new methine OH-containing C-20 (δ 69.4) was deduced from upfield chemical shift of vicinal C-21 (δ 23.4). In the HMBC spectrum, ^2^*J* correlations of H-9 (δ 1.85) and H_2_-12 (δ 2.49, 2.22) with C-11 (δ 209.3) supported a ketonic group at C-11. Furthermore, ^3^*J* correlation of H-19 (δ 1.60) with C-9 (δ 62.3) also supported C-11 C = O. ^3^*J* HMBC correlation of H-4 (δ 6.0), and ^2^*J* correlation of H_2_-7 (δ 2.01, 1.33) with C-6 (δ 71.1), were used to place an OH at C-6. ^2^*J* and ^3^*J* HMBC correlations of H-21 (δ 1.20) with C-20 (δ 69.4) and C-17 (δ 57.0), respectively, were used to deduce the position of an OH-bearing methine at C-20 (Fig 4). Single-crystal X-ray diffraction analysis of metabolite **6** indicated that the molecule consists of four fused rings *i*.*e*. rings A (C-1—C-5/C-10), B (C-5—C-10), C (C-8—C-9/C-11—C-14). and D (C-13—C-17). The stereochemistry at C-20 appeared as *S*, and the hydroxyl group at C-6 was β oriented (Fig 8). The structure of the new metabolite **6** was finally characterized as 6β,20*S*-dihydroxy-6α-methylpregn-4-ene-3,11-dione.

The ESI-MS of compound **7** showed the [M+H]^+^ at *m/z* 361.2374 (C_22_H_32_O_4_+H, calcd. 361.2378), 16 a.m.u. greater than the substrate **1**. The IR absorptions appeared at 3402, 1703, and 1662 cm^-1^ were for the hydroxyl, ketonic carbonyl, and α,β-unsaturated ketonic carbonyl moieties, respectively. The ^1^H-NMR spectrum (Table 1) showed a downfield signal at δ 4.72 (t, *J*_16α, 15αβ/17α_ = 7.3 Hz) due to the hydroxylation of the substrate **1**. The ^13^C-NMR spectrum (Table 2) showed a new methine carbon at C-16 (δ 72.5) as compared to the substrate **1**. An OH group at C-16 was placed on the basis of ^3^*J* HMBC correlations between H-16 (δ 4.72) and C-14 (δ 56.4) and C-20 (δ 210.5) (Fig 4). The β (*pseudo equatorial*) orientation of OH group at C-16 was deduced on the basis of NOESY correlation between geminal H-16 (δ 4.72) and H-17 (δ 2.49). The stereochemistry of the newly generated OH at C-11 was also deduced to be β (*axial*) on the basis of NOESY correlation between H-11 (δ 4.34) and H-9 (δ 1.10) (Fig 6D). Metabolite **7** was hence identified as 11β,16β -dihydroxy-6α-methylpregn-4-en-3,20-dione.

**Fig 9:**
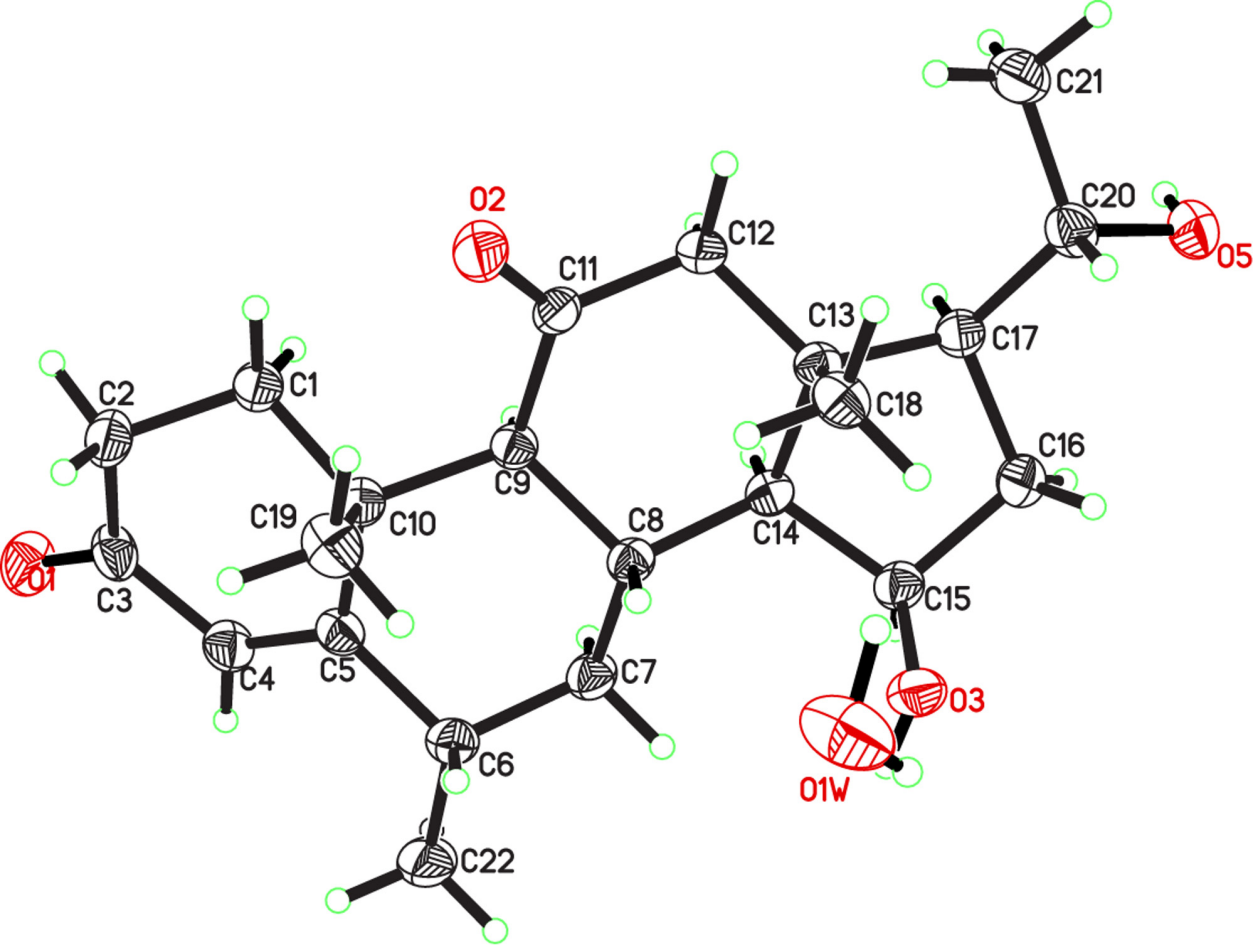
ORTEP Diagram of metabolite 8 is representing final X-ray structure. Water of solvation is visible.

The HREI-MS of compound **8** exhibited the M^+^ at *m/z* 360.2295 which supported the formula as C_22_H_32_O_4_ (calcd. 360.2295), with seven degrees of unsaturation. The IR absorptions at 3431, 1703, and 1662 cm^-1^ were due to hydroxyl, ketonic carbonyl, α,β-unsaturated ketonic carbonyl moieties, respectively. The ^1^H-NMR spectrum (Table 1) showed two downfield methine proton signals at δ 4.32 (t, *J*_15α,14α/16α_ = 5.7 Hz), and 3.66 (m), due to the hydroxylation at the two sites. The appearance of an additional doublet for H_3_-21 at δ 1.11 (*J*_21, 20β_ = 6.3 Hz) indicated the reduction of the vicinal C-20 ketonic carbonyl into a CH-OH. The ^13^C-NMR spectrum (Table 2) showed a new quaternary carbon signal at δ 211.9, placed at C-11 based on the downfield shifts of neighboring C-9 (δ 63.6), and C-12 (δ 58.8). Two new methine signals appeared at C-15 (δ 69.4) and C-20 (δ 70.2). ^3^*J* HMBC correlations of H-15 (δ 4.32) with C-8 (δ 33.8) and C-17 (δ 58.2) further indicated the presence of an OH at C-15. The presence of an OH at C-20 (δ 70.2) was deduced on the basis of ^2^*J* and ^3^*J* HMBC correlations of H-20 (δ 3.66) with C-17 (δ 58.2) and C-16 (δ 40.8), respectively. ^3^*J* HMBC correlation of H-18 with C-12 (δ 58.8) further supported a carbonyl at C-11 (Fig 4). The stereochemistry of the C-15 OH was assigned to be β (*pseudo axial*) based on NOESY correlations between H-15 (δ 4.32) and H-14 (δ 1.62) (Fig 6E). Single-crystal X-ray diffraction analysis indicated that the molecule consists of four fused rings *i*.*e*. ring A (C-1—C-5/C-10), B (C-5—C-10), C (C-8—C-9/C-11—C-14) and D (C-13—C-17). The stereochemistry at C-20 appeared as *R* and the hydroxyl group at C-15 was β oriented, respectively (Fig 9). The structure of the new metabolite **8** was therefore deduced as 15β,20*R*-dihydroxy-6α-methylpregn-4-en-3,11-dione.

#### Anti-inflammatory activity

To explore the anti-inflammatory effects of medrysone (**1**), and its metabolites **2–8**, different assays, such as phagocyte oxidative burst, T-cell proliferation, and cytokine inhibition assays were employed.

#### Effect on phagocytes oxidative burst

The production of reactive oxygen species (ROS) plays a key role in the development of many inflammatory disorders. This includes endothelial dysfunction and tissue injury by an enhanced ROS generation by polymorphonuclear neutrophils (PMNs) at the site of inflammation. In our study the luminol-enhanced chemiluminescence assay was used for the measurement of the production of ROS. Metabolites **2–8** were evaluated for the inhibition of the production of intracellular ROS (OH, O_2_^˗^, H_2_O_2_, HOCl) by using luminol as a probe and zymosan as an activator [34–36]. The results indicated that all the tested compounds were found to be inactive, except metabolite **6** (IC_50_ **=** 30.3 ± 8.8 μg/mL), which showed moderate activity against the zymosan-induced oxidative burst in PMNs (Table 3).

**Table 3:** Effect of compounds 1–8 on luminol enhanced oxidative burst using zymosan activated PMNs.

Compounds	IC_50_^a^_μ_g/mL
**1**	>100
**2**	>100
**3**	>100
**4**	>100
**5**	>100
**6**	30.3 ± 8.8
**7**	>100
**8**	>100
**Ibuprofen** (Standard)	2.5 ± 0.6

^a^The IC_50_ values were obtained using various concentrations of test compounds, and readings are presented as mean ± SD of triplicates.

#### Effect on T-cell proliferation

T-Lymphocytes are the main cells of adaptive immune responses and are known to play a central role in pathogenesis of various autoimmune diseases. During chronic inflammation the cytokines secreted by activated T-cells are known to activate and proliferate the population of various other immune cells. They are also involved in graft rejection process during transplantation, where they destroy the graft directly through cell mediated lysis or indirectly by enhancing antibody production or by activating complement. Hence the inhibition of proliferation of T-cells provides strong immunosuppressive approach for the treatment of various autoimmune diseases and transplantation rejection [37]. During this study, medrysone (**1**), and its metabolites **2–8** were evaluated for the inhibition of T-cell proliferation by using PBMCs, activated with PHA. The results indicated that compounds **1**, **4**, **5**, **7**, and **8** possess strong inhibitory activity with an IC_50_ values between < 0.2 to 10.4 μg/mL. Among them compound **7** was the most potent inhibitor (IC_50_ < 0.2 μg/mL) of T-cell proliferation. Compounds **2**, **3**, and **6** were found to be moderately active with an IC_50_ ranges between 14.6–20.0 μg/mL as compared with the standard drug prednisolone (IC_50_ < 3.1 μg/mL) (Table 4).

**Table 4:** Effect of compounds 1–8 on PHA activated T-cells proliferation.

Compounds	IC_50_^a^ μg/mL
**1**	2.9 ± 0.04
**2**	20.0 ± 0.9
**3**	14.6 ± 2.1
**4**	9.2±0.7
**5**	1.2 ± 0.02
**6**	15.2 ± 2.4
**7**	<0.2
**8**	10.4 ± 0.42
**Prednisolone** (Standard)	<3.1

^a^The IC_50_ values were obtained using various concentrations of test compounds, and readings are presented as mean ± SD of triplicates.

### Effect on TNF-α

Cytokines are regulators of host responses to infection and inflammation, while the presence of pro-inflammatory cytokines worsens the disease condition. TNF-α is a pro-inflammatory cytokine, which promotes systemic inflammation. Blocking of TNF-α during overwhelming infection can improve the disease condition. During our studies, the substrate and transformed products **2–8** were evaluated against the production of TNF-α [38]. Among them, compound **1** (IC_50_ **=** 30.54 ± 1.69 μg/mL), and its metabolite **7** (IC_50_ **=** 28.6 ± 11.5 μg/mL) were found to be moderate inhibitors of TNF-α production (Table 5).

**Table 5:** The table represents % effect of compounds 1–8 (25 μg/mL) on TNF-α produced by LPS activated THP-1 cells.

Compounds	TNF-α % inhibition^a^ (μg/mL)
**1**	30.54 ± 1.69
**2**	-4.04 ± 0.2
**3**	-2.69 ± 5.08
**4**	-7.49 ± 1.27
**5**	6.44 ± 0.21
**6**	4.4 ± 9.6
**7**	28.6 ± 11.5
**8**	-8.23 ± 2.75

^a^Results are presented as mean ± SD of triplicates. The level of TNF-α was monitored using ELISA kits.–ve sign = indicates increase in the cytokine level as compared to activated cells without test compound.

These results have helped us to identify compounds with potential anti-inflammatory activity. Compounds **1–8** were analyzed for their immunomodulatory effect on different parameters of both innate and adaptive immune responses, including their effect on generation of ROS, proliferation of T-cells, and production of pro-inflammatory cytokine TNF-α. During an innate immune response, the phagocytes release several chemical mediators like reactive oxygen species (ROS) and cytokines, which perpetuate the inflammatory process and activate the adaptive immune responses. During the current study, compound **6** exhibited moderate inhibitory activity on ROS produced from professional phagocytes, activated with the serum opsonized zymosan (as an antigen) by myeloperoxidase dependent pathway. It also showed moderate inhibition of proliferation of PHA activated T-cells. Compounds **1**, **4**, **5**, **7**, and **8** strongly inhibited the PHA activated proliferation of T-cells, whereas compounds **2**, **3**, and **6** were found to be the moderate inhibitors. Compound **7** was found to be the most potent inhibitor of T-cells proliferation from this group. These results indicate that all compounds have the potential to inhibit cellular immune responses and might be useful in suppressing various chronic inflammatory and autoimmune disorders as well as for treatment of transplantation rejection. Among all compounds, the compounds **1**, and **7** were also found to inhibit pro-inflammatory cytokine TNF-α produced from LPS activated macrophages. The blockade of TNF-α proved to be beneficial in many pathological conditions including rheumatoid arthritis (RA), inflammatory bowel disease (IBD), and psoriasis. All compounds showed their suppressive effects on various parameters of innate and adaptive immune responses, and can provide valuable insight for the treatment of different chronic inflammatory and autoimmune illnesses.

### Cytotoxicity activity

Substrate **1** and its metabolites **2**–**8** were evaluated for their cytotoxicity against HeLa (human epithelial carcinoma), PC3 (prostate cancer), and 3T3 (mouse fibroblast) cells. All the compounds were found to be non-cytotoxic against above mentioned cell lines.

## Conclusion

In conclusion, this is the first report of the fungal transformation of steroidal anti-inflammatory drug medrysone (**1**) into several new derivatives **2**–**8** with *C*. *blakesleeana* (ATCC 8688a), *N*. *crassa* (ATCC 18419), and *R*. *stolonifer* (TSY 0471). Through this study we identified an efficient route towards the synthesis of C-6β, 11β, 14α, 15β, 16β, and 20β oxidation products. Single-crystal X-ray diffraction analyses were performed to unambiguously deduce the structures of metabolites **2**, **4**, **6**, and **8**. Among all the metabolites, compound **6** (IC_50_ **=** 30.3 μg/mL) showed moderate inhibitory activity against the zymosan-induced oxidative burst in human whole blood cells whereas rest of the compounds were found to be inactive. When tested for their effects on proliferation of T-cells, compounds **1**, **4**, **5**, **7**, and **8** showed a strong inhibitory activity against these cells. Compound **7** (IC_50_ < 0.2 μg/mL) was the most potent inhibitor of T-cell proliferation. The compounds **2**, **3**, and **6** showed moderate levels of inhibition with an IC_50_ values ranges between 14.6 to 20.0 μg/mL. When tested for their effect on production of pro-inflammatory cytokine, TNF-α, compounds **1**, and **7** showed moderate levels of inhibition while remaining compounds showed no inhibitory activity. All the compounds were found to be non-toxic when tested on 3T3 (mouse fibroblast) cells and showed no activity when tested against HeLa (human epithelial carcinoma), and PC3 (prostate cancer) cell lines. The work presented here can be helpful for the study of *in vivo* metabolism of medrysone (**1**), as well as for the identification of new anti-inflammatory agents, based on bio-catalysed structural transformation of various steroids.

## Supporting Information

S1 File The MS and NMR spectra of compound 1. (PDF)

S2 File The MS and NMR spectra of compound 2. (PDF)

S3 File The MS and NMR spectra of compound 3. (PDF)

S4 File The MS and NMR spectra of compound 4. (PDF)

S5 File The MS and NMR spectra of compound 5. (PDF)

S6 File The MS and NMR spectra of compound 6. (PDF)

S7 File The MS and NMR spectra of compound 7. (PDF)

S8 File The MS and NMR spectra of compound 8. (PDF)

## References

[1] Yang C, Fan H, Yuan Y, Gao J. Microbial transformation of pregnane-3β,16β,20-triol by *Cunninghamella echinulate*. Chinese J Chem. 2013; 31: 127–131.

[2] Bhatti HN, Khera RA. Biological transformations of steroidal compounds: a review. Steroids. 2012; 77: 1267–1290. 10.1016/j.steroids.2012.07.018 22910289

[3] Habibi Z, Yousefi M, Ghanian S, Mohammadi M, Ghasemi S. Biotransformation of progesterone by *Absidia griseolla* var. *igachii* and *Rhizomucor pusillus*. Steroids. 2012; 77: 1446–1449. 10.1016/j.steroids.2012.08.010 22974825

[4] Świzdor A, Panek A, Milecka-Tronina N. Microbial Baeyer-Villiger oxidation of 5α-steroids using *Beauveria bassiana*. A stereochemical requirement for the 11α-hydroxylation and the lactonization pathway. Steroids. 2014; 82: 44–52. 10.1016/j.steroids.2014.01.006 24486796

[5] Gao JM, Shen JW, Wang JY, Yang Z, Zhang AL. Microbial transformation of 3β-acetoxypregna-5,16-diene-20-one by *Penicillium citrinum*. Steroids. 2011; 76: 43–7. 10.1016/j.steroids.2010.08.006 20801138

[6] Peterson DH, Murray HC, Epstein SH, Reineke LM, Weintraub A, Meister PD, et al Microbiological oxygenation of steroids. I. introduction of oxygen at carbon-11 of progesterone. J Am Chem Soc. 1952; 74: 5933–5936.

[7] Smith C, Wahab AT, Khan MS, Ahmad MS, Farran D, Choudhary MI, et al Microbial transformation of oxandrolone with *Macrophomina phaseolina* and *Cunninghamella blakesleeana*. Steroids. 2015; 102: 39–45. 10.1016/j.steroids.2015.06.008 26095204

[8] Ahmad MS, Zafar S, Bibi M, Bano S, Atia-tul-Wahab, Atta-ur-Rahman, et al Biotransformation of androgenic steroid mesterolone with *Cunninghamella blakesleeana* and *Macrophomina phaseolina*, Steroids. 2014; 82: 53–9. 10.1016/j.steroids.2014.01.001 24462640

[9] Khan NT, Zafar S, Noreen S, Al Majid AM, Al Othman ZA, Al-Resayes SI, et al Biotransformation of dianabol with the filamentous fungi and β-glucuronidase inhibitory activity of resulting metabolites. Steroids. 2014; 85: 65–72. 10.1016/j.steroids.2014.04.004 24755238

[10] Choudhary MI, Erum S, Atif M, Malik R, Khan NT, Atta-ur-Rahman. Biotransformation of (20S)-20-hydroxymethylpregna-1,4-dien-3-one by four filamentous fungi. Steroids. 2011; 76: 1288–1296. 10.1016/j.steroids.2011.06.007 21762714

[11] Zafar S, Bibi M, Yousuf S, Choudhary MI. New metabolites from fungal biotransformation of an oral contraceptive agent: methyloestrenolone. Steroids. 2013; 78: 418–425. 10.1016/j.steroids.2013.01.002 23357433

[12] Baydoun E, Karam M, Atia-tul-Wahab, Khan MS, Ahmad MS, Samreen, et al Microbial transformation of nandrolone with *Cunninghamella echinulata* and *Cunninghamella blakesleeana* and evaluation of leishmaniacidal activity of transformed products. Steroids. 2014; 88: 95–100. 10.1016/j.steroids.2014.06.020 25014252

[13] Choudhary MI, Yousuf S, Samreen, Shah SA, Ahmed S, Atta-ur-Rahman. Biotransformation of physalin H and leishmanicidal activity of its transformed products. Chem Pharm Bull. 2006; 54: 927–930. 16819205 10.1248/cpb.54.927

[14] Azizuddin Choudhary MI. Microbial transformation of dydrogesterone by *Gibberella fujikuroi*. J Biochem Tech. 2012; 3: 336–338.

[15] Choudhary MI, Sultan S, Jalil S, Anjum S, Rahman AA, Fun HK, et al Microbial transformation of mesterolone. Chem Biodivers. 2005; 2: 392–400. 17191988 10.1002/cbdv.200590019

[16] Zafar S, Yousuf S, Kayani HA, Saifullah, Khan S, Al-Majid AM, et al Biotransformation of oral contraceptive ethynodiol diacetate with microbial and plant cell cultures. Chem Cent J. 2012; 6: 109 10.1186/1752-153X-6-109 23021311 PMC3496622

[17] Zafar S, Choudhary MI, Dalvandi K, Mahmood U, Ul-Haq Z. Molecular docking simulation studies on potent butyrylcholinesterase inhibitors obtained from microbial transformation of dihydrotestosterone. Chem Cent J. 2013; 7: 164 10.1186/1752-153X-7-164 24103815 PMC4126177

[18] Al-Maruf MA, Khan NT, Saki MAA, Choudhary MI, Ali MU, Islam MA. Biotransformation of 11-ketoprogesterone by filamentous fungus, *Fusarium lini*. J Sci Res. 2011; 3: 347–356.

[19] Choudhary MI, Sultan S, Khan MT, Yasin A, Shaheen F, Atta-ur-Rahman. Biotransformation of (+)-androst-4ene-3,17-dione. Nat Prod Res. 2006; 18: 529–535. 10.1080/14786410310001620628 15595610

[20] Choudhary MI, Siddiqui ZA, Musharraf SG, Nawaz SA, Atta-ur-Rahman. Biotransformation of (+)-androst-4-ene-3,17-dione. Nat Prod Res. 2005; 19: 311–7. 15938135

[21] Choudhary MI, Azizuddin, Atta-ur-Rahman. Microbial transformation of danazol. Nat Prod Lett. 2002; 16: 101–6. 11990425 10.1080/10575630290019994

[22] Choudhary MI, Nasir M, Khan SN, Atif M, Ali RA, Khalil SM, et al Microbial hydroxylation of hydroxyprogesterones and α-glucosidase inhibition activity of their metabolites. Z Naturforsch. 2007; 62: 593–599.

[23] Siemens A. SMART and SAINT. Madison: Siemens Analytical X-ray Instruments Inc; 1996.

[24] Altomare A, Cascarano G, Giacovazzo C, Guagliardi A. Completion and refinement of crystal structures with SIR92. J Appl Cryst. 1993; 26: 343–50

[25] Sheldrick GM. SHELXTL-PC (Version 5.1). Madison: Siemens Analytical Instruments, Inc; 1997.

[26] Johnson CK. ‘ORTEPII’, Report ORNL-5138. Oak Ridge National Laboratory, Tennessee, USA; 1976

[27] Betts RE, Walters DE, Rosazza JP. Microbial transformations of antitumor compounds. Conversion of acronycine to 9-hydroxyacronycine *by Cunninghamella echinulata*. J Med Chem. 1974; 17: 599–602. 4829940 10.1021/jm00252a006

[28] Helfand SL, Werkmeister J, Roder JC. Chemiluminescence response of human natural killer cells. The relationship between target cell binding, chemiluminescence and cytolysis. J Exp Med. 1982; 156: 492–505. 6178787 10.1084/jem.156.2.492 PMC2186753

[29] Nielsen M, Gerwien J, Nielsen M, Geisler C, Röpke C, Svejgaad A, et al MHC class II ligation induces CD58 (LFA-3)-mediated adhesion in human T cells. Exp Clin Immunogenet. 1998; 15: 61–68. 9691200 10.1159/000019055

[30] Dimas K, Demetzos C, Marsellos M, Sotiriadou R, Malamas M, Kokkinopoulos D. Cytotoxic activity of labdane type diterpenes against human leukemic cell lines *in vitro*. Planta Med. 1998; 64: 208–211. 9581515 10.1055/s-2006-957410

[31] Baydoun E, Bano S, Atia-tul-Wahab, Jabeen A, Yousuf S, Mesaik A, et al Fungal transformation and T-cell proliferation inhibitory activity of melengestrol acetate and its metabolite. Steroids. 2014; 86: 56–61. 10.1016/j.steroids.2014.04.012 24793568

[32] Khan NT, Bibi M, Yousuf S, Qureshi IH, Atta-Ur-Rahman N, Al-Majid AM, et al Synthesis of some potent immunomodulatory and anti-inflammatory metabolites by fungal transformation of anabolic steroid oxymetholone. Chem Cent J. 2012; 6: 153 10.1186/1752-153X-6-153 23237028 PMC3740782

[33] Choudhary MI, Azizuddin, Jalil S, Musharraf SG, Atta-ur-Rahman. Fungal transformation of dydrogesterone and inhibitory effect of its metabolites on the respiratory burst in human neutrophils. Chem Biodivers. 2008; 5: 324–331. 10.1002/cbdv.200890030 18293446

[34] Mittal M, Siddiqui MR, Tran K, Reddy SP, Malik AB. Reactive oxygen species in inflammation and tissue injury. Antioxid Redox Signal. 2014; 20: 1126–1167. 10.1089/ars.2012.5149 23991888 PMC3929010

[35] Ralee S, Özlem Y. The role of reactive-oxygen-species in microbial persistence and inflammation. Int J Mol Sci. 2011; 12: 334–352. 10.3390/ijms12010334 21339989 PMC3039955

[36] Mesaik MA, Jabeen A, Halim SA, Begum A, Khalid AS, Asif M, et al In silico and *in vitro* immunomodulatory studies on compounds of *Lindelofia stylosa*. Chem Biol Drug Des. 2012; 79: 290–299. 10.1111/j.1747-0285.2011.01310.x 22181857

[37] Krirger NR, Yin DP, Fathman CG. CD4+ but not CD8+ cells are essential for allorejection. J Exp Med. 1996; 184: 2013–2018. 8920888 10.1084/jem.184.5.2013 PMC2192869

[38] Dinarello CA. Pro-inflammatory cytokines. Chest. 2000; 118: 503–508. 10936147 10.1378/chest.118.2.503

