# Supplementary material for: New Anti-Inflammatory Metabolites by Microbial Transformation of Medrysone
Source: PLoS One. 2016 Apr 22;11(4):e0153951. doi: 10.1371/journal.pone.0153951 (PMC4841542; doi:10.1371/journal.pone.0153951)
Supplement: S1 File — (PDF) [file pone.0153951.s001.pdf]

Date Run: 03-08-2012 (Time Run: 11:12:03)

COMPOUND 1

Instrument: JEOL MSRoute

Inlet: Direct Probe

Ionization mode: EI+

Scan: 11 R.T.: .4

Base: m/z 138; 86.7%FS TIC: 15261897 (Max Inten : 909503) #Ions: 247

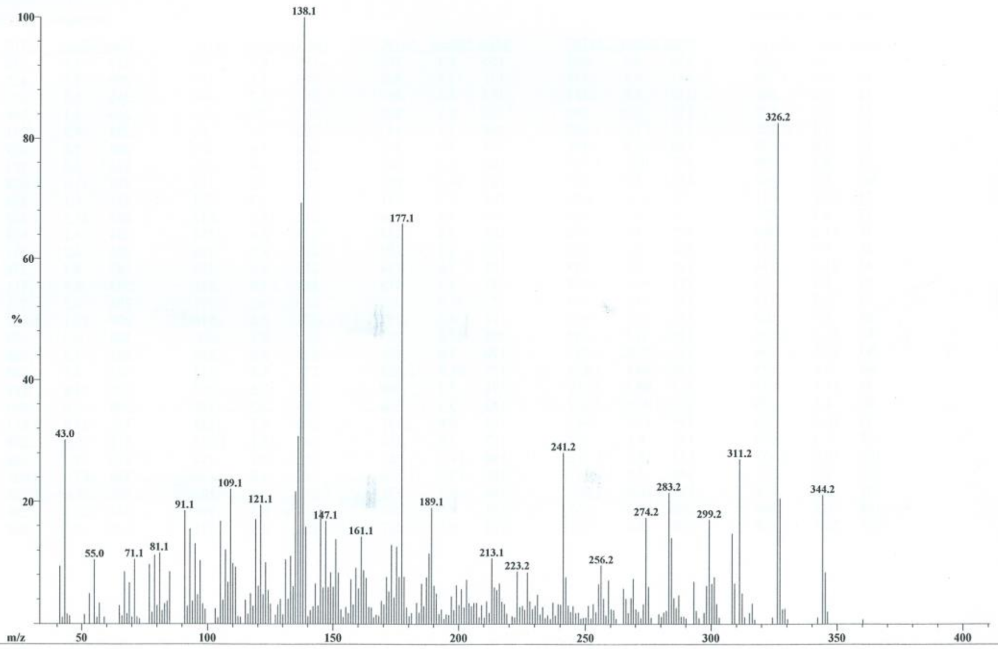

COMPOUND 1
HREI-MS

Mass	Relative Intensity	Theoretical Mass	Delta [ppm]	Delta [mmu]	RDB	Composition
229.1558	1.1433	229.1592	-15.1	-3.5	6.5	$C_{16} H_{21} O_1$
230.1647	1.1235	230.1671	-10.2	-2.4	6.0	$C_{16} H_{22} O_1$
230.9856	10.1973					
235.1461	2.3050	235.1487	-10.9	-2.6	9.5	$C_{18} H_{19}$
235.9903	1.5915	235.9898	2.1	0.5	18.0	$C_{17} O_2$
237.1281	6.4994	237.1279	0.6	0.1	9.5	$C_{17} H_{17} O_1$
238.1317	1.6528	238.1358	-17.0	-4.1	9.0	$C_{17} H_{18} O_1$
238.1719	1.5974	238.1722	-1.2	-0.3	8.0	$C_{18} H_{22}$
239.1445	8.5158	239.1436	3.7	0.9	8.5	$C_{17} H_{19} O_1$
240.1504	3.5755	240.1514	-4.3	-1.0	8.0	$C_{17} H_{20} O_1$
241.1595	34.3282	241.1592	1.0	0.2	7.5	$C_{17} H_{21} O_1$
242.1613	8.0068	242.1671	-23.8	-5.8	7.0	$C_{17} H_{22} O_1$
242.9856	10.7517					
243.1680	2.6426					
244.1800	1.3706	244.1827	-11.1	-2.7	6.0	$C_{17} H_{24} O_1$
249.1641	1.2879	249.1643	-0.9	-0.2	9.5	$C_{19} H_{21}$
251.1567	2.5020					
251.1719	2.5436					
253.1576	9.4321	253.1592	-6.6	-1.7	8.5	$C_{18} H_{21} O_1$
254.1609	2.9978					
254.9856	3.1008					
255.1765	4.7361	255.1749	6.1	1.6	7.5	$C_{18} H_{23} O_1$
256.1792	5.3710	256.1827	-13.6	-3.5	7.0	$C_{18} H_{24} O_1$
257.1801	1.5230					
259.1601	1.4768					
265.1876	3.7302					
266.1636	1.9155	266.1671	-13.0	-3.4	9.0	$C_{19} H_{22} O_1$
266.1934	1.7699	266.1882	19.6	5.2	4.0	$C_{16} H_{26} O_3$
266.9826	2.2321					
267.1765	5.1427	267.1749	6.0	1.6	8.5	$C_{19} H_{23} O_1$
268.1781	3.8653	268.1827	-17.3	-4.6	8.0	$C_{19} H_{24} O_1$
268.9824	3.2185					
269.1880	6.3470	269.1905	-9.5	-2.5	7.5	$C_{19} H_{25} O_1$
270.1949	1.4897	270.1984	-12.8	-3.5	7.0	$C_{19} H_{26} O_1$
274.1938	3.5868	274.1933	2.0	0.6	6.0	$C_{18} H_{26} O_2$
275.1837	1.5065	275.1800	13.5	3.7	10.5	$C_{21} H_{23}$
279.1820	1.5170					
280.9824	7.4444					
281.1917	3.9565	281.1905	4.1	1.2	8.5	$C_{20} H_{25} O_1$
282.1968	2.3667	282.1984	-5.7	-1.6	8.0	$C_{20} H_{26} O_1$
283.2082	10.3496	283.2062	7.0	2.0	7.5	$C_{20} H_{27} O_1$
284.2158	6.7966	284.2140	6.2	1.8	7.0	$C_{20} H_{28} O_1$
285.2192	1.5294	285.2218	-9.2	-2.6	6.5	$C_{20} H_{29} O_1$
287.2091	1.0827					
291.1752	1.2563	291.1749	1.1	0.3	10.5	$C_{21} H_{23} O_1$
292.9824	11.0504					
293.1918	7.8888	293.1905	4.3	1.3	9.5	$C_{21} H_{25} O_1$
294.1981	1.8412	294.1984	-0.7	-0.2	9.0	$C_{21} H_{26} O_1$
295.1985	1.0957					
297.1860	1.0710	297.1855	2.0	0.6	8.5	$C_{20} H_{25} O_2$
298.1901	2.3438	298.1933	-10.6	-3.2	8.0	$C_{20} H_{26} O_2$
298.2272	2.3886	298.2297	-8.1	-2.4	7.0	$C_{21} H_{30} O_1$
299.2031	3.4644	299.2011	6.7	2.0	7.5	$C_{20} H_{27} O_2$
300.2091	1.5522	300.2089	0.4	0.1	7.0	$C_{20} H_{28} O_2$
301.2118	1.4331	301.2168	-16.5	-5.0	6.5	$C_{20} H_{29} O_2$
304.9824	2.3586					
307.1707	1.0914	307.1698	2.9	0.9	10.5	$C_{21} H_{23} O_2$
308.2144	4.8190	308.2140	1.1	0.3	9.0	$C_{22} H_{28} O_1$
309.1909	7.9338	309.1855	17.6	5.4	9.5	$C_{21} H_{25} O_2$
310.1917	2.6948	310.1933	-5.2	-1.6	9.0	$C_{21} H_{26} O_2$
311.1993	45.8148	311.2011	-5.7	-1.8	8.5	$C_{21} H_{27} O_2$
312.2023	11.1425					
313.2109	1.4069	313.2168	-18.8	-5.9	7.5	$C_{21} H_{29} O_2$
316.9794	1.3110					
324.2056	7.1655	324.2089	-10.3	-3.3	9.0	$C_{22} H_{28} O_2$
325.2178	1.7057	325.2168	3.3	1.1	8.5	$C_{22} H_{29} O_2$
326.2232	44.0701	326.2246	-4.2	-1.4	8.0	$C_{22} H_{30} O_2$
327.2266	11.4459	327.2324	-17.8	-5.8	7.5	$C_{22} H_{31} O_2$
328.2291	2.4050					
330.9792	3.5883					
342.2260	1.1861					
342.9792	4.4157					
344.2348	4.3784	344.2351	-1.0	-0.4	7.0	$C_{22} H_{32} O_3$
345.2387	1.3757	345.2430	-12.3	-4.2	6.5	$C_{22} H_{33} O_3$
354.9792	1.6937					

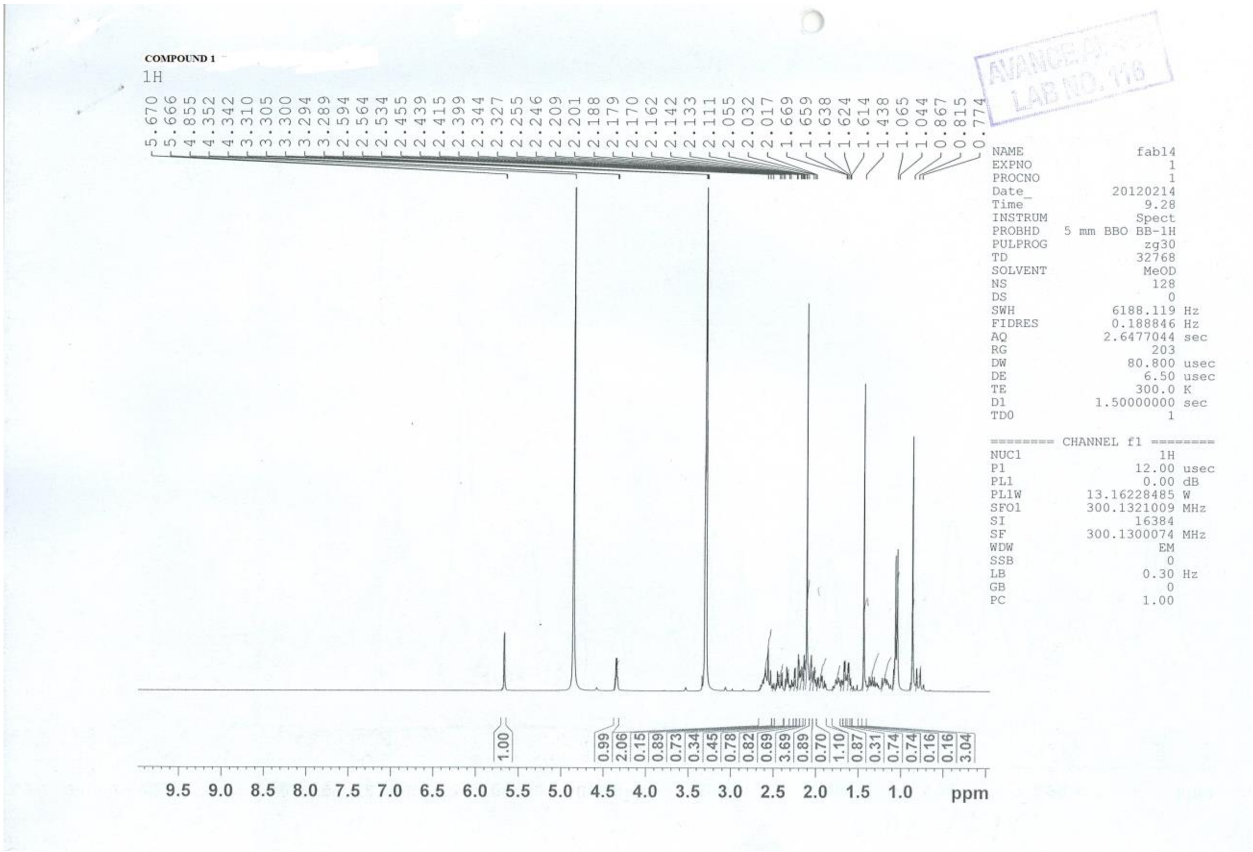
COMPOUND 1
1H
5.670
5.666
4.855
4.352
4.342
3.310
3.305
3.300
3.294
3.289
2.594
2.564
2.534
2.455
2.439
2.415
2.399
2.344
2.327
2.255
2.246
2.209
2.201
2.188
2.179
2.170
2.162
2.142
2.133
2.111
2.055
2.032
2.017
1.669
1.659
1.638
1.624
1.614
1.438
1.065
1.044
0.867
0.815
0.774
NAME fab14
EXPNO 1
PROCNO 1
Date_ 20120214
Time 9.28
INSTRUM Spect
PROBHD 5 mm BBO BB-1H
PULPROG zg30
TD 32768
SOLVENT MeOD
NS 128
DS 0
SWH 6188.119 Hz
FIDRES 0.188846 Hz
AQ 2.6477044 sec
RG 203
DW 80.800 usec
DE 6.50 usec
TE 300.0 K
D1 1.50000000 sec
TD0 1
======== CHANNEL f1 ========
NUC1 1H
P1 12.00 usec
PL1 0.00 dB
PL1W 13.16228485 W
SFO1 300.1321009 MHz
SI 16384
SF 300.1300074 MHz
WDW EM
SSB 0
LB 0.30 Hz
GB 0
PC 1.00
1.00
0.99
2.06
0.15
0.89
0.73
0.34
0.45
0.78
0.82
0.69
3.69
0.89
0.70
1.10
0.87
0.31
0.74
0.74
0.16
0.16
3.04
9.5 9.0 8.5 8.0 7.5 7.0 6.5 6.0 5.5 5.0 4.5 4.0 3.5 3.0 2.5 2.0 1.5 1.0 ppm

COMPOUND 1
BB

AVANCE AV-500
CRYOPROBE
LAB NO: 108

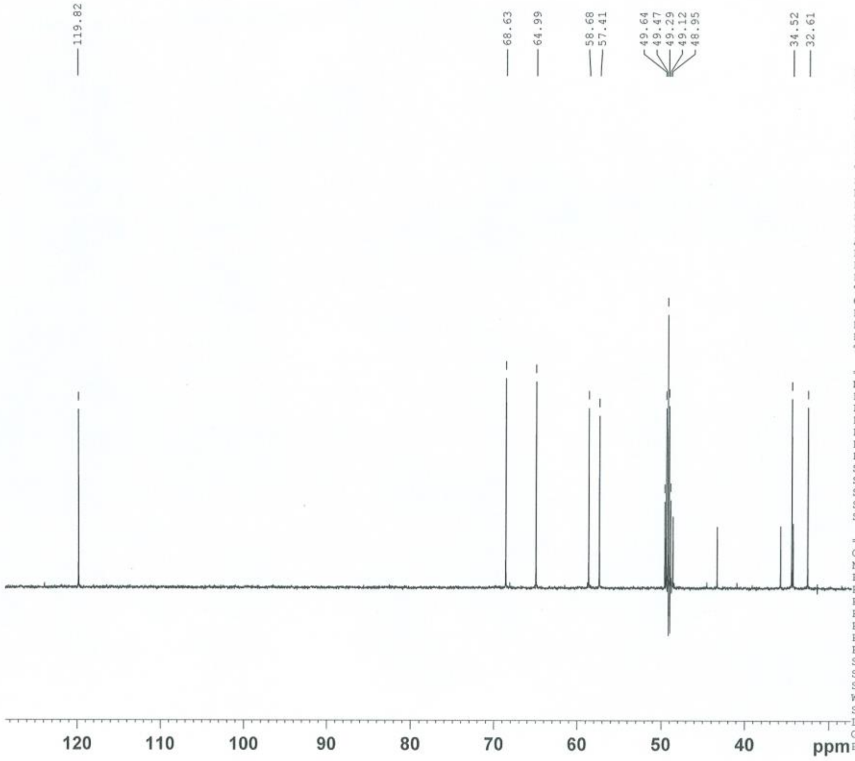

NAME	mar09-12
EXPNO	9
PROCNO	1
Date_	20120310
Time	8.40
INSTRUM	spect
PROBHD	5 mm CPDUL 13C
PULPROG	deptsp90
TD	32768
SOLVENT	MeOD
NS	1153
DS	2
SWH	25252.525 Hz
FIDRES	0.770646 Hz
AQ	0.6488762 sec
RG	32768
DW	19.800 usec
DE	6.50 usec
TE	293.5 K
CNST2	145.0000000
D1	1.50000000 sec
D2	0.00344828 sec
D12	0.00002000 sec
TD0	4
======== CHANNEL f1 ========	
NUC1	13C
P1	7.80 usec
P12	2000.00 usec
PL0	120.00 dB
PL1	3.00 dB
PL0W	0.00000000 W
PL1W	33.36152267 W
SFO1	125.8206598 MHz
SP2	1.99 dB
SPNAM2	Crp60comp.4
SPOAL2	0.500
SPOFFS2	0.00 Hz
======== CHANNEL f2 ========	
CPDPRG2	waltz16
NUC2	1H
P3	13.50 usec
P4	27.00 usec
PCPD2	80.00 usec
PL2	2.00 dB
PL12	18.50 dB
PL2W	11.25274181 W
PL12W	0.25191751 W
SFO2	500.3330020 MHz
SI	32768
SF	125.8079004 MHz
WDW	EM
SSB	0
LB	1.00 Hz
GB	0
PC	2.00

COMPOUND 1
DEPT-135

AVANCE AV-500
CRYOPROBE
LAB NO: 108

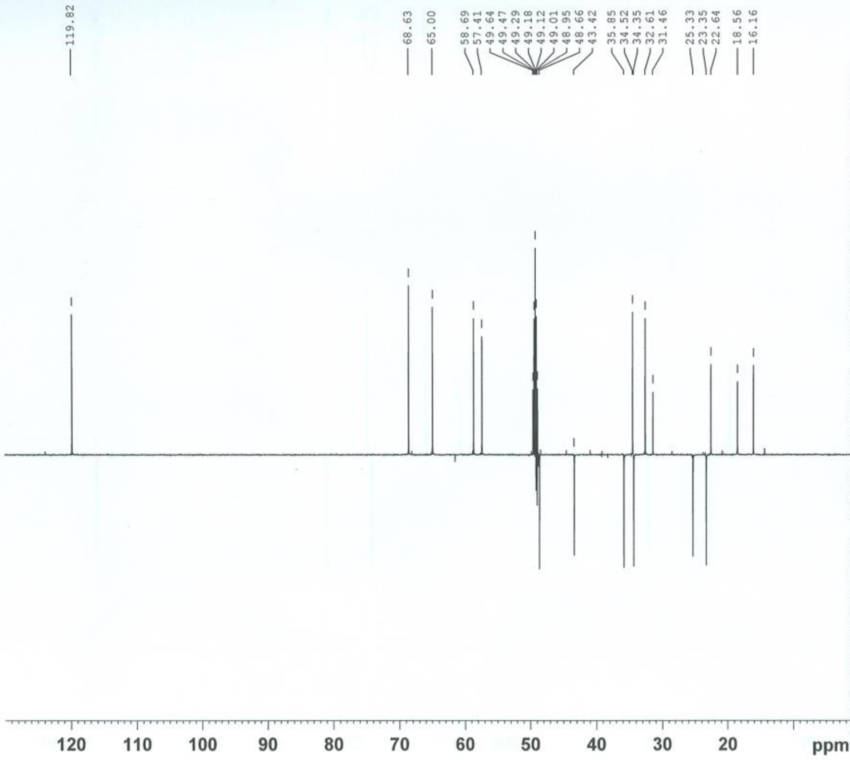

NAME	mar09-12
EXPNO	8
PROCNO	1
Date_	20120310
Time	6.12
INSTRUM	spect
PROBHD	5 mm CPDUL 13C
PULPROG	deptsp135
TD	32768
SOLVENT	MeOD
NS	4506
DS	2
SWH	25252.525 Hz
FIDRES	0.770646 Hz
AQ	0.6488762 sec
RG	32768
DW	19.800 usec
DE	6.50 usec
TE	294.2 K
CNST2	145.0000000
D1	1.50000000 sec
D2	0.00344828 sec
D12	0.00002000 sec
TD0	7
======== CHANNEL f1 ========	
NUC1	13C
P1	7.80 usec
P12	2000.00 usec
PL0	120.00 dB
PL1	3.00 dB
PL0W	0.00000000 W
PL1W	33.36152267 W
SFO1	125.8206598 MHz
SP2	1.99 dB
SPNAM2	Crp60comp.4
SPOAL2	0.500
SPOFFS2	0.00 Hz
======== CHANNEL f2 ========	
CPDPRG2	waltz16
NUC2	1H
P3	13.50 usec
P4	27.00 usec
PCPD2	80.00 usec
PL2	2.00 dB
PL12	18.50 dB
PL2W	11.25274181 W
PL12W	0.25191751 W
SFO2	500.3330020 MHz
SI	32768
SF	125.8079004 MHz
WDW	EM
SSB	0
LB	1.00 Hz
GB	0
PC	2.00

COMPOUND 1
DEPT-90

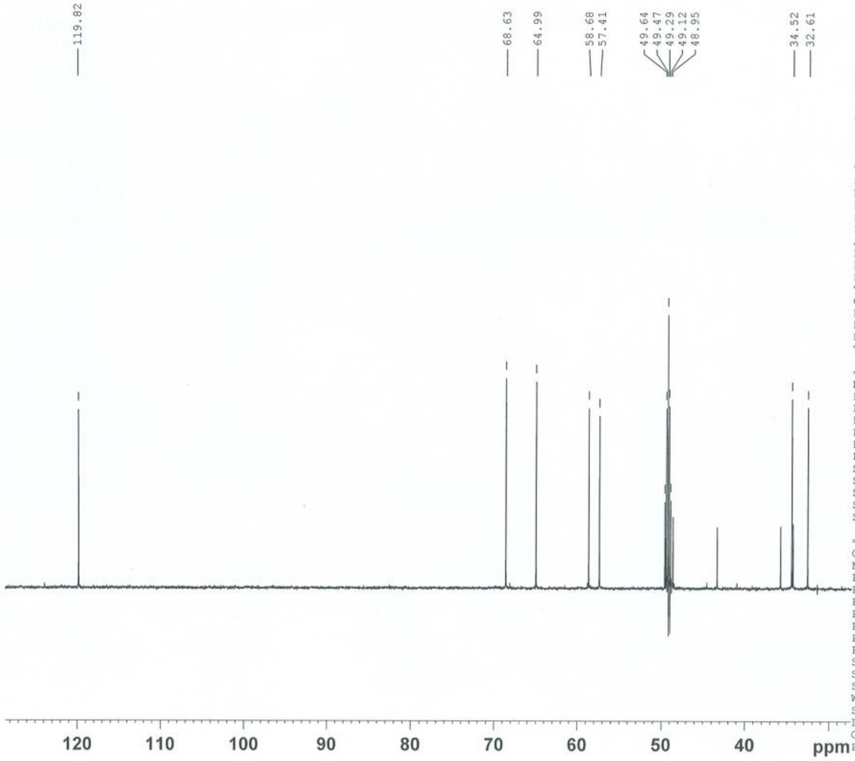

AVANCE AV-500

NAME	mar09-12	
EXPNO	9	
PROCNO	1	
Date_	20120310	
Time	8.40	
INSTRUM	spect	
PROBHD	5 mm CPDUL 13C	
PULPROG	deptsp90	
TD	32768	
SOLVENT	MeOD	
NS	1153	
DS	2	
SWH	25252.525	Hz
FIDRES	0.770646	Hz
AQ	0.6488762	sec
RG	32768	
DW	19.800	usec
DE	6.50	usec
TE	293.5	K
CNST2	145.0000000	
D1	1.50000000	sec
D2	0.00344828	sec
D12	0.00002000	sec
TD0	4	
======== CHANNEL f1 ========		
NUC1	13C	
P1	7.80	usec
P12	2000.00	usec
PL0	120.00	dB
PL1	3.00	dB
PL0W	0.00000000	W
PL1W	33.36152267	W
SFO1	125.8206598	MHz
SP2	1.99	dB
SPNAM2	Crp60comp.4	
SPOAL2	0.500	
SPOFFS2	0.00	Hz
======== CHANNEL f2 ========		
CPDPRG2	waltz16	
NUC2	1H	
P3	13.50	usec
P4	27.00	usec
PCPD2	80.00	usec
PL2	2.00	dB
PL12	18.50	dB
PL2W	11.25274181	W
PL12W	0.25191751	W
SFO2	500.3330020	MHz
SI	32768	
SF	125.8079004	MHz
WDW	EM	
SSB	0	
LB	1.00	Hz
GB	0	
PC	2.00	

COMPOUND 1
HSQC

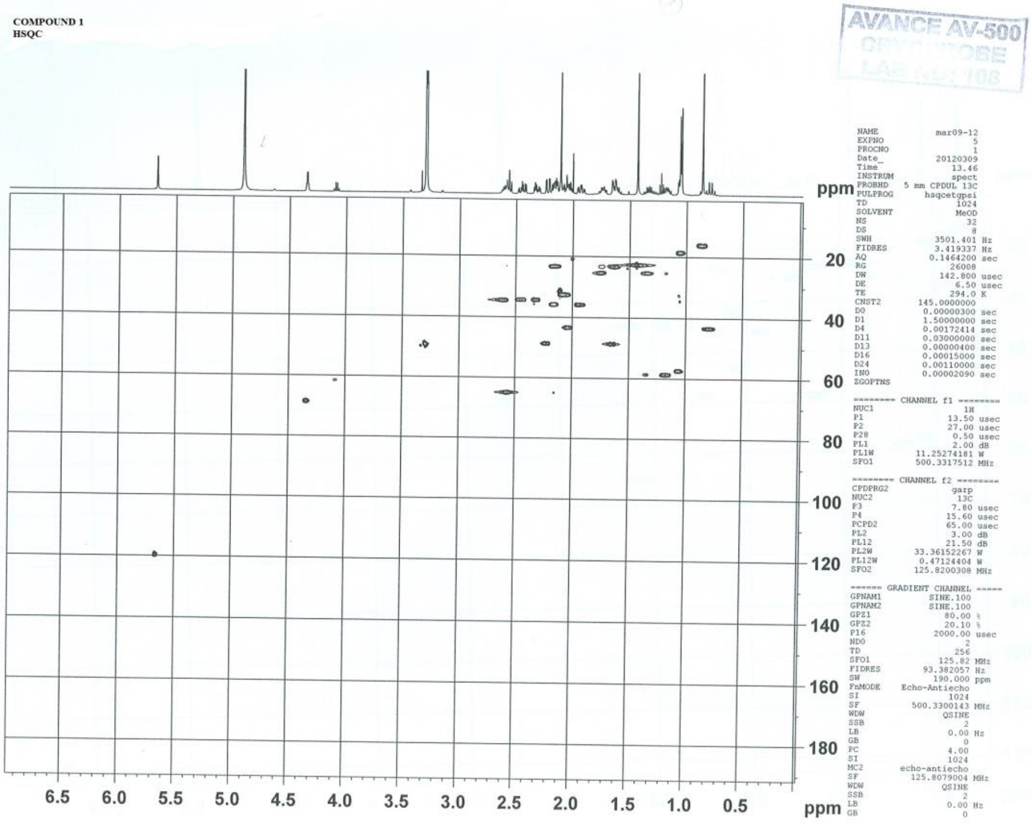

COMPOUND 1
COSY

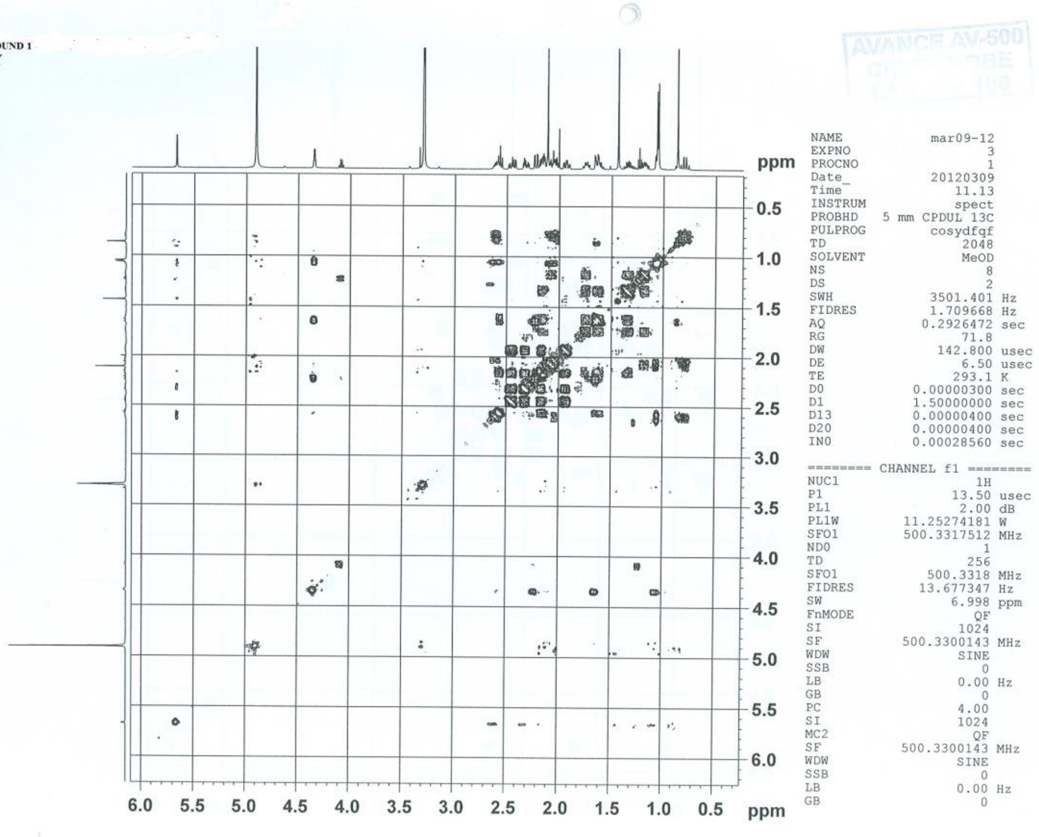

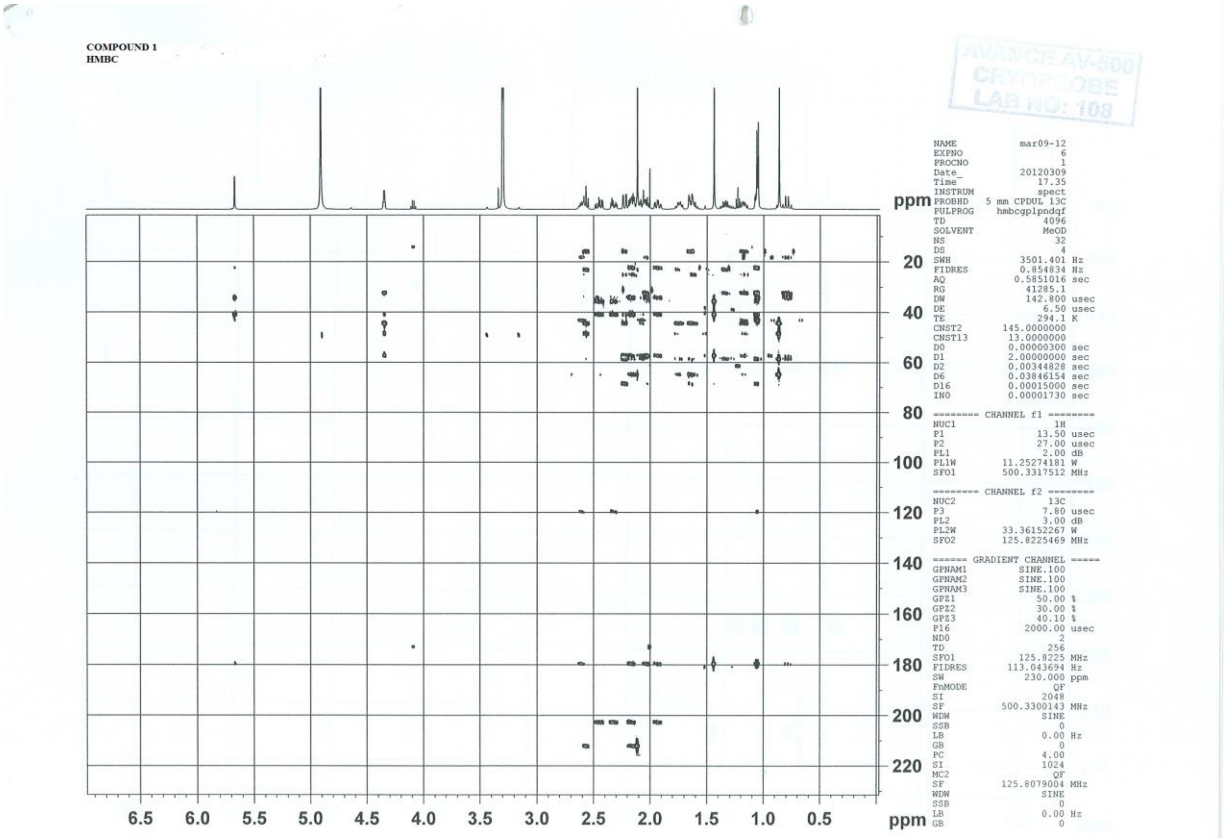
COMPOUND 1
HMBC
AVANCE AV-500
CRYOPROBE
LAB NO: 108
NAME mar09-12
EXPNO 6
PROCNO 1
Date_ 20120309
Time 17.35
INSTRUM spect
PROBHD 5 mm CPDUL 13C
PULPROG hmbcgplpndqf
TD 4096
SOLVENT MeOD
NS 32
DS 4
SWH 3501.401 Hz
FIDRES 0.854834 Hz
AQ 0.5851016 sec
RG 41285.1
DW 142.800 usec
DE 6.50 usec
TE 294.1 K
CNST2 145.0000000
CNST13 13.0000000
D0 0.00000300 sec
D1 2.00000000 sec
D2 0.00344828 sec
D6 0.03846154 sec
D16 0.00015000 sec
IN0 0.00001730 sec
======== CHANNEL f1 ========
NUC1 1H
P1 13.50 usec
P2 27.00 usec
PL1 2.00 dB
PL1W 11.25274181 W
SFO1 500.3317512 MHz
======== CHANNEL f2 ========
NUC2 13C
P3 7.80 usec
PL2 3.00 dB
PL2W 33.36152267 W
SFO2 125.8225469 MHz
====== GRADIENT CHANNEL =====
GPNAM1 SINE.100
GPNAM2 SINE.100
GPNAM3 SINE.100
GPZ1 50.00 %
GPZ2 30.00 %
GPZ3 40.10 %
P16 2000.00 usec
ND0 2
TD 256
SFO1 125.8225 MHz
FIDRES 113.043694 Hz
SW 230.000 ppm
FnMODE QF
SI 2048
SF 500.3300143 MHz
WDW SINE
SSB 0
LB 0.00 Hz
GB 0
PC 4.00
SI 1024
MC2 QF
SF 125.8079004 MHz
WDW SINE
SSB 0
LB 0.00 Hz
GB 0
ppm
20
40
60
80
100
120
140
160
180
200
220
6.5
6.0
5.5
5.0
4.5
4.0
3.5
3.0
2.5
2.0
1.5
1.0
0.5
ppm

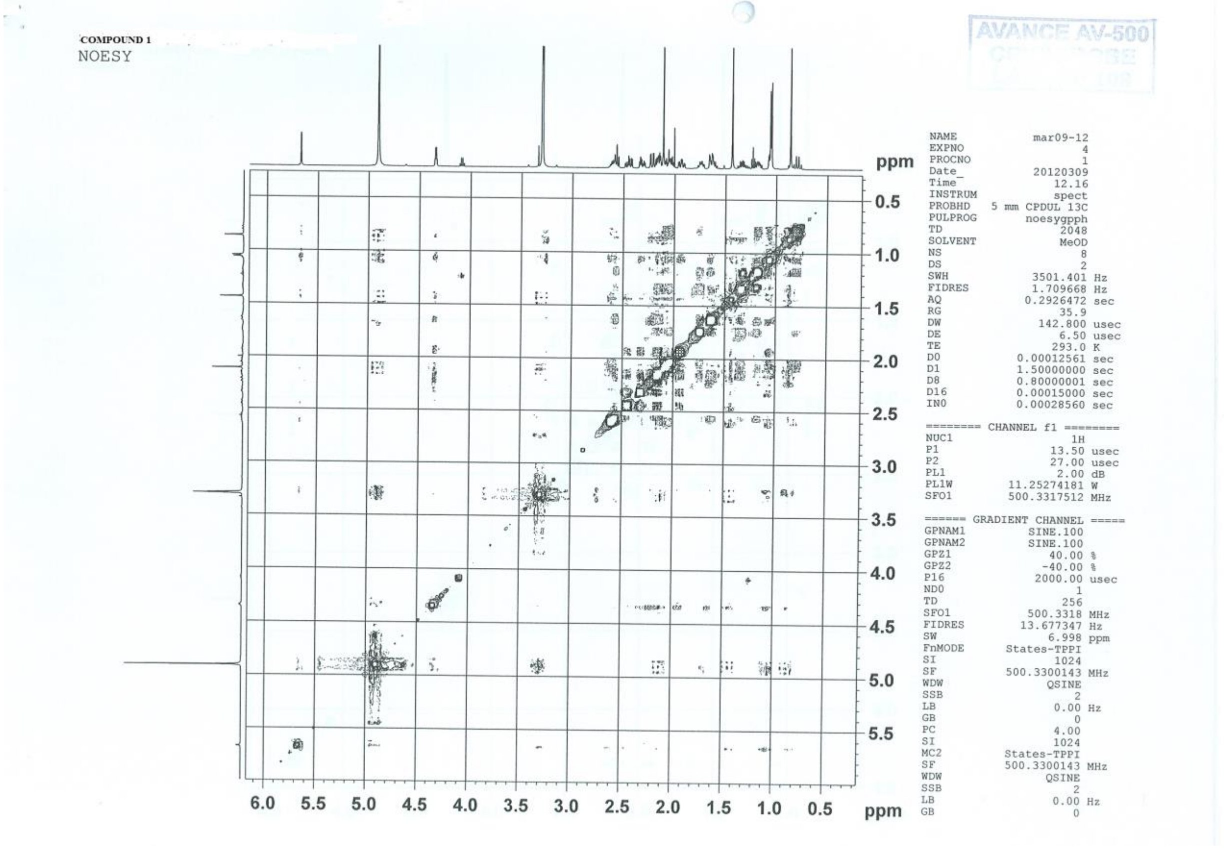
COMPOUND 1
NOESY
AVANCE AV-500
NAME mar09-12
EXPNO 4
PROCNO 1
Date_ 20120309
Time 12.16
INSTRUM spect
PROBHD 5 mm CPDUL 13C
PULPROG noesygpph
TD 2048
SOLVENT MeOD
NS 8
DS 2
SWH 3501.401 Hz
FIDRES 1.709668 Hz
AQ 0.2926472 sec
RG 35.9
DW 142.800 usec
DE 6.50 usec
TE 293.0 K
D0 0.00012561 sec
D1 1.50000000 sec
D8 0.80000001 sec
D16 0.00015000 sec
IN0 0.00028560 sec
======== CHANNEL f1 ========
NUC1 1H
P1 13.50 usec
P2 27.00 usec
PL1 2.00 dB
PL1W 11.25274181 W
SFO1 500.3317512 MHz
====== GRADIENT CHANNEL =====
GPNAM1 SINE.100
GPNAM2 SINE.100
GPZ1 40.00 %
GPZ2 -40.00 %
P16 2000.00 usec
ND0 1
TD 256
SFO1 500.3318 MHz
FIDRES 13.677347 Hz
SW 6.998 ppm
FnMODE States-TPPI
SI 1024
SF 500.3300143 MHz
WDW QSINE
SSB 2
LB 0.00 Hz
GB 0
PC 4.00
SI 1024
MC2 States-TPPI
SF 500.3300143 MHz
WDW QSINE
SSB 2
LB 0.00 Hz
GB 0
ppm
0.5
1.0
1.5
2.0
2.5
3.0
3.5
4.0
4.5
5.0
5.5
6.0
5.5
5.0
4.5
4.0
3.5
3.0
2.5
2.0
1.5
1.0
0.5
ppm
