# Supplementary material for: New Anti-Inflammatory Metabolites by Microbial Transformation of Medrysone
Source: PLoS One. 2016 Apr 22;11(4):e0153951. doi: 10.1371/journal.pone.0153951 (PMC4841542; doi:10.1371/journal.pone.0153951)
Supplement: S2 File — (PDF) [file pone.0153951.s002.pdf]

Date Run: 11-10-2012 (Time Run: 09:52:18)

COMPOUND 2
Instrument: JEOL MSRoute
Inlet: My Inlet **Ionization mode: EI+**

Scan: 7 R.T.: .53
Base: m/z 177; 54.4%FS TIC: 13337782 #Ions: 310

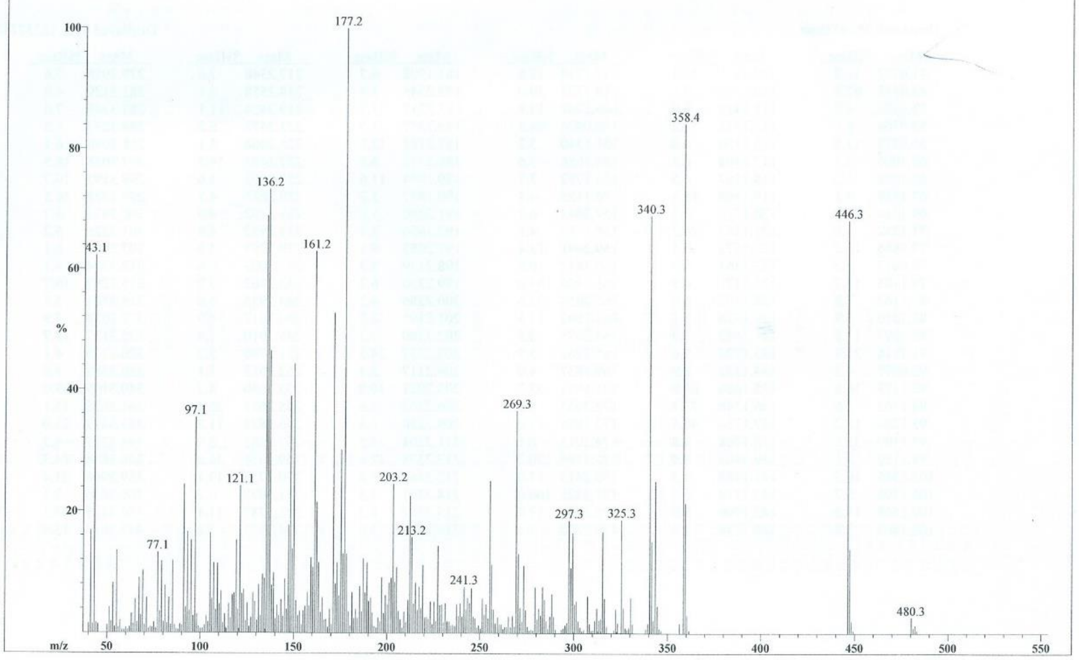

COMPOUND-2
HREI-MS

Mass	Relative Intensity	Theoretical Mass	Delta [mmu]	RDB	Composition
255.1710	2.8	255.1743	-3.3	7.5	$C_{18} H_{23} O_{1}$
255.2250	3.3				
256.1263	1.6	256.1247	1.6	13.0	$C_{20} H_{16}$
256.1642	1.5	256.1669	-2.8	3.0	$C_{14} H_{24} O_{4}$
256.2220	10.2	256.2186	3.4	6.0	$C_{19} H_{28}$
257.2119	3.4	257.2111	0.8	1.5	$C_{15} H_{29} O_{3}$
260.1710	1.2				
262.1231	2.2	262.1200	3.1	7.0	$C_{15} H_{18} O_{4}$
264.0250	1.1	264.0206	4.4	18.0	$C_{19} H_{4} O_{2}$
264.1125	5.4	264.1145	-2.0	11.0	$C_{18} H_{16} O_{2}$
265.1064	2.0	265.1012	5.3	15.5	$C_{21} H_{13}$
267.1076	1.3				
268.9824	4.7	268.9869	-4.5	17.5	$C_{17} H_{1} O_{4}$
270.0010	2.8				
271.0074	1.2	271.0026	4.8	16.5	$C_{17} H_{3} O_{4}$
273.0327	1.6	273.0335	-0.8	19.5	$C_{21} H_{5} O_{1}$
279.1469	2.5				
280.9824	2.9	280.9869	-4.5	18.5	$C_{18} H_{1} O_{4}$
281.1646	1.5				
282.1629	1.5	282.1614	1.5	9.0	$C_{19} H_{22} O_{2}$
283.1731	1.7	283.1693	3.9	8.5	$C_{19} H_{23} O_{2}$
284.2822	6.1				
285.2877	2.4				
292.9824	7.0	292.9869	-4.5	19.5	$C_{19} H_{1} O_{4}$
297.1860	3.7	297.1849	1.1	8.5	$C_{20} H_{25} O_{2}$
297.2738	2.0	297.2788	-5.0	1.5	$C_{19} H_{37} O_{2}$
298.1955	2.5	298.1927	2.8	8.0	$C_{20} H_{26} O_{2}$
299.1965	1.6	299.2006	-4.1	7.5	$C_{20} H_{27} O_{2}$
304.9824	1.3	304.9869	-4.5	20.5	$C_{20} H_{1} O_{4}$
307.1650	2.2	307.1693	-4.3	10.5	$C_{21} H_{23} O_{2}$
311.2221	1.5	311.2217	0.4	3.5	$C_{18} H_{31} O_{4}$
312.2060	1.2	312.2084	-2.4	8.0	$C_{21} H_{28} O_{2}$
313.2715	3.7	313.2737	-2.2	1.5	$C_{19} H_{37} O_{3}$
315.1980	1.0	315.1955	2.5	7.5	$C_{20} H_{27} O_{3}$
322.1974	1.1	322.1927	4.7	10.0	$C_{22} H_{26} O_{2}$
325.1805	3.9	325.1798	0.7	9.5	$C_{21} H_{25} O_{3}$
330.9792	2.0				
339.2643	2.0	339.2682	-3.9	7.5	$C_{24} H_{35} O_{1}$
340.2055	11.6	340.2033	2.2	9.0	$C_{22} H_{28} O_{3}$
341.2092	3.0	341.2111	-1.9	8.5	$C_{22} H_{29} O_{3}$
342.9792	2.3				
343.1983	2.0				
356.1931	1.1	356.1982	-5.1	9.0	$C_{22} H_{28} O_{4}$
358.2126	4.8	358.2139	-1.3	8.0	$C_{22} H_{30} O_{4}$
367.2746	3.1				
368.3366	3.1				
369.3414	1.1	369.3363	5.1	1.5	$C_{23} H_{45} O_{3}$
380.9760	1.1				
392.9760	2.5				
393.2989	1.3	393.2999	-1.0	4.5	$C_{24} H_{41} O_{4}$
423.3491	1.2	423.3469	2.2	3.5	$C_{26} H_{47} O_{4}$
430.9729	1.0				
551.5206	2.7	551.5186	2.0	6.5	$C_{39} H_{67} O_{1}$
576.5173	1.4				
577.5380	2.0	577.5343	3.7	7.5	$C_{41} H_{69} O_{1}$

COMPOUND 2
1H-NMR

AVANCE AV - III
300 MHz, LAB # 116

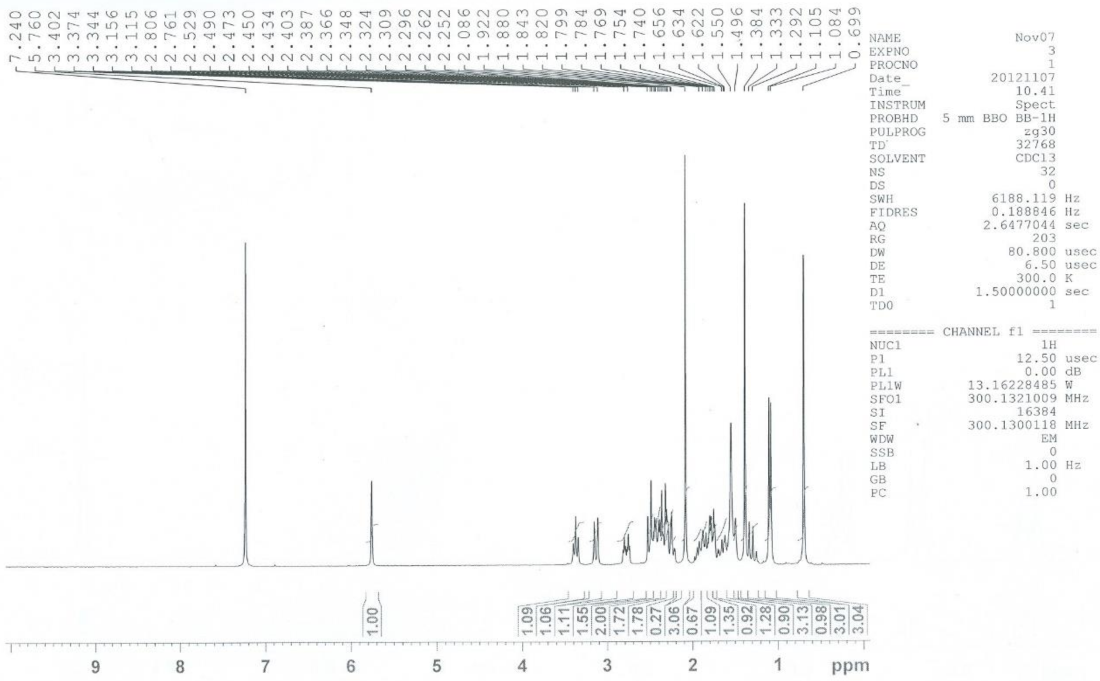

NAME	Nov07
EXPNO	3
PROCNO	1
Date_	20121107
Time	10.41
INSTRUM	Spect
PROBHD	5 mm BBO BB-1H
PULPROG	zg30
TD	32768
SOLVENT	CDCl3
NS	32
DS	0
SWH	6188.119 Hz
FIDRES	0.188846 Hz
AQ	2.6477044 sec
RG	203
DW	80.800 usec
DE	6.50 usec
TE	300.0 K
D1	1.50000000 sec
TD0	1

======== CHANNEL f1 ========

NUC1	1H
P1	12.50 usec
PL1	0.00 dB
PL1W	13.16228485 W
SFO1	300.1321009 MHz
SI	16384
SF	300.1300118 MHz
WDW	EM
SSB	0
LB	1.00 Hz
GB	0
PC	1.00

H.E.J. Research Institute of Chemistry.

COMPOUND 2

BB

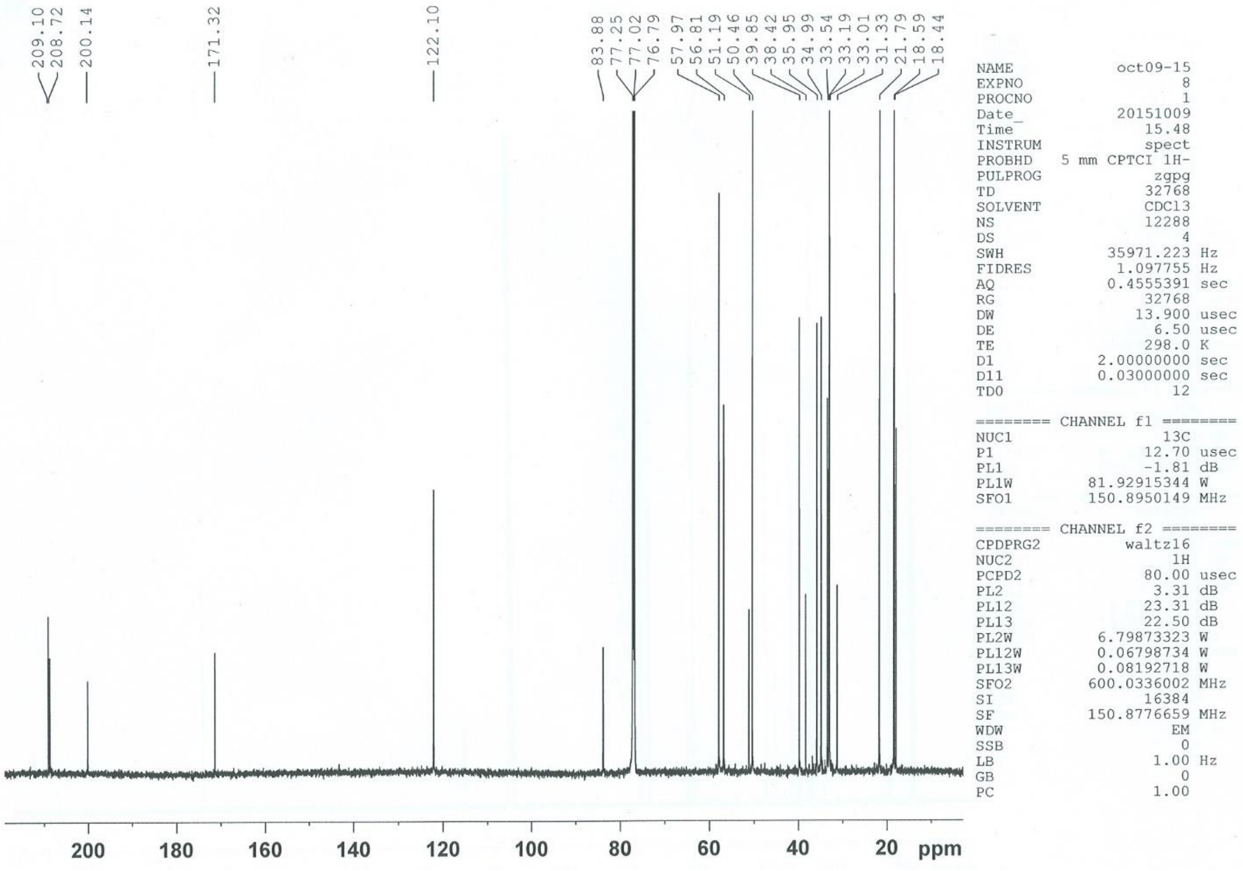

COMPOUND 2

dept135

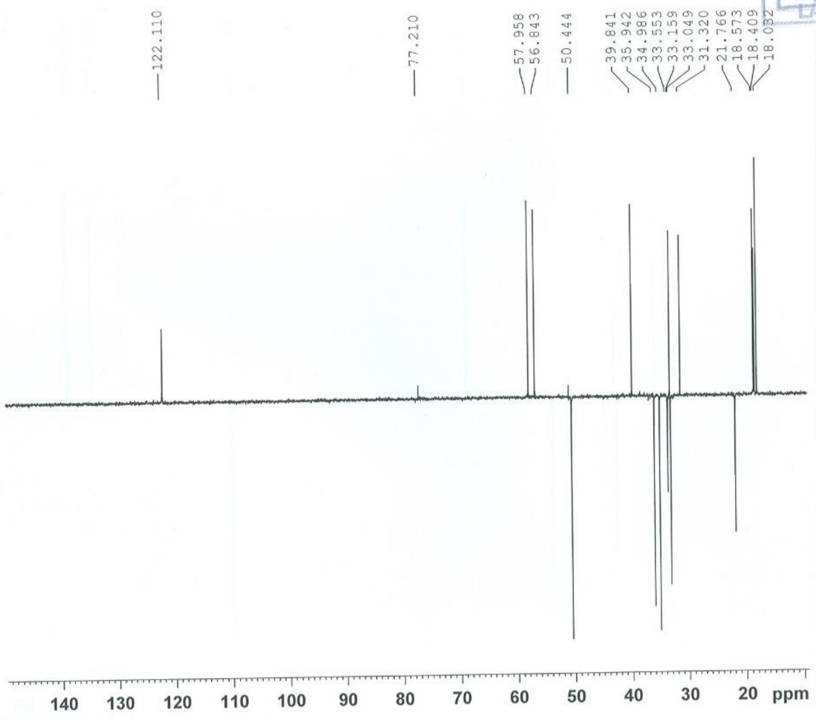


```
NAME          oct09-15
EXPNO                9
PROCNO               1
Date_         20151010
Time              0.17
INSTRUM          spect
PROBHD   5 mm CPTCI 1H-
PULPROG     deptsp135
TD               32768
SOLVENT          CDC13
NS                6144
DS                   2
SWH          30303.031 Hz
FIDRES        0.924775 Hz
AQ           0.5407385 sec
RG               32768
DW              16.500 usec
DE                6.50 usec
TE               298.0 K
CNST2      145.0000000
D1          1.50000000 sec
D2          0.00344828 sec
D12         0.00002000 sec
TD0                  6

======== CHANNEL f1 ========
NUC1               13C
P1               12.70 usec
P12            2000.00 usec
PL0             120.00 dB
PL1              -1.81 dB
PL0W        0.00000000 W
PL1W       81.92915344 W
SFO1       150.8927518 MHz
SP2               4.19 dB
SPNAM2     Crp60comp.4
SPOAL2           0.500
SPOFFS2           0.00 Hz

======== CHANNEL f2 ========
CPDPRG2        waltz16
NUC2                1H
P3                8.00 usec
P4               16.00 usec
PCPD2            80.00 usec
PL2               3.31 dB
PL12             23.31 dB
PL2W        6.79873323 W
PL12W       0.06798734 W
SFO2       600.0330002 MHz
SI               16384
SF         150.8776659 MHz
WDW                 EM
SSB                  0
LB                1.00 Hz
GB                   0
PC                1.40
```

COMPOUND 2

dept 90

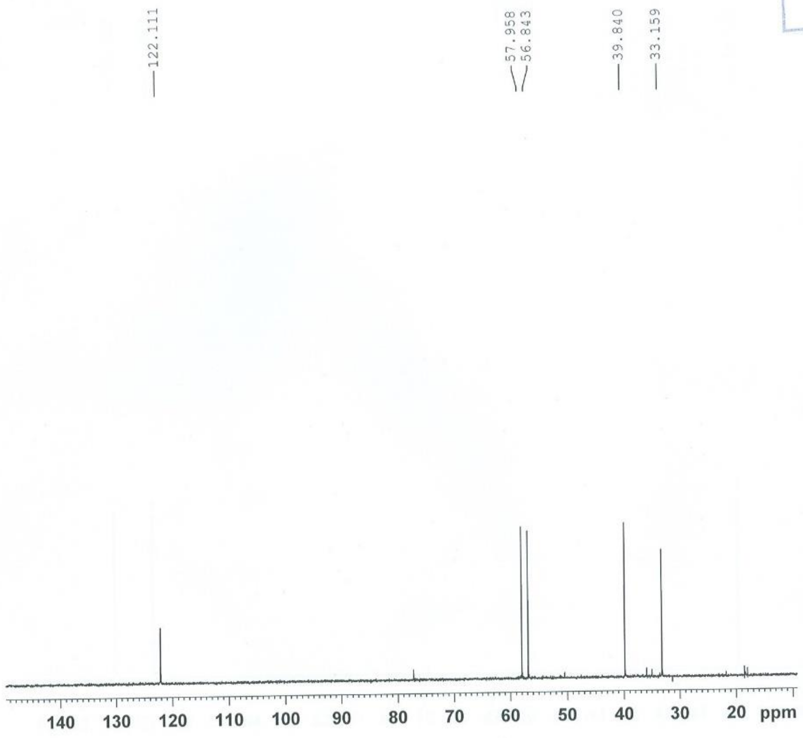

AVANCE AV-600-LC
CRYOPROBE
LAB NO: 108

NAME	oct09-15
EXPNO	10
PROCNO	1
Date_	20151010
Time	3.51
INSTRUM	spect
PROBHD	5 mm CPTCI 1H-
PULPROG	deptsp90
TD	32768
SOLVENT	CDCl3
NS	3072
DS	2
SWH	30303.031 Hz
FIDRES	0.924775 Hz
AQ	0.5407385 sec
RG	32768
DW	16.500 usec
DE	6.50 usec
TE	298.0 K
CNST2	145.0000000
D1	1.50000000 sec
D2	0.00344828 sec
D12	0.00002000 sec
TD0	3

======== CHANNEL f1 ========

NUC1	13C
P1	12.70 usec
P12	2000.00 usec
PL0	120.00 dB
PL1	-1.81 dB
PL0W	0.00000000 W
PL1W	81.92915344 W
SFO1	150.8927518 MHz
SP2	4.19 dB
SPNAM2	Crp60comp.4
SPOAL2	0.500
SPOFFS2	0.00 Hz

======== CHANNEL f2 ========

CPDPRG2	waltz16
NUC2	1H
P3	8.00 usec
P4	16.00 usec
PCPD2	80.00 usec
PL2	3.31 dB
PL12	23.31 dB
PL2W	6.79873323 W
PL12W	0.06798734 W
SFO2	600.0330002 MHz
SI	16384
SF	150.8776659 MHz
WDW	EM
SSB	0
LB	1.00 Hz
GB	0
PC	1.40

COMPOUND-2
HSQC

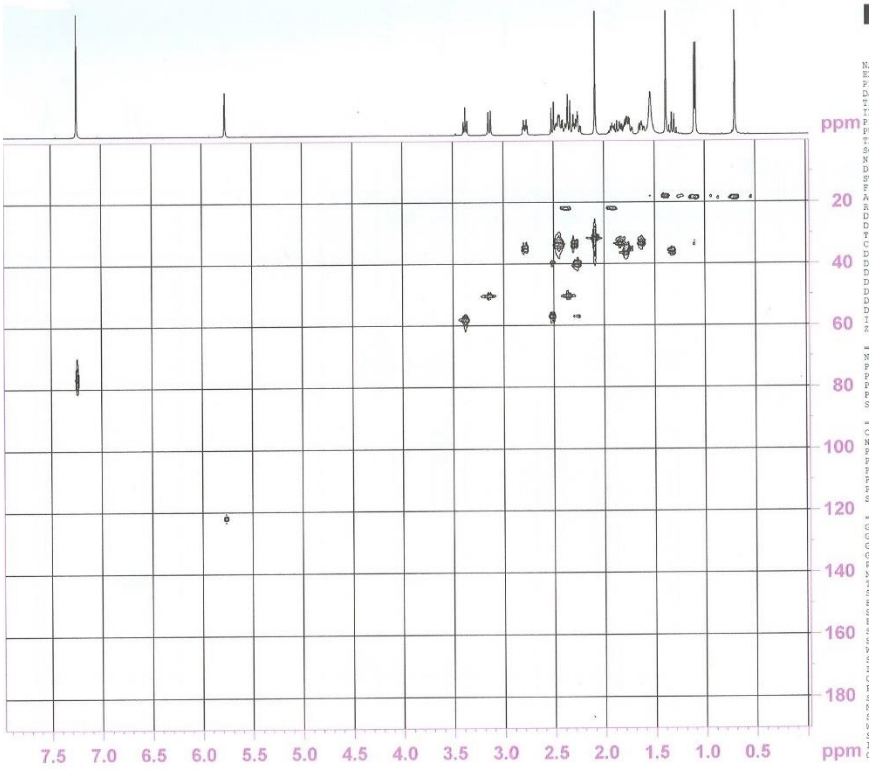

BRUKER

NAME	nov08-12
EXPNO	5
PROCNO	1
Date_	20121108
Time	20.31
INSTRUM	spect
PROBHD	5 mm PABBI 1H/
PULPROG	hsqcetgpsi
TD	1024
SOLVENT	CDCl3
NS	32
DS	8
SWH	4006.410 Hz
FIDRES	3.912510 Hz
AQ	0.1279700 sec
RG	20642.5
DW	124.800 usec
DE	6.50 usec
TE	297.5 K
CNST2	145.0000000
D0	0.00000300 sec
D1	2.00000000 sec
D4	0.00172414 sec
D11	0.03000000 sec
D13	0.00000400 sec
D16	0.00020000 sec
D24	0.00110000 sec
IN0	0.00002090 sec
ZGOPTNS	

======== CHANNEL f1 ========

NUC1	1H
P1	7.05 usec
P2	14.10 usec
P28	1000.00 usec
PL1	5.00 dB
SFO1	500.2320009 MHz

======== CHANNEL f2 ========

CPDPRG2	garp
NUC2	13C
P3	15.25 usec
P4	30.50 usec
PCPD2	70.00 usec
PL2	-3.00 dB
PL12	9.00 dB
SFO2	125.7948834 MHz

====== GRADIENT CHANNEL =====

GPNAM1	SINE.100
GPNAM2	SINE.100
GPZ1	80.00 %
GPZ2	20.10 %
P16	1000.00 usec
ND0	2
TD	256
SFO1	125.7949 MHz
FIDRES	93.363388 Hz
SW	190.000 ppm
FnMODE	Echo-Antiecho
SI	1024
SF	500.2300193 MHz
WDW	QSINE
SSB	2
LB	0.00 Hz
GB	0
PC	1.00
SI	1024
MC2	echo-antiecho
SF	125.7829370 MHz
WDW	QSINE
SSB	2
LB	0.00 Hz
GB	0

COMPOUND 2
COSY

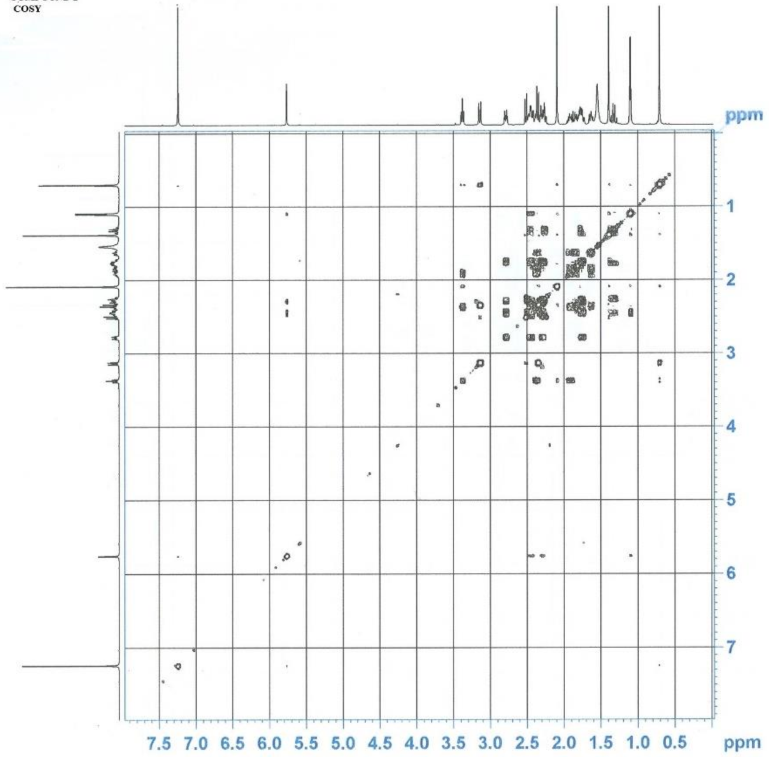

BRUKER

NAME	nov08-12	
EXPNO	3	
PROCNO	1	
Date_	20121108	
Time	16.32	
INSTRUM	spect	
PROBHD	5 mm PABBI 1H/	
PULPROG	cosygpqf	
TD	2048	
SOLVENT	CDCl3	
NS	8	
DS	8	
SWH	4006.410	Hz
FIDRES	1.956255	Hz
AQ	0.2557652	sec
RG	456.1	
DW	124.800	usec
DE	6.50	usec
TE	296.5	K
D0	0.00000300	sec
D1	1.50000000	sec
D13	0.00000400	sec
D16	0.00020000	sec
IN0	0.00024960	sec

======== CHANNEL f1 ========

NUC1	1H	
P0	7.05	usec
P1	7.05	usec
PL1	5.00	dB
SFO1	500.2320009	MHz

====== GRADIENT CHANNEL =====

GPNAM1	SINE.100	
GPZ1	10.00	%
P16	1000.00	usec
ND0	1	
TD	256	
SFO1	500.232	MHz
FIDRES	15.650039	Hz
SW	8.009	ppm
FnMODE	QF	
SI	1024	
SF	500.2300193	MHz
WDW	QSINE	
SSB	0	
LB	0.00	Hz
GB	0	
PC	1.00	
SI	1024	
MC2	QF	
SF	500.2300193	MHz
WDW	QSINE	
SSB	0	
LB	0.00	Hz
GB	0	

COMPOUND 2
HMBC

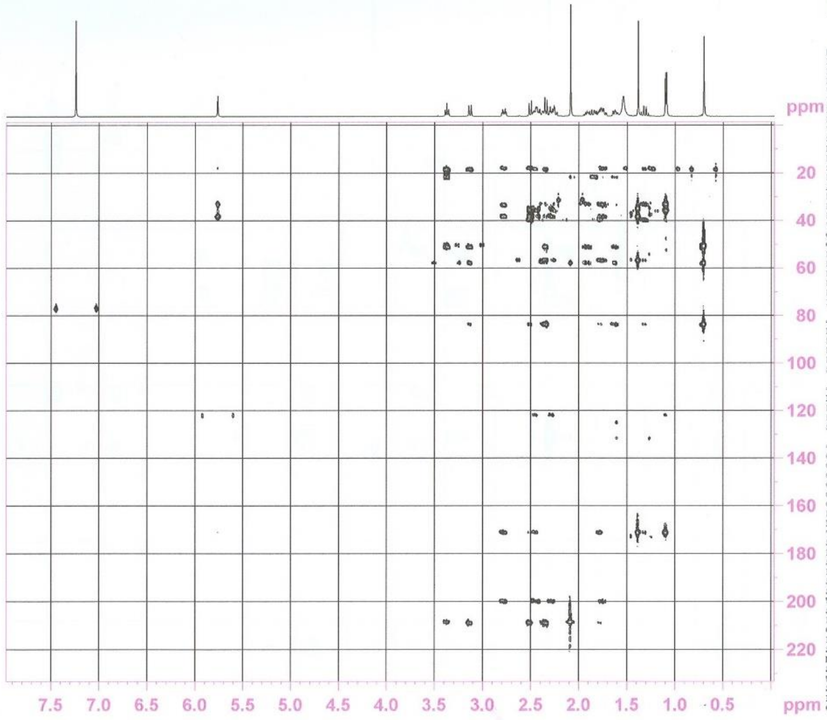

BRUKER

NAME	nov08-12	
EXPNO	6	
PROCNO	1	
Date_	20121109	
Time	1.26	
INSTRUM	spect	
PROBHD	5 mm PABBI 1H/	
PULPROG	hmbcgplpndqf	
TD	2048	
SOLVENT	CDCl3	
NS	64	
DS	8	
SWH	4006.410	Hz
FIDRES	1.956255	Hz
AQ	0.2557652	sec
RG	23170.5	
DW	124.800	usec
DE	6.50	usec
TE	297.6	K
CNST2	145.0000000	
CNST13	10.0000000	
D0	0.00000300	sec
D1	2.00000000	sec
D2	0.00344828	sec
D6	0.05000000	sec
D16	0.00020000	sec
IN0	0.00001690	sec

======== CHANNEL f1 ========

NUC1	1H	
P1	7.05	usec
P2	14.10	usec
PL1	5.00	dB
SFO1	500.2320009	MHz

======== CHANNEL f2 ========

NUC2	13C	
P3	15.25	usec
PL2	-3.00	dB
SFO2	125.7974871	MHz

====== GRADIENT CHANNEL =====

GPNAM1	SINE.100	
GPNAM2	SINE.100	
GPNAM3	SINE.100	
GPZ1	50.00	%
GPZ2	30.00	%
GPZ3	40.10	%
P16	1000.00	usec
ND0	2	
TD	256	
SFO1	125.7975	MHz
FIDRES	115.478165	Hz
SW	235.000	ppm
FnMODE	QF	
SI	1024	
SF	500.2300193	MHz
WDW	SINE	
SSB	0	
LB	0.00	Hz
GB	0	
PC	1.00	
SI	512	
MC2	QF	
SF	125.7829370	MHz
WDW	SINE	
SSB	0	
LB	0.00	Hz
GB	0	

COMPOUND 2
NOESY

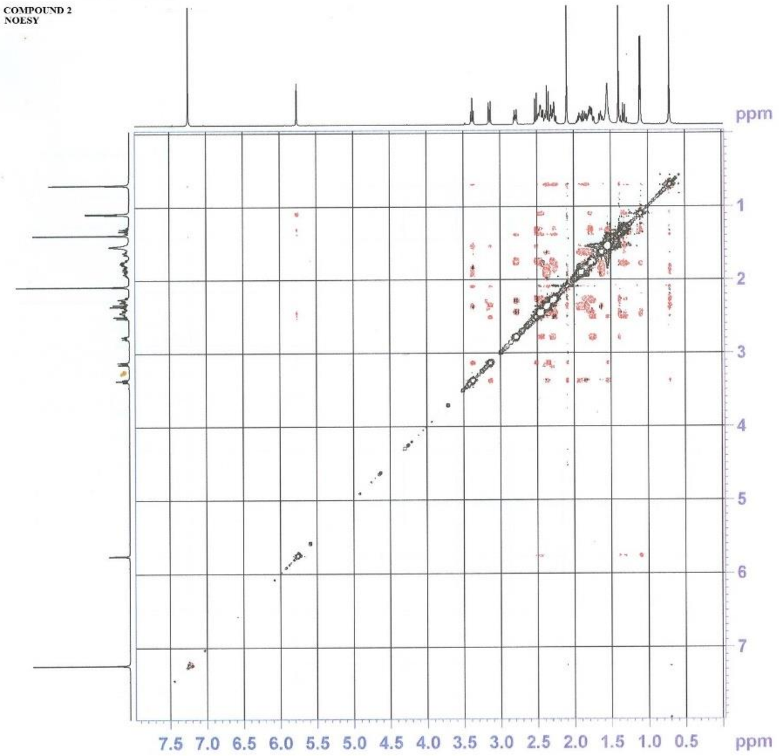

BRUKER

NAME	nov08-12
EXPNO	4
PROCNO	1
Date_	20121108
Time	17.34
INSTRUM	spect
PROBHD	5 mm PABBI 1H/
PULPROG	noesygpph
TD	2048
SOLVENT	CDCl3
NS	16
DS	8
SWH	4006.410 Hz
FIDRES	1.956255 Hz
AQ	0.2557652 sec
RG	1149.4
DW	124.800 usec
DE	6.50 usec
TE	297.0 K
D0	0.00011582 sec
D1	1.50000000 sec
D8	0.80000001 sec
D16	0.00020000 sec
IN0	0.00024960 sec

======== CHANNEL f1 ========

NUC1	1H
P1	7.05 usec
P2	14.10 usec
PL1	5.00 dB
SFO1	500.2320009 MHz

====== GRADIENT CHANNEL =====

GPNAM1	SINE.100
GPNAM2	SINE.100
GPZ1	40.00 %
GPZ2	-40.00 %
P16	1000.00 usec
ND0	1
TD	256
SFO1	500.232 MHz
FIDRES	15.650039 Hz
SW	8.009 ppm
FnMODE	States-TPPI
SI	1024
SF	500.2300193 MHz
WDW	QSINE
SSB	2
LB	0.00 Hz
GB	0
PC	1.00
SI	1024
MC2	States-TPPI
SF	500.2300193 MHz
WDW	QSINE
SSB	2
LB	0.00 Hz
GB	0
