# Supplementary material for: New Anti-Inflammatory Metabolites by Microbial Transformation of Medrysone
Source: PLoS One. 2016 Apr 22;11(4):e0153951. doi: 10.1371/journal.pone.0153951 (PMC4841542; doi:10.1371/journal.pone.0153951)
Supplement: S3 File — (PDF) [file pone.0153951.s003.pdf]

COMPOUND 3

Date Run: 10-05-2012 (Time Run: 10:01:56)

Instrument: JEOL MSRoute

Run By: HEJ

Inlet: Direct Probe

Ionization mode: EI+

Scan: 21

R.T.: 1.78

Base: m/z 315; 53.6%FS TIC: 2824128

#Ions: 100

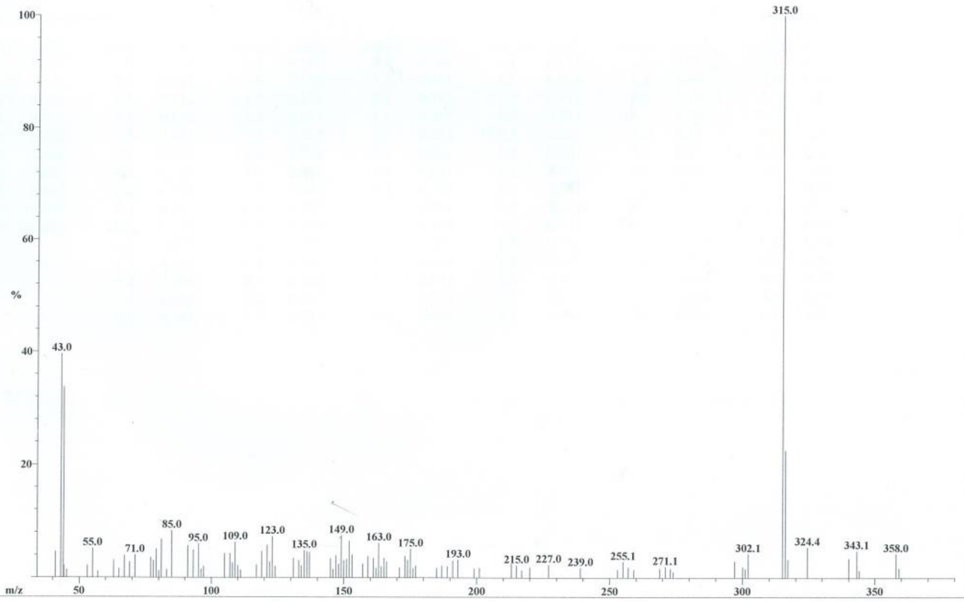

COMPOUND 3
HREI-MS

Mass	Relative Intensity	Theoretical Mass	Delta [mmu]	RDB	Composition
152.0781	1.7	152.0832	-5.1	4.0	$C_{9} H_{12} O_{2}$
154.9920	3.3				
157.1015	1.7	157.1012	0.4	6.5	$C_{12} H_{13}$
159.0800	3.3	159.0804	-0.5	6.5	$C_{11} H_{11} O_{1}$
161.0945	2.1	161.0961	-1.6	5.5	$C_{11} H_{13} O_{1}$
161.9919	4.0	161.9948	-2.8	8.0	$C_{8} H_{2} O_{4}$
163.1141	1.2	163.1117	2.3	4.5	$C_{11} H_{15} O_{1}$
165.0721	1.2	165.0699	2.3	9.5	$C_{13} H_{9}$
165.0947	1.0	165.0910	3.6	4.5	$C_{10} H_{13} O_{2}$
168.9888	18.4	168.9920	-3.2	10.5	$C_{10} H_{1} O_{3}$
173.0945	2.8	173.0961	-1.6	6.5	$C_{12} H_{13} O_{1}$
174.1065	6.1	174.1039	2.6	6.0	$C_{12} H_{14} O_{1}$
175.1076	4.2	175.1117	-4.1	5.5	$C_{12} H_{15} O_{1}$
176.1195	1.0	176.1196	-0.1	5.0	$C_{12} H_{16} O_{1}$
180.9888	21.7	180.9920	-3.2	11.5	$C_{11} H_{1} O_{3}$
181.9897	1.1				
185.9926	1.6	185.9948	-2.2	10.0	$C_{10} H_{2} O_{4}$
187.1140	1.9	187.1117	2.2	6.5	$C_{13} H_{15} O_{1}$
192.9888	5.7	192.9920	-3.2	12.5	$C_{12} H_{1} O_{3}$
193.1212	2.2	193.1223	-1.1	4.5	$C_{12} H_{17} O_{2}$
199.1171	1.4	199.1117	5.3	7.5	$C_{14} H_{15} O_{1}$
204.9888	3.4	204.9920	-3.2	13.5	$C_{13} H_{1} O_{3}$
211.1108	1.1	211.1117	-1.0	8.5	$C_{15} H_{15} O_{1}$
211.9924	1.3	211.9893	3.1	16.0	$C_{15} O_{2}$
213.1273	2.2	213.1274	-0.1	7.5	$C_{15} H_{17} O_{1}$
216.9896	1.4	216.9920	-2.4	14.5	$C_{14} H_{1} O_{3}$
218.9856	6.8				
227.1451	1.2	227.1430	2.0	7.5	$C_{16} H_{19} O_{1}$
230.9856	10.8				
235.9866	1.2	235.9893	-2.7	18.0	$C_{17} O_{2}$
237.1296	1.2	237.1274	2.2	9.5	$C_{17} H_{17} O_{1}$
238.1432	1.1				
239.1415	1.6	239.1430	-1.6	8.5	$C_{17} H_{19} O_{1}$
242.9856	14.3				
243.9825	1.0	243.9791	3.4	16.0	$C_{15} O_{4}$
253.1642	1.9	253.1587	5.5	8.5	$C_{18} H_{21} O_{1}$
254.9856	3.5				
255.1405	1.9	255.1380	2.5	8.5	$C_{17} H_{19} O_{2}$
255.1686	1.7	255.1743	-5.7	7.5	$C_{18} H_{23} O_{1}$
266.9924	1.3				
268.9824	1.9	268.9869	-4.5	17.5	$C_{17} H_{1} O_{4}$
269.1488	1.0	269.1536	-4.8	8.5	$C_{18} H_{21} O_{2}$
279.1740	1.4	279.1743	-0.4	9.5	$C_{20} H_{23} O_{1}$
280.9824	6.7	280.9869	-4.5	18.5	$C_{18} H_{1} O_{4}$
292.9824	14.8	292.9869	-4.5	19.5	$C_{19} H_{1} O_{4}$
296.1757	1.3	296.1771	-1.4	9.0	$C_{20} H_{24} O_{2}$
297.1898	1.6	297.1849	4.9	8.5	$C_{20} H_{25} O_{2}$
304.9824	2.2	304.9869	-4.5	20.5	$C_{20} H_{1} O_{4}$
315.1971	16.2	315.1955	1.6	7.5	$C_{20} H_{27} O_{3}$
316.2055	3.8	316.2033	2.2	7.0	$C_{20} H_{28} O_{3}$
316.9834	1.3	316.9869	-3.5	21.5	$C_{21} H_{1} O_{4}$
325.1941	1.4	325.1951	-1.0	13.5	$C_{25} H_{25}$
330.9792	4.1				
340.1995	8.3	340.2033	-3.8	9.0	$C_{22} H_{28} O_{3}$
341.2047	1.7				
342.9792	5.4				
343.1874	1.1	343.1904	-3.0	8.5	$C_{21} H_{27} O_{4}$
354.9792	1.5				
358.2115	1.3	358.2139	-2.4	8.0	$C_{22} H_{30} O_{4}$
380.9760	2.1				
392.9760	3.8				
404.9760	1.5				
442.9728	1.9				
492.9697	1.1				

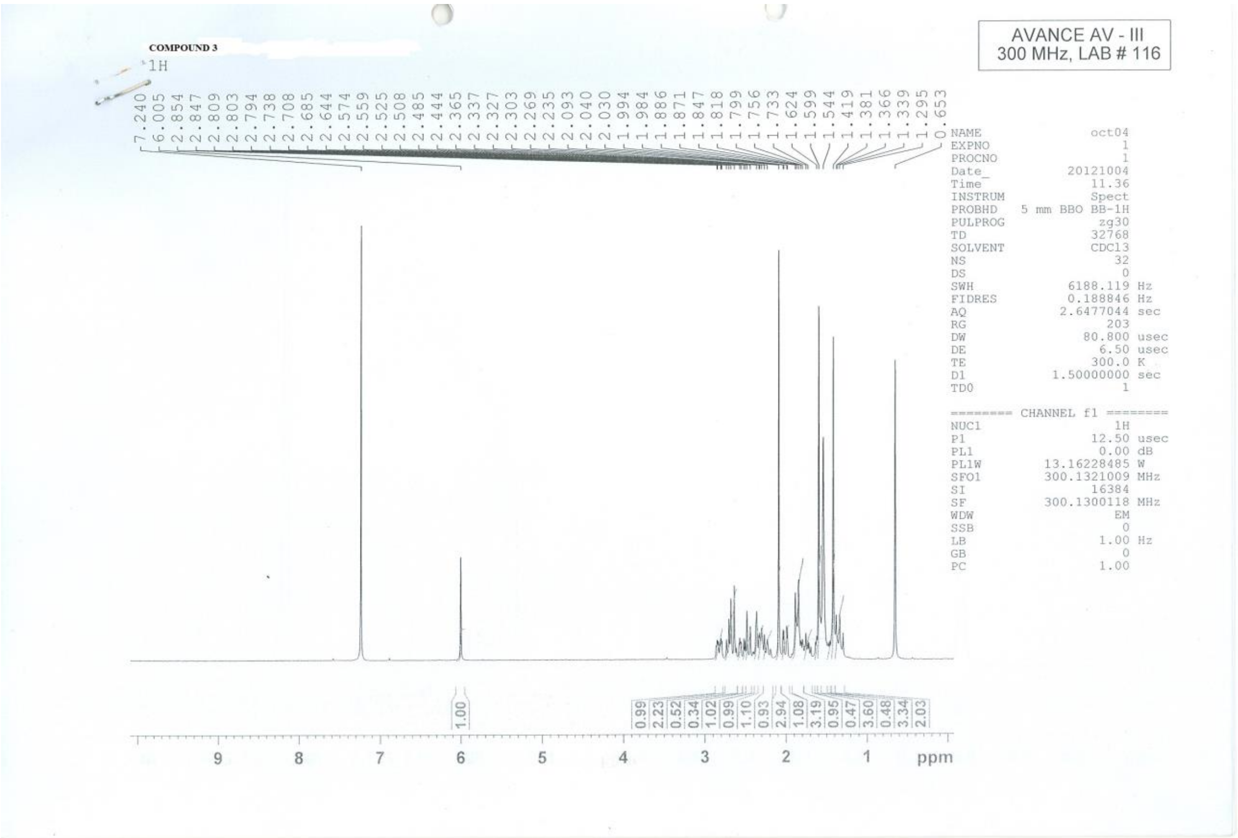
AVANCE AV - III
300 MHz, LAB # 116
COMPOUND 3
1H
7.240
6.005
2.854
2.847
2.809
2.803
2.794
2.738
2.708
2.685
2.644
2.574
2.559
2.525
2.508
2.485
2.444
2.365
2.337
2.327
2.303
2.269
2.235
2.093
2.040
2.030
1.994
1.984
1.886
1.871
1.847
1.818
1.799
1.756
1.733
1.624
1.599
1.544
1.419
1.381
1.366
1.339
1.295
0.653
NAME oct04
EXPNO 1
PROCNO 1
Date_ 20121004
Time 11.36
INSTRUM Spect
PROBHD 5 mm BBO BB-1H
PULPROG zg30
TD 32768
SOLVENT CDCl3
NS 32
DS 0
SWH 6188.119 Hz
FIDRES 0.188846 Hz
AQ 2.6477044 sec
RG 203
DW 80.800 usec
DE 6.50 usec
TE 300.0 K
D1 1.50000000 sec
TD0 1
======== CHANNEL f1 ========
NUC1 1H
P1 12.50 usec
PL1 0.00 dB
PL1W 13.16228485 W
SFO1 300.1321009 MHz
SI 16384
SF 300.1300118 MHz
WDW EM
SSB 0
LB 1.00 Hz
GB 0
PC 1.00
1.00
0.99
2.23
0.52
0.34
1.02
0.99
1.10
0.93
2.94
1.08
3.19
0.95
0.47
3.60
0.48
3.34
2.03
9
8
7
6
5
4
3
2
1
ppm

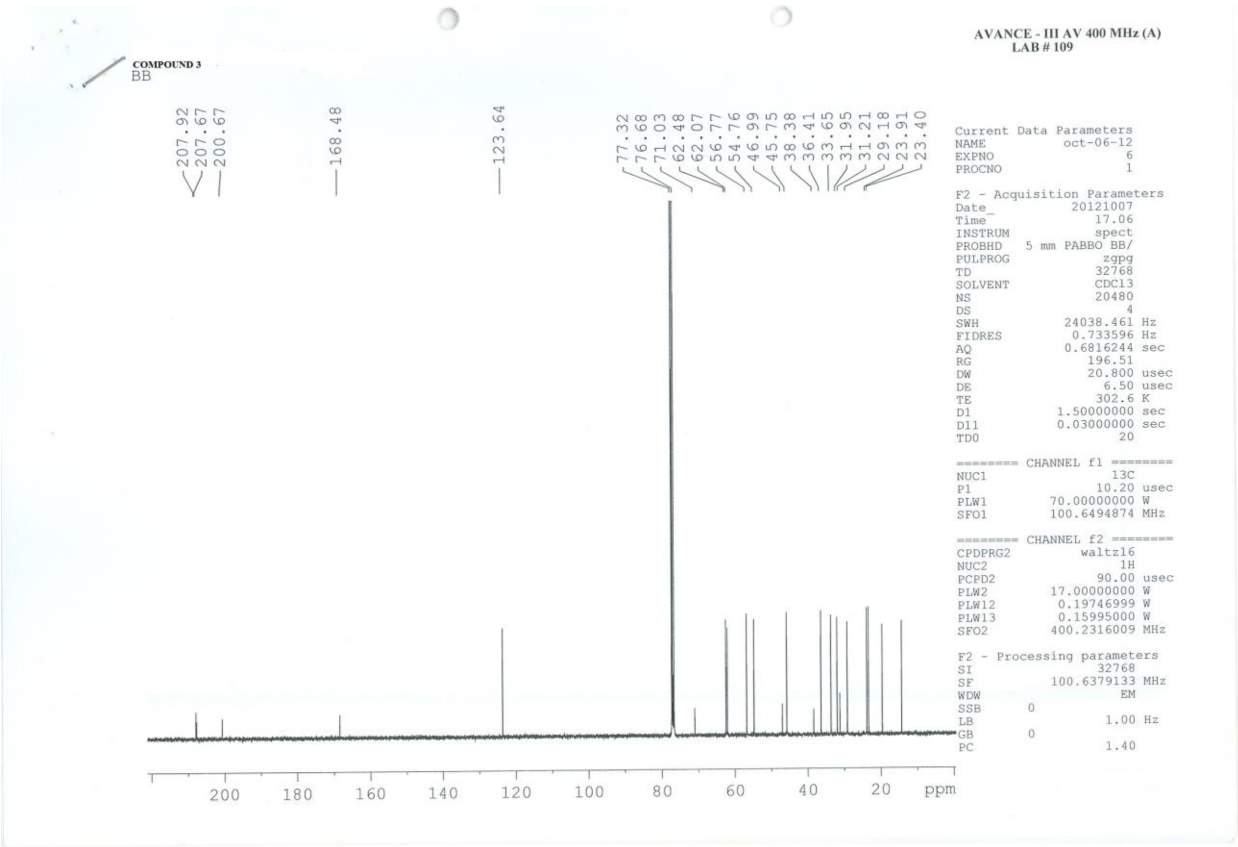
AVANCE - III AV 400 MHz (A)
LAB # 109
COMPOUND 3
BB
207.92
207.67
200.67
168.48
123.64
77.32
76.68
71.03
62.48
62.07
56.77
54.76
46.99
45.75
38.38
36.41
33.65
31.95
31.21
29.18
23.91
23.40
Current Data Parameters
NAME oct-06-12
EXPNO 6
PROCNO 1
F2 - Acquisition Parameters
Date_ 20121007
Time 17.06
INSTRUM spect
PROBHD 5 mm PABBO BB/
PULPROG zgpg
TD 32768
SOLVENT CDCl3
NS 20480
DS 4
SWH 24038.461 Hz
FIDRES 0.733596 Hz
AQ 0.6816244 sec
RG 196.51
DW 20.800 usec
DE 6.50 usec
TE 302.6 K
D1 1.50000000 sec
D11 0.03000000 sec
TD0 20
======== CHANNEL f1 ========
NUC1 13C
P1 10.20 usec
PLW1 70.00000000 W
SFO1 100.6494874 MHz
======== CHANNEL f2 ========
CPDPRG2 waltz16
NUC2 1H
PCPD2 90.00 usec
PLW2 17.00000000 W
PLW12 0.19746999 W
PLW13 0.15995000 W
SFO2 400.2316009 MHz
F2 - Processing parameters
SI 32768
SF 100.6379133 MHz
WDW EM
SSB 0
LB 1.00 Hz
GB 0
PC 1.40
200
180
160
140
120
100
80
60
40
20
ppm

COMPOUND 3
DEPT 135

AVANCE - III AV 400 MHz (A)
LAB # 109

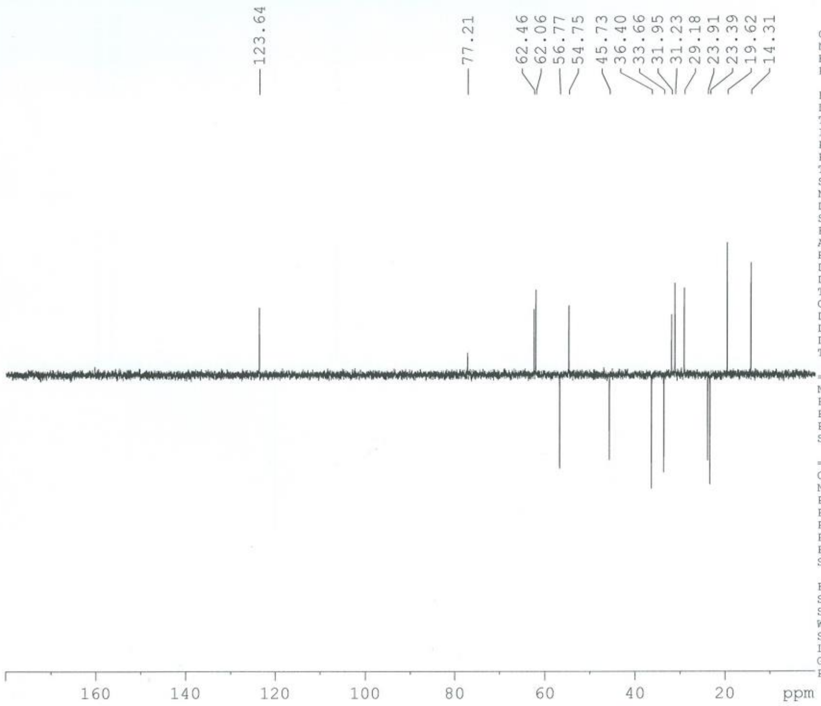


```
Current Data Parameters
NAME          oct-06-12
EXPNO                 7
PROCNO                1

F2 - Acquisition Parameters
Date_          20121008
Time               5.56
INSTRUM           spect
PROBHD   5 mm PABBO BB/
PULPROG         dept135
TD                32768
SOLVENT           CDCl3
NS                 4345
DS                    8
SWH           18115.941 Hz
FIDRES         0.552855 Hz
AQ            0.9044468 sec
RG               196.51
DW               27.600 usec
DE                 6.50 usec
TE                302.1 K
CNST2       145.0000000
D1           1.50000000 sec
D2           0.00344828 sec
D12          0.00002000 sec
TD0                  10

======== CHANNEL f1 ========
NUC1                13C
P1                10.20 usec
P2                20.40 usec
PLW1        70.00000000 W
SFO1        100.6469714 MHz

======== CHANNEL f2 ========
CPDPRG2         waltz16
NUC2                 1H
P3                 9.70 usec
P4                19.40 usec
PCPD2             90.00 usec
PLW2        17.00000000 W
PLW12        0.19746999 W
SFO2        400.2312800 MHz

F2 - Processing parameters
SI                16384
SF          100.6379133 MHz
WDW                  EM
SSB      0
LB                 1.00 Hz
GB       0
PC                 1.40
```

COMPOUND 3
DEPT 90

AVANCE - III AV 400 MHz (A)
LAB # 109

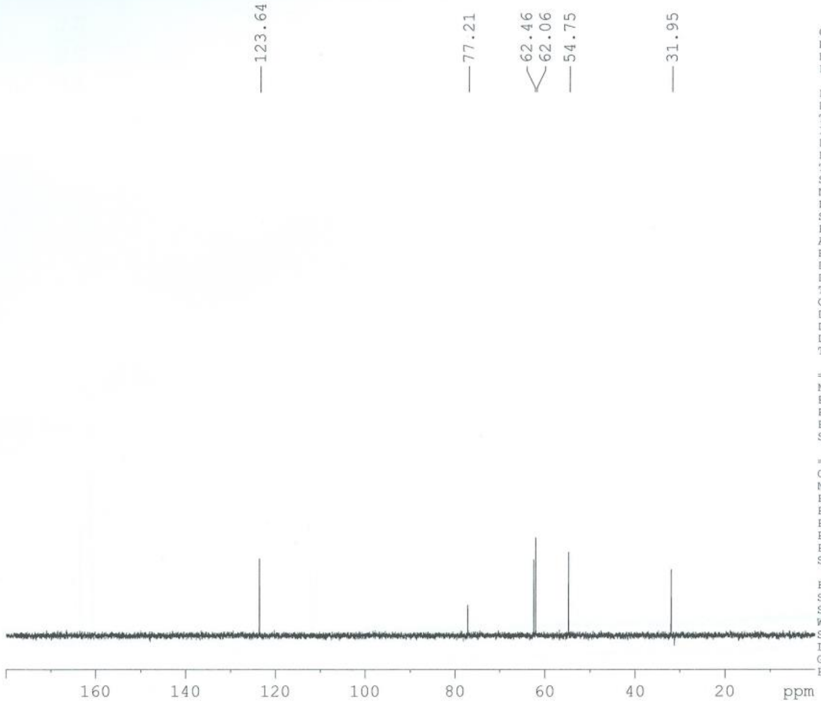


```
Current Data Parameters
NAME          oct-06-12
EXPNO                 8
PROCNO                1

F2 - Acquisition Parameters
Date_          20121008
Time               8.54
INSTRUM           spect
PROBHD   5 mm PABBO BB/
PULPROG          dept90
TD                32768
SOLVENT           CDCl3
NS                 4096
DS                    4
SWH           18115.941 Hz
FIDRES         0.552855 Hz
AQ            0.9044468 sec
RG               196.51
DW               27.600 usec
DE                 6.50 usec
TE                301.8 K
CNST2       145.0000000
D1           1.50000000 sec
D2           0.00344828 sec
D12          0.00002000 sec
TD0                   4

======== CHANNEL f1 ========
NUC1                13C
P1                10.20 usec
P2                20.40 usec
PLW1        70.00000000 W
SFO1        100.6469714 MHz

======== CHANNEL f2 ========
CPDPRG2         waltz16
NUC2                 1H
P3                 9.70 usec
P4                19.40 usec
PCPD2             90.00 usec
PLW2        17.00000000 W
PLW12        0.19746999 W
SFO2        400.2312800 MHz

F2 - Processing parameters
SI                16384
SF          100.6379133 MHz
WDW                  EM
SSB      0
LB                 1.00 Hz
GB       0
PC                 1.40
```

COMPOUND 3
HSQC

AVANCE - III AV 400 MHz (A)
LAB # 109

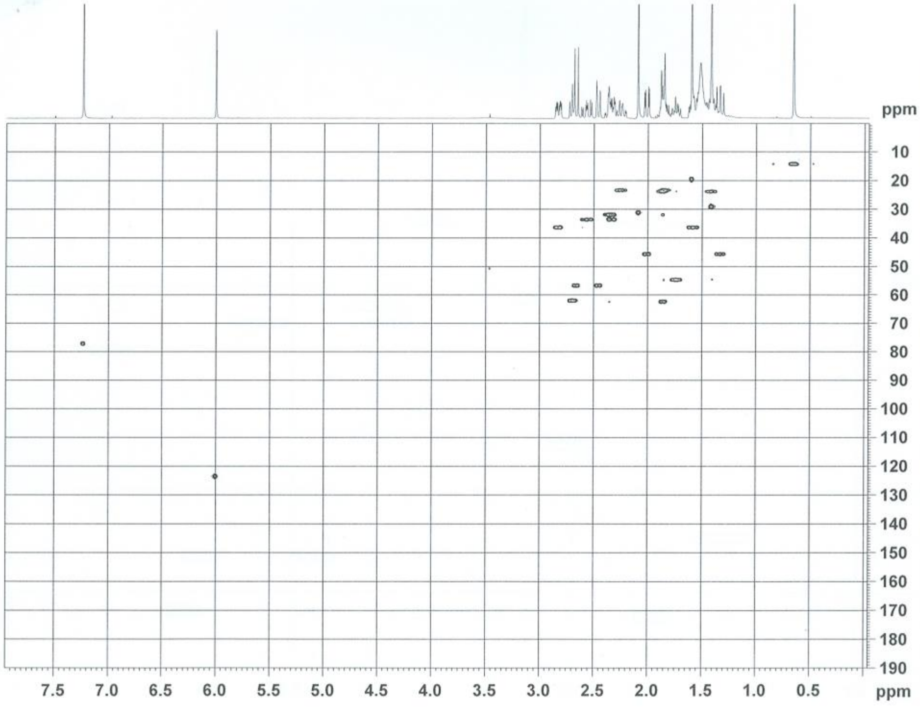


```
Current Data Parameters
NAME            oct-06-12
EXPNO                   4
PROCNO                  1

F2 - Acquisition Parameters
Date_            20121006
Time                21.58
INSTRUM             spect
PROBHD   5 mm PABBO BB/
PULPROG         hsqcetgpsi
TD                   1024
SOLVENT             CDCl3
NS                     64
DS                     16
SWH              3201.024 Hz
FIDRES           3.126000 Hz
AQ              0.1599988 sec
RG                 196.51
DW                156.200 usec
DE                   6.50 usec
TE                  301.7 K
CNST2         145.0000000
D0             0.00000300 sec
D1             1.50000000 sec
D4             0.00172414 sec
D11            0.03000000 sec
D16            0.00020000 sec
D24            0.00110000 sec
IN0            0.00002615 sec
ZGOPTNS

======== CHANNEL f1 ========
NUC1                   1H
P1                   9.70 usec
P2                  19.40 usec
P28                  1.00 usec
PLW1          17.00000000 W
SFO1          400.2316009 MHz

======== CHANNEL f2 ========
CPDPRG2              garp
NUC2                  13C
P3                  10.20 usec
P4                  20.40 usec
PCPD2               80.00 usec
PLW2          70.00000000 W
PLW12          1.13789999 W
SFO2          100.6474746 MHz

====== GRADIENT CHANNEL =====
GPNAM1         SMSQ10.100
GPNAM2         SMSQ10.100
GPZ1                80.00 %
GPZ2                20.10 %
P16               1000.00 usec

F1 - Acquisition parameters
TD                    256
SFO1             100.6475 MHz
FIDRES          74.697769 Hz
SW                189.996 ppm
FnMODE      Echo-Antiecho

F2 - Processing parameters
SI                   1024
SF            400.2300198 MHz
WDW                 QSINE
SSB                     2
LB        0 Hz
GB        0
PC                   1.00

F1 - Processing parameters
SI                   1024
MC2         echo-antiecho
SF            100.6379133 MHz
WDW
SSB                     2
LB        0 Hz
GB        0
```

COMPOUND 3
COSY

AVANCE - III AV 400 MHz (A)
LAB # 109

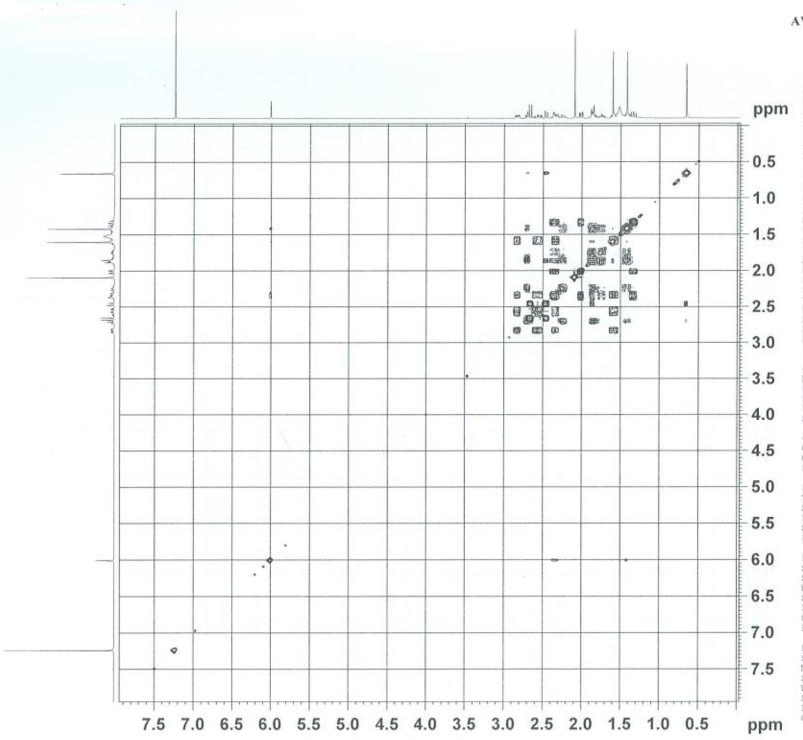


```
Current Data Parameters
NAME            oct-06-12
EXPNO                   2
PROCNO                  1

F2 - Acquisition Parameters
Date_            20121006
Time                11.39
INSTRUM             spect
PROBHD   5 mm PABBO BB/
PULPROG          cosygpqf
TD                   2048
SOLVENT             CDCl3
NS                     32
DS                      8
SWH              3201.024 Hz
FIDRES           1.563000 Hz
AQ              0.3199476 sec
RG                 196.51
DW                156.200 usec
DE                   6.50 usec
TE                  301.8 K
D0             0.00000300 sec
D1             1.50000000 sec
D13            0.00000400 sec
D16            0.00020000 sec
IN0            0.00031240 sec

======== CHANNEL f1 ========
NUC1                   1H
P0                   9.70 usec
P1                   9.70 usec
PLW1          17.00000000 W
SFO1          400.2316009 MHz

====== GRADIENT CHANNEL =====
GPNAM1         SMSQ10.100
GPZ1                10.00 %
P16               1000.00 usec

F1 - Acquisition parameters
TD                    256
SFO1             400.2316 MHz
FIDRES          12.504000 Hz
SW                  7.998 ppm
FnMODE                 QF

F2 - Processing parameters
SI                   1024
SF            400.2300198 MHz
WDW                 QSINE
SSB       0
LB        0 Hz
GB        0
PC                   1.00

F1 - Processing parameters
SI                   1024
MC2                    QF
SF            400.2300198 MHz
WDW           States-TPPI
SSB       0
LB        0 Hz
GB        0
```

COMPOUND 3

HMBC

AVANCE - III AV 400 MHz (A)
LAB # 109

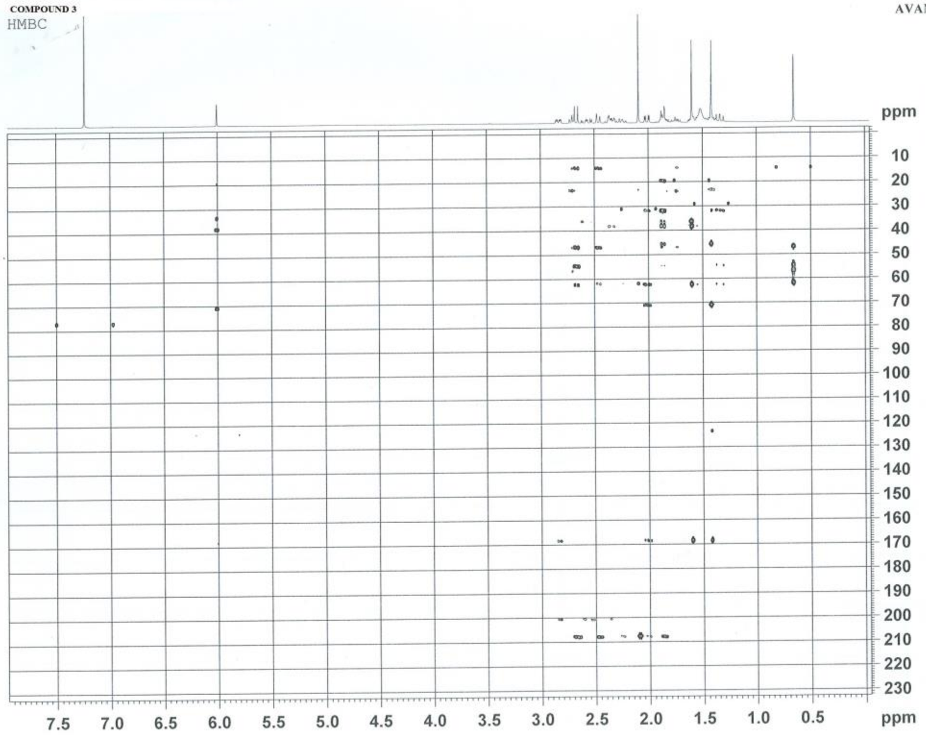


```
Current Data Parameters
NAME            oct-06-12
EXPNO                   5
PROCNO                  1

F2 - Acquisition Parameters
Date_            20121007
Time                 5.38
INSTRUM             spect
PROBHD   5 mm PABBO BB/
PULPROG      hmbcgplpndqf
TD                   2048
SOLVENT             CDCl3
NS                     64
DS                     16
SWH              3201.024 Hz
FIDRES           1.563000 Hz
AQ              0.3199476 sec
RG                 196.51
DW                156.200 usec
DE                   6.50 usec
TE                  302.2 K
CNST2         145.0000000
CNST13         10.0000000
D0             0.00000300 sec
D1             2.00000000 sec
D2             0.00344828 sec
D6             0.05000000 sec
D16            0.00020000 sec
IN0            0.00002115 sec

======== CHANNEL f1 ========
NUC1                   1H
P1                   9.70 usec
P2                  19.40 usec
PLW1          17.00000000 W
SFO1          400.2316009 MHz

======== CHANNEL f2 ========
NUC2                  13C
P3                  10.20 usec
PLW2          70.00000000 W
SFO2          100.6494874 MHz

====== GRADIENT CHANNEL =====
GPNAM1         SMSQ10.100
GPNAM2         SMSQ10.100
GPNAM3         SMSQ10.100
GPZ1                50.00 %
GPZ2                30.00 %
GPZ3                40.10 %
P16               1000.00 usec

F1 - Acquisition parameters
TD                    256
SFO1             100.6495 MHz
FIDRES          92.391670 Hz
SW                234.996 ppm
FnMODE                 QF

F2 - Processing parameters
SI                   2048
SF            400.2300198 MHz
WDW                  SINE
SSB      0
LB       0 Hz
GB       0
PC                   1.00

F1 - Processing parameters
SI                   1024
MC2                    QF
SF            100.6379133 MHz
WDW                States
SSB      0
LB       0 Hz
GB       0
```

COMPOUND 3

NOESY

AVANCE - III AV 400 MHz (A)
LAB # 109

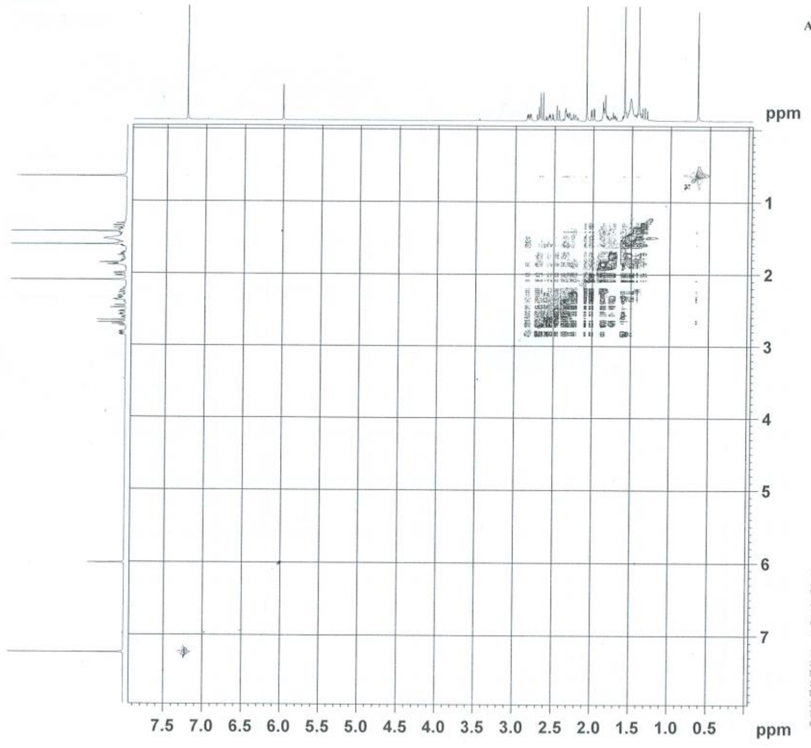


```
Current Data Parameters
NAME            oct-06-12
EXPNO                   3
PROCNO                  1

F2 - Acquisition Parameters
Date_            20121006
Time                15.56
INSTRUM             spect
PROBHD   5 mm PABBO BB/
PULPROG         noesygpph
TD                   2048
SOLVENT             CDCl3
NS                     32
DS                     32
SWH              3201.024 Hz
FIDRES           1.563000 Hz
AQ              0.3199476 sec
RG                 112.56
DW                156.200 usec
DE                   6.50 usec
TE                  301.9 K
D0             0.00014385 sec
D1             1.50000000 sec
D8             0.80000001 sec
D16            0.00020000 sec
IN0            0.00031240 sec

======== CHANNEL f1 ========
NUC1                   1H
P1                   9.70 usec
P2                  19.40 usec
PLW1          17.00000000 W
SFO1          400.2316009 MHz

====== GRADIENT CHANNEL =====
GPNAM1           SINE.100
GPZ1                40.00 %
P16               1000.00 usec

F1 - Acquisition parameters
TD                    256
SFO1             400.2316 MHz
FIDRES          12.504000 Hz
SW                  7.998 ppm
FnMODE        States-TPPI

F2 - Processing parameters
SI                   1024
SF            400.2300198 MHz
WDW                 QSINE
SSB                     2
LB       0 Hz
GB       0
PC                   1.00

F1 - Processing parameters
SI                   1024
MC2           States-TPPI
SF            400.2300198 MHz
WDW
SSB                     2
LB       0 Hz
GB       0
```

COMPOUND 3
NOESY

AVANCE AV-500
LAB NO: 109B

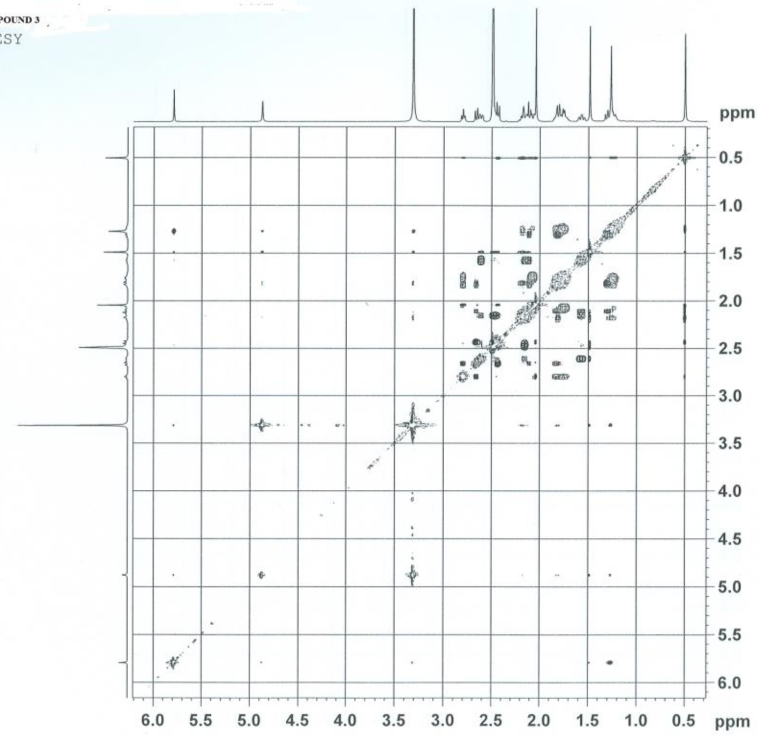

NAME	feb24-15	
EXPNO	12	
PROCNO	1	
Date_	20150224	
Time	15.59	
INSTRUM	spect	
PROBHD	5 mm BBI 1H/D-	
PULPROG	noesygpph	
TD	2048	
SOLVENT	DMSO	
NS	16	
DS	2	
SWH	3255.208	Hz
FIDRES	1.589457	Hz
AQ	0.3147764	sec
RG	512	
DW	153.600	usec
DE	6.50	usec
TE	298.5	K
D0	0.00014341	sec
D1	2.00000000	sec
D8	0.80000001	sec
D16	0.00020000	sec
IN0	0.00030720	sec

======== CHANNEL f1 ========

NUC1	1H	
P1	8.00	usec
P2	16.00	usec
PL1	-1.00	dB
SFO1	500.1316004	MHz

====== GRADIENT CHANNEL =====

GPNAM1	SINE.100	
GPZ1	40.00	%
P16	1000.00	usec
ND0	1	
TD	256	
SFO1	500.1316	MHz
FIDRES	12.715656	Hz
SW	6.509	ppm
FnMODE	States-TPPI	
SI	1024	
SF	500.1300106	MHz
WDW	QSINE	
SSB	2	
LB	0.00	Hz
GB	0	
PC	4.00	
SI	1024	
MC2	States-TPPI	
SF	500.1300106	MHz
WDW	QSINE	
SSB	2	
LB	0.00	Hz
GB	0	
