# Supplementary material for: New Anti-Inflammatory Metabolites by Microbial Transformation of Medrysone
Source: PLoS One. 2016 Apr 22;11(4):e0153951. doi: 10.1371/journal.pone.0153951 (PMC4841542; doi:10.1371/journal.pone.0153951)
Supplement: S4 File — (PDF) [file pone.0153951.s004.pdf]

Date Run: 10-16-2012 (Time Run: 12:00:03)

COMPOUND 4

Instrument: JEOL MSRoute
Inlet: Direct Probe

Ionization mode: EI+

Run By: HEJ

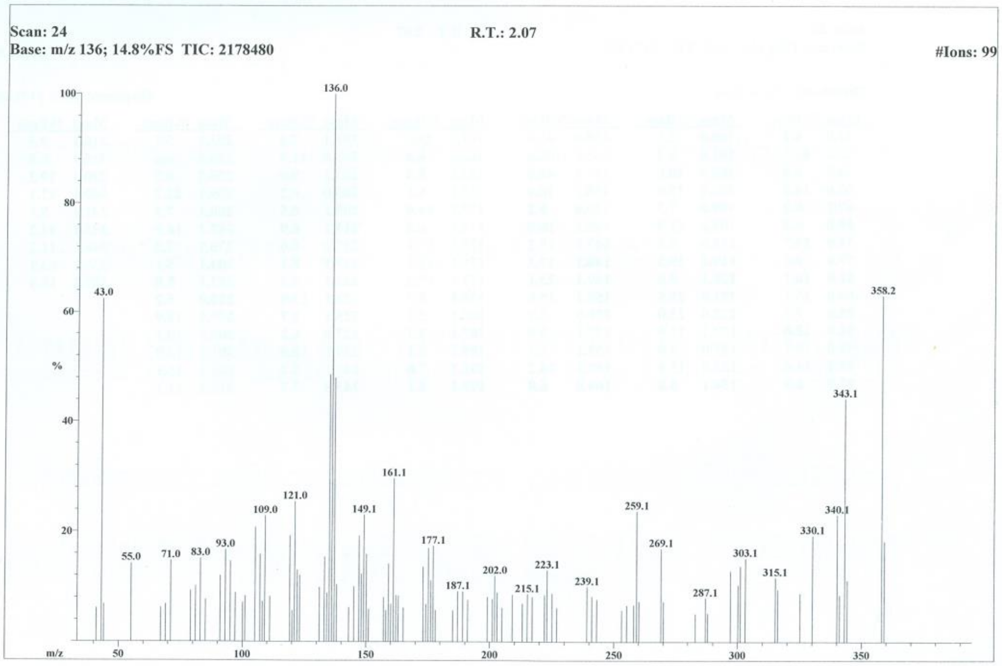

COMPOUND 4

Mass	Relative Intensity	Theoretical Mass	Delta [mmu]	RDB	Composition
242.9856	11.2				
251.1437	1.8	251.1430	0.7	9.5	$C_{18} H_{19} O_{1}$
		251.1489	-5.2	0.5	$C_{11} H_{23} O_{6}$
253.1583	1.7	253.1587	-0.4	8.5	$C_{18} H_{21} O_{1}$
254.1644	1.2	254.1665	-2.1	8.0	$C_{18} H_{22} O_{1}$
254.9856	3.4				
255.1684	2.7	255.1743	-5.9	7.5	$C_{18} H_{23} O_{1}$
258.1612	1.2	258.1614	-0.3	7.0	$C_{17} H_{22} O_{2}$
259.1678	3.1	259.1693	-1.4	6.5	$C_{17} H_{23} O_{2}$
265.1563	1.5	265.1587	-2.4	9.5	$C_{19} H_{21} O_{1}$
266.9853	1.6				
267.1434	1.0	267.1438	-0.4	0.5	$C_{11} H_{23} O_{7}$
		267.1380	5.5	9.5	$C_{18} H_{19} O_{2}$
268.9824	2.4	268.9869	-4.5	17.5	$C_{17} H_{1} O_{4}$
269.1546	4.6	269.1536	1.0	8.5	$C_{18} H_{21} O_{2}$
270.1585	2.2	270.1614	-2.9	8.0	$C_{18} H_{22} O_{2}$
279.1743	1.6	279.1743	-0.0	9.5	$C_{20} H_{23} O_{1}$
		279.1802	-5.9	0.5	$C_{13} H_{27} O_{6}$
280.9824	6.9	280.9869	-4.5	18.5	$C_{18} H_{1} O_{4}$
281.1584	1.3	281.1595	-1.1	0.5	$C_{12} H_{25} O_{7}$
		281.1536	4.8	9.5	$C_{19} H_{21} O_{2}$
282.1612	1.7	282.1614	-0.2	9.0	$C_{19} H_{22} O_{2}$
283.1695	1.9	283.1693	0.2	8.5	$C_{19} H_{23} O_{2}$
292.9824	12.3	292.9869	-4.5	19.5	$C_{19} H_{1} O_{4}$
293.9846	1.0	293.9795	5.1	15.0	$C_{15} H_{2} O_{7}$
295.1712	1.3	295.1693	2.0	9.5	$C_{20} H_{23} O_{2}$
		295.1751	-3.9	0.5	$C_{13} H_{27} O_{7}$
296.1801	1.3	296.1830	-2.8	0.0	$C_{13} H_{28} O_{7}$
		296.1771	3.0	9.0	$C_{20} H_{24} O_{2}$
297.1872	5.9	297.1849	2.3	8.5	$C_{20} H_{25} O_{2}$
298.1939	2.4	298.1927	1.2	8.0	$C_{20} H_{26} O_{2}$
300.1783	1.9				
301.1814	2.0	301.1798	1.5	7.5	$C_{19} H_{25} O_{3}$
304.9824	2.1	304.9869	-4.5	20.5	$C_{20} H_{1} O_{4}$
307.1691	1.2	307.1693	-0.2	10.5	$C_{21} H_{23} O_{2}$
315.1918	1.4	315.1955	-3.7	7.5	$C_{20} H_{27} O_{3}$
316.1784	1.1	316.1822	-3.8	12.0	$C_{23} H_{24} O_{1}$
316.9827	1.3	316.9869	-4.2	21.5	$C_{21} H_{1} O_{4}$
325.1817	2.0	325.1798	1.9	9.5	$C_{21} H_{25} O_{3}$
		325.1857	-3.9	0.5	$C_{14} H_{29} O_{8}$
330.2135	1.6	330.2189	-5.5	7.0	$C_{21} H_{30} O_{3}$
330.9792	3.6				
338.1862	1.8	338.1876	-1.4	10.0	$C_{22} H_{26} O_{3}$
340.2040	13.0	340.2033	0.7	9.0	$C_{22} H_{28} O_{3}$
		340.2092	-5.2	0.0	$C_{15} H_{32} O_{8}$
341.2046	3.5				
342.9792	4.0				
343.1908	2.0	343.1904	0.4	8.5	$C_{21} H_{27} O_{4}$
354.9792	1.5				
358.2128	4.0	358.2139	-1.0	8.0	$C_{22} H_{30} O_{4}$
359.2164	1.1	359.2217	-5.3	7.5	$C_{22} H_{31} O_{4}$
381.0255	2.9				
393.1095	2.7	393.1121	-2.7	18.5	$C_{26} H_{17} O_{4}$

AVANCE AV - III
300 MHz, LAB # 116

COMPOUND 4
H-NMR

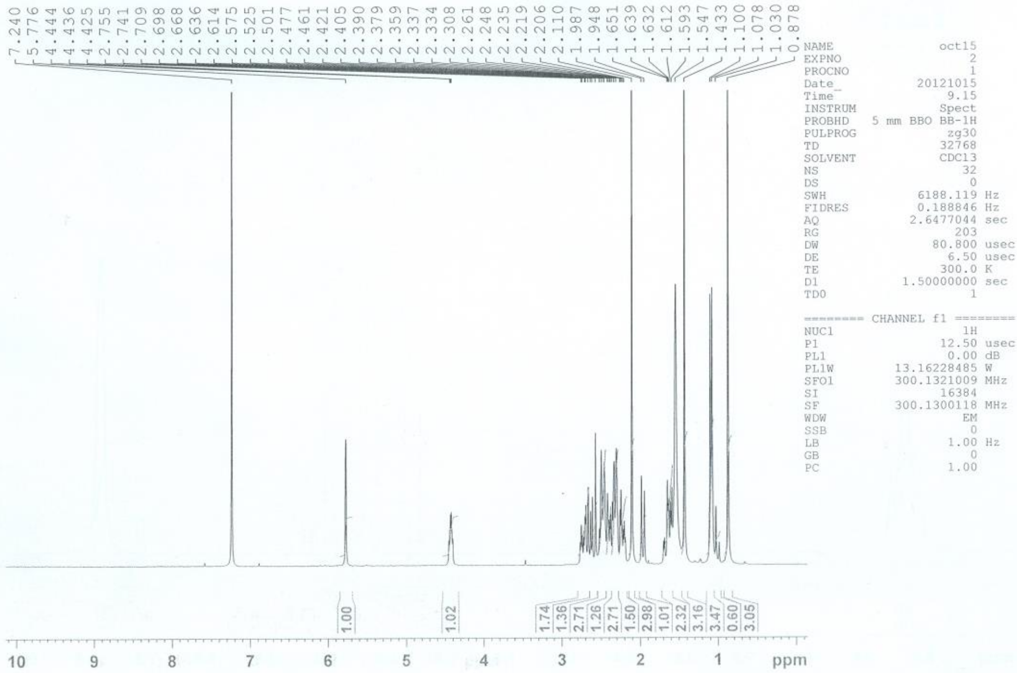

NAME	oct15
EXPNO	2
PROCNO	1
Date_	20121015
Time	9.15
INSTRUM	Spect
PROBHD	5 mm BBO BB-1H
PULPROG	zg30
TD	32768
SOLVENT	CDCl3
NS	32
DS	0
SWH	6188.119 Hz
FIDRES	0.188846 Hz
AQ	2.6477044 sec
RG	203
DW	80.800 usec
DE	6.50 usec
TE	300.0 K
D1	1.50000000 sec
TD0	1

======== CHANNEL f1 ========

NUC1	1H
P1	12.50 usec
PL1	0.00 dB
PL1W	13.16228485 W
SFO1	300.1321009 MHz
SI	16384
SF	300.1300118 MHz
WDW	EM
SSB	0
LB	1.00 Hz
GB	0
PC	1.00

H.E.J. Research Institute of Chemistry.

COMPOUND 4
BB

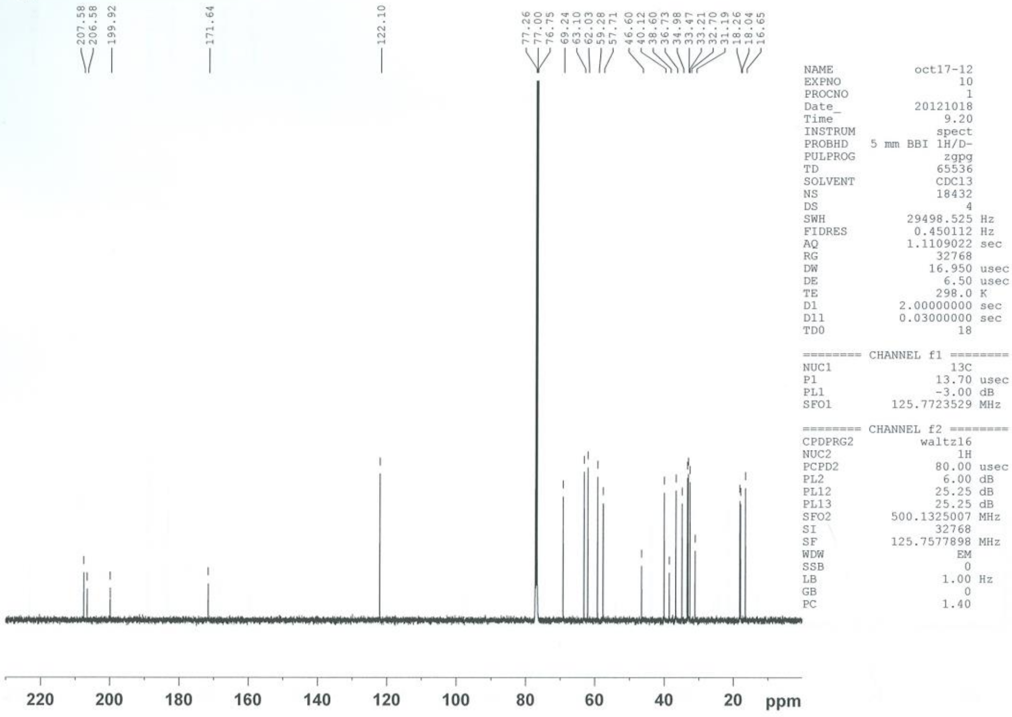

NAME	oct17-12
EXPNO	10
PROCNO	1
Date_	20121018
Time	9.20
INSTRUM	spect
PROBHD	5 mm BBI 1H/D-
PULPROG	zgpg
TD	65536
SOLVENT	CDCl3
NS	18432
DS	4
SWH	29498.525 Hz
FIDRES	0.450112 Hz
AQ	1.1109022 sec
RG	32768
DW	16.950 usec
DE	6.50 usec
TE	298.0 K
D1	2.00000000 sec
D11	0.03000000 sec
TD0	18

======== CHANNEL f1 ========

NUC1	13C
P1	13.70 usec
PL1	-3.00 dB
SFO1	125.7723529 MHz

======== CHANNEL f2 ========

CPDPRG2	waltz16
NUC2	1H
PCPD2	80.00 usec
PL2	6.00 dB
PL12	25.25 dB
PL13	25.25 dB
SFO2	500.1325007 MHz
SI	32768
SF	125.7577898 MHz
WDW	EM
SSB	0
LB	1.00 Hz
GB	0
PC	1.40

COMPOUND 4
DEPT135

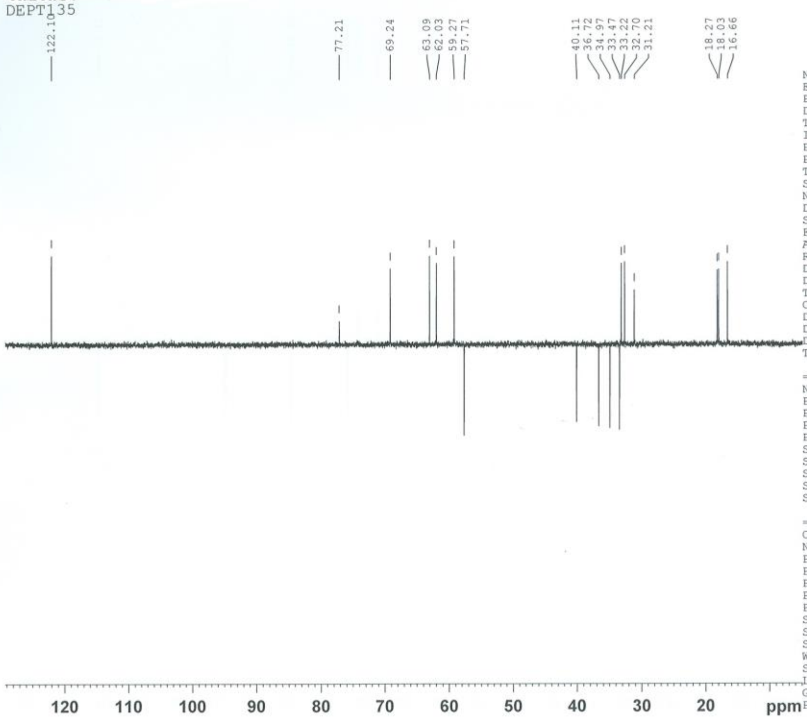

NAME oct17-12
EXPNO 11
PROCNO 1
Date_ 20121019
Time 1.19
INSTRUM spect
PROBHD 5 mm BBI 1H/D-
PULPROG deptsp135
TD 32768
SOLVENT CDCl3
NS 11951
DS 2
SWH 23809.523 Hz
FIDRES 0.726609 Hz
AQ 0.6881990 sec
RG 32768
DW 21.000 usec
DE 6.50 usec
TE 298.0 K
CNST2 145.0000000
D1 1.50000000 sec
D2 0.00344828 sec
D12 0.00002000 sec
TD0 12

======== CHANNEL f1 ========
NUC1 13C
P1 13.70 usec
P12 2000.00 usec
PL0 120.00 dB
PL1 -3.00 dB
SFO1 125.7697360 MHz
SP2 2.42 dB
SPNAM2 Crp60comp.4
SPOAL2 0.500
SPOFFS2 0.00 Hz

======== CHANNEL f2 ========
CPDPRG2 waltz16
NUC2 1H
P3 8.00 usec
P4 16.00 usec
PCPD2 80.00 usec
PL2 6.00 dB
PL12 25.25 dB
SFO2 500.1335009 MHz
SI 32768
SF 125.7577898 MHz
WDW EM
SSB 0
LB 1.00 Hz
GB 0
PC 1.40

COMPOUND 4
DEPT90

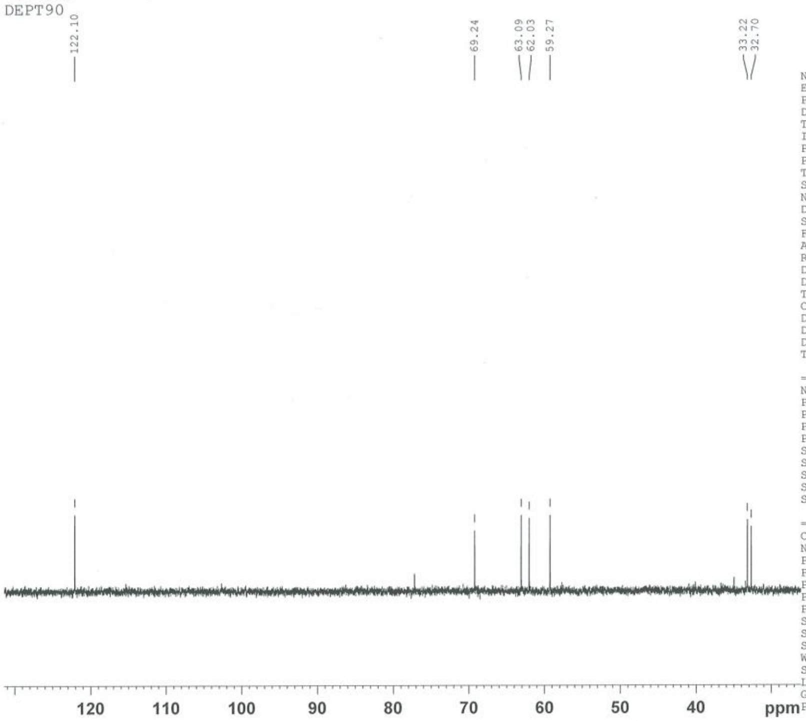

NAME oct17-12
EXPNO 12
PROCNO 1
Date_ 20121019
Time 8.44
INSTRUM spect
PROBHD 5 mm BBI 1H/D-
PULPROG deptsp90
TD 32768
SOLVENT CDCl3
NS 2068
DS 2
SWH 23809.523 Hz
FIDRES 0.726609 Hz
AQ 0.6881990 sec
RG 32768
DW 21.000 usec
DE 6.50 usec
TE 298.0 K
CNST2 145.0000000
D1 1.50000000 sec
D2 0.00344828 sec
D12 0.00002000 sec
TD0 8

======== CHANNEL f1 ========
NUC1 13C
P1 13.70 usec
P12 2000.00 usec
PL0 120.00 dB
PL1 -3.00 dB
SFO1 125.7697360 MHz
SP2 2.42 dB
SPNAM2 Crp60comp.4
SPOAL2 0.500
SPOFFS2 0.00 Hz

======== CHANNEL f2 ========
CPDPRG2 waltz16
NUC2 1H
P3 8.00 usec
P4 16.00 usec
PCPD2 80.00 usec
PL2 6.00 dB
PL12 25.25 dB
SFO2 500.1335009 MHz
SI 32768
SF 125.7577898 MHz
WDW EM
SSB 0
LB 1.00 Hz
GB 0
PC 1.40

COMPOUND 4
HSQC

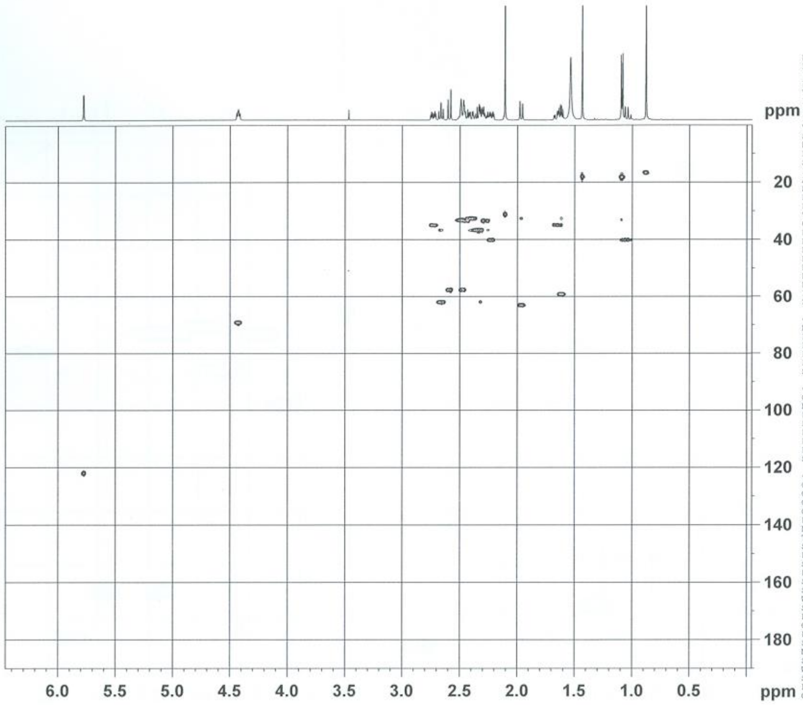


```
NAME            oct17-12
EXPNO                  8
PROCNO                 1
Date_           20121017
Time               18.46
INSTRUM            spect
PROBHD    5 mm BBI 1H/D-
PULPROG       hsqcetgpsi
TD                  1024
SOLVENT            CDCl3
NS                    32
DS                     8
SWH             3255.208 Hz
FIDRES          3.178914 Hz
AQ             0.1574900 sec
RG               23170.5
DW               153.600 usec
DE                  6.50 usec
TE                 298.0 K
CNST2        145.0000000
D0            0.00000300 sec
D1            1.50000000 sec
D4            0.00172414 sec
D11           0.03000000 sec
D13           0.00000400 sec
D16           0.00010000 sec
D24           0.00110000 sec
IN0           0.00002090 sec
ZGOPTNS

======== CHANNEL f1 ========
NUC1                  1H
P1                  7.60 usec
P2                 15.20 usec
P28                 0.00 usec
PL1                 6.00 dB
SFO1         500.1316254 MHz

======== CHANNEL f2 ========
CPDPRG2             garp
NUC2                 13C
P3                 13.70 usec
P4                 27.40 usec
PCPD2              65.00 usec
PL2                -2.85 dB
PL12               10.67 dB
SFO2         125.7697360 MHz

====== GRADIENT CHANNEL =====
GPNAM1          SINE.100
GPNAM2          SINE.100
GPZ1               80.00 %
GPZ2               20.10 %
P16              1000.00 usec
ND0                    2
TD                   256
SFO1            125.7697 MHz
FIDRES         93.344727 Hz
SW               190.000 ppm
FnMODE      Echo-Antiecho
SI                  1024
SF           500.1300233 MHz
WDW                QSINE
SSB                    2
LB                  0.00 Hz
GB                     0
PC                  4.00
SI                  1024
MC2         echo-antiecho
SF           125.7577898 MHz
WDW                QSINE
SSB                    2
LB                  0.00 Hz
GB                     0
```

AVANCE AV-500 LAB NO: 109-B

COMPOUND 4
COSY

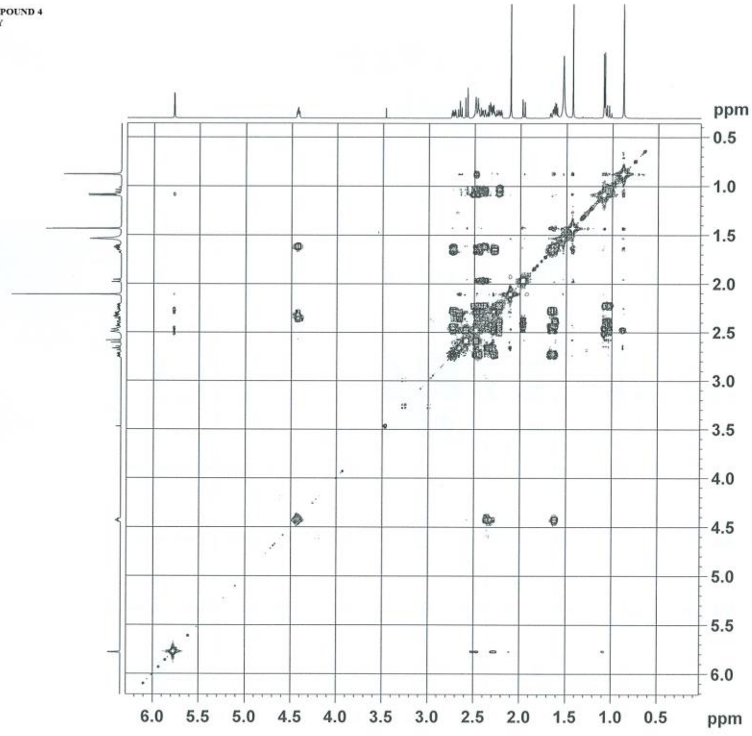


```
NAME            oct17-12
EXPNO                  6
PROCNO                 1
Date_           20121017
Time               14.41
INSTRUM            spect
PROBHD    5 mm BBI 1H/D-
PULPROG         cosygpqf
TD                  2048
SOLVENT            CDCl3
NS                     8
DS                     8
SWH             3255.208 Hz
FIDRES          1.589457 Hz
AQ             0.3147764 sec
RG                   362
DW               153.600 usec
DE                  6.50 usec
TE                 298.0 K
D0            0.00000300 sec
D1            1.50000000 sec
D13           0.00000400 sec
D16           0.00010000 sec
IN0           0.00030720 sec

======== CHANNEL f1 ========
NUC1                  1H
P0                  7.60 usec
P1                  7.60 usec
PL1                 6.00 dB
SFO1         500.1316254 MHz

====== GRADIENT CHANNEL =====
GPNAM1          SINE.100
GPZ1               10.00 %
P16              1000.00 usec
ND0                    1
TD                   256
SFO1            500.1316 MHz
FIDRES         12.715656 Hz
SW                 6.509 ppm
FnMODE                QF
SI                  1024
SF           500.1300233 MHz
WDW                 SINE
SSB                    0
LB                  0.00 Hz
GB                     0
PC                  4.00
SI                  1024
MC2                   QF
SF           500.1300233 MHz
WDW                 SINE
SSB                    0
LB                  0.00 Hz
GB                     0
```

AVANCE AV-500 LAB NO: 109-B

COMPOUND 4
HMBC

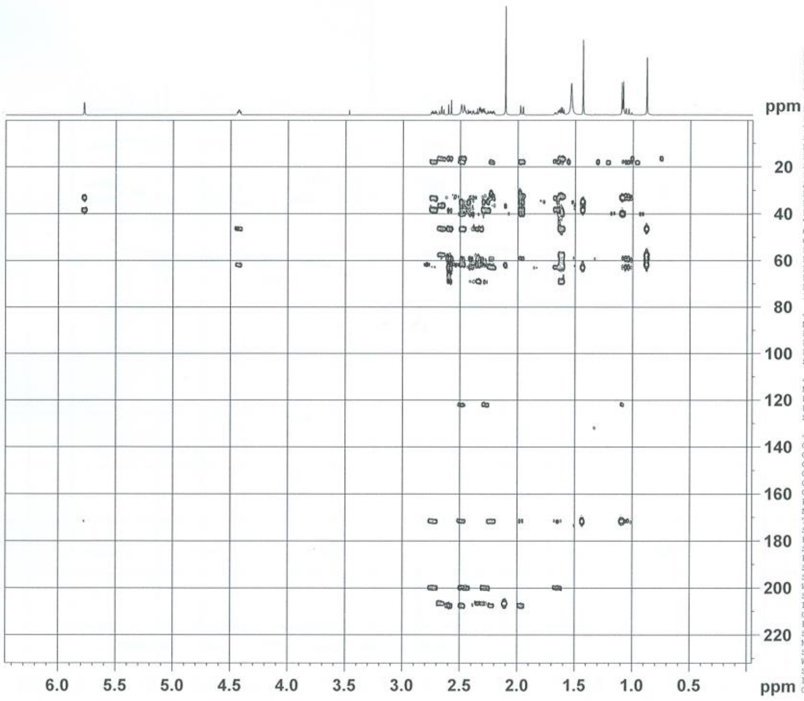


```
NAME          oct17-12
EXPNO                9
PROCNO               1
Date_         20121017
Time             22.37
INSTRUM          spect
PROBHD   5 mm BBI 1H/D-
PULPROG    hmbcgplpndqf
TD                4096
SOLVENT          CDCl3
NS                  64
DS                   8
SWH           3255.208 Hz
FIDRES        0.794729 Hz
AQ           0.6293492 sec
RG             23170.5
DW             153.600 usec
DE                6.50 usec
TE               298.0 K
CNST2      145.0000000
CNST13      10.0000000
D0          0.00000300 sec
D1          2.00000000 sec
D2          0.00344828 sec
D6          0.05000000 sec
D16         0.00010000 sec
IN0         0.00001715 sec

======== CHANNEL f1 ========
NUC1                1H
P1                7.60 usec
P2               15.20 usec
PL1               6.00 dB
SFO1       500.1316254 MHz

======== CHANNEL f2 ========
NUC2               13C
P3               13.70 usec
PL2              -2.85 dB
SFO2       125.7723769 MHz

====== GRADIENT CHANNEL =====
GPNAM1        SINE.100
GPNAM2        SINE.100
GPNAM3        SINE.100
GPZ1             50.00 %
GPZ2             30.00 %
GPZ3             40.10 %
P16            1000.00 usec
ND0                  2
TD                 205
SFO1          125.7724 MHz
FIDRES      142.337524 Hz
SW             232.000 ppm
FnMODE              QF
SI                1024
SF         500.1300233 MHz
WDW              QSINE
SSB                  0
LB                0.00 Hz
GB                   0
PC                4.00
SI                1024
MC2                 QF
SF         125.7577898 MHz
WDW              QSINE
SSB                  0
LB                0.00 Hz
GB                   0
```

AVANCE AV-500
LAB NO: 109-B

COMPOUND 4
NOESY

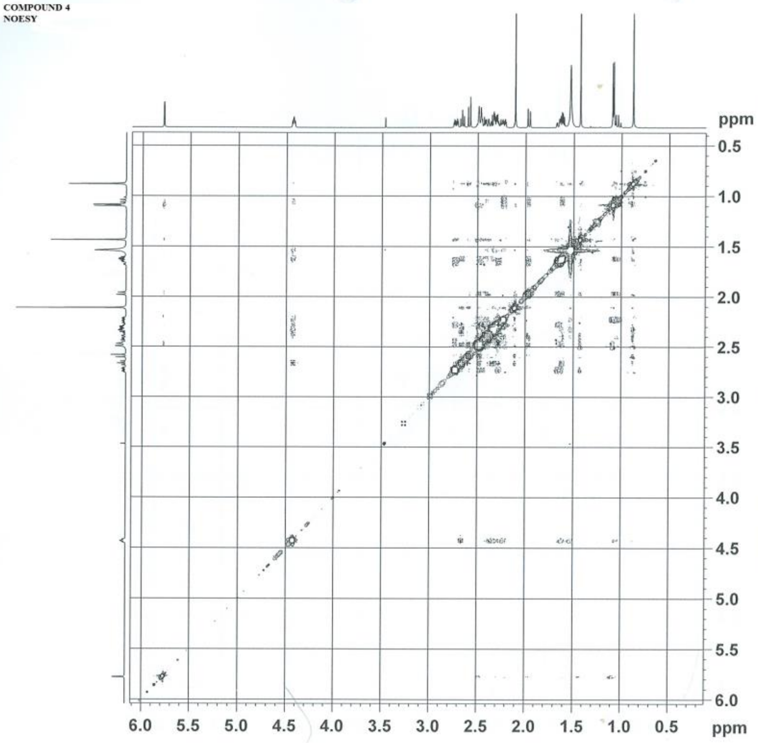


```
NAME          oct17-12
EXPNO                7
PROCNO               1
Date_         20121017
Time             15.45
INSTRUM          spect
PROBHD   5 mm BBI 1H/D-
PULPROG      noesygpph
TD                2048
SOLVENT          CDCl3
NS                  16
DS                   2
SWH           3255.208 Hz
FIDRES        1.589457 Hz
AQ           0.3147764 sec
RG              1625.5
DW             153.600 usec
DE                6.50 usec
TE               298.0 K
D0          0.00014392 sec
D1          1.50000000 sec
D8          0.80000001 sec
D16         0.00010000 sec
IN0         0.00030720 sec

======== CHANNEL f1 ========
NUC1                1H
P1                7.60 usec
P2               15.20 usec
PL1               6.00 dB
SFO1       500.1316254 MHz

====== GRADIENT CHANNEL =====
GPNAM1        SINE.100
GPZ1             40.00 %
P16            1000.00 usec
ND0                  1
TD                 256
SFO1          500.1316 MHz
FIDRES       12.715656 Hz
SW               6.509 ppm
FnMODE      States-TPPI
SI                1024
SF         500.1300233 MHz
WDW              QSINE
SSB                  2
LB                0.00 Hz
GB                   0
PC                4.00
SI                1024
MC2         States-TPPI
SF         500.1300233 MHz
WDW              QSINE
SSB                  2
LB                0.00 Hz
GB                   0
```

AVANCE AV-500
LAB NO: 109-B
