# Supplementary material for: New Anti-Inflammatory Metabolites by Microbial Transformation of Medrysone
Source: PLoS One. 2016 Apr 22;11(4):e0153951. doi: 10.1371/journal.pone.0153951 (PMC4841542; doi:10.1371/journal.pone.0153951)

COMPOUND 5

Date Run: 10-16-2012 (Time Run: 12:09:37)

Instrument: JEOL MSRoute
Inlet: Direct Probe

Run By: HEJ

Ionization mode: EI+

Scan: 14
Base: m/z 43; 7.5%FS TIC: 1393072

R.T.: 1.17

#Ions: 86

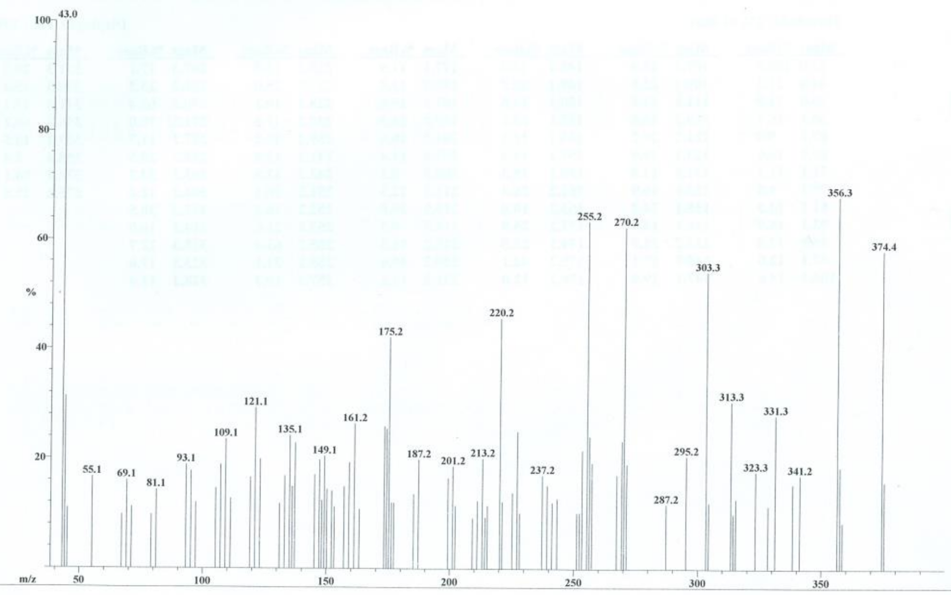

COMPOUND 5
HREI-MS

Mass	Relative Intensity	Theoretical Mass	Delta [mmu]	RDB	Composition
159.0822	2.9	159.0804	1.8	6.5	$C_{11} H_{11} O_1$
161.0978	4.6	161.0961	1.7	5.5	$C_{11} H_{13} O_1$
161.9895	7.3	161.9948	-5.3	8.0	$C_8 H_2 O_4$
168.9888	33.1	168.9920	-3.2	10.5	$C_{10} H_1 O_3$
173.1007	5.6	173.0961	4.6	6.5	$C_{12} H_{13} O_1$
174.1053	7.6	174.1039	1.4	6.0	$C_{12} H_{14} O_1$
175.1095	2.1	175.1117	-2.3	5.5	$C_{12} H_{15} O_1$
176.1090	1.5	176.1043	4.7	1.0	$C_8 H_{16} O_4$
180.9888	27.7	180.9920	-3.2	11.5	$C_{11} H_1 O_3$
185.9888	1.9	185.9948	-6.0	10.0	$C_{10} H_2 O_4$
187.1203	2.3				
192.9888	4.6	192.9920	-3.2	12.5	$C_{12} H_1 O_3$
199.1196	2.6				
204.9888	6.9	204.9920	-3.2	13.5	$C_{13} H_1 O_3$
213.1330	3.7	213.1274	5.6	7.5	$C_{15} H_{17} O_1$
218.9856	13.0				
225.1330	2.8	225.1274	5.6	8.5	$C_{16} H_{17} O_1$
230.9856	10.9				
242.9856	13.7				
254.9856	5.3				
255.1376	4.9	255.1380	-0.4	8.5	$C_{17} H_{19} O_2$
266.9775	2.2				
268.9824	3.7	268.9869	-4.5	17.5	$C_{17} H_1 O_4$
280.9824	10.2	280.9869	-4.5	18.5	$C_{18} H_1 O_4$
292.9824	17.4	292.9869	-4.5	19.5	$C_{19} H_1 O_4$
304.9824	4.5	304.9869	-4.5	20.5	$C_{20} H_1 O_4$
313.1557	4.4	313.1587	-3.0	13.5	$C_{23} H_{21} O_1$
316.9887	2.9	316.9869	1.8	21.5	$C_{21} H_1 O_4$
330.9792	4.3				
338.1831	4.6	338.1876	-4.5	10.0	$C_{22} H_{26} O_3$
354.9792	2.4				
356.2084	5.1	356.2135	-5.1	13.0	$C_{26} H_{28} O_1$
374.2068	4.0	374.2088	-1.9	8.0	$C_{22} H_{30} O_5$
		374.2029	3.9	17.0	$C_{29} H_{26}$
380.9760	5.0	380.9818	-5.8	25.5	$C_{25} H_1 O_5$
392.9760	3.0	392.9818	-5.8	26.5	$C_{26} H_1 O_5$
430.9434	3.6				
442.8956	2.7				

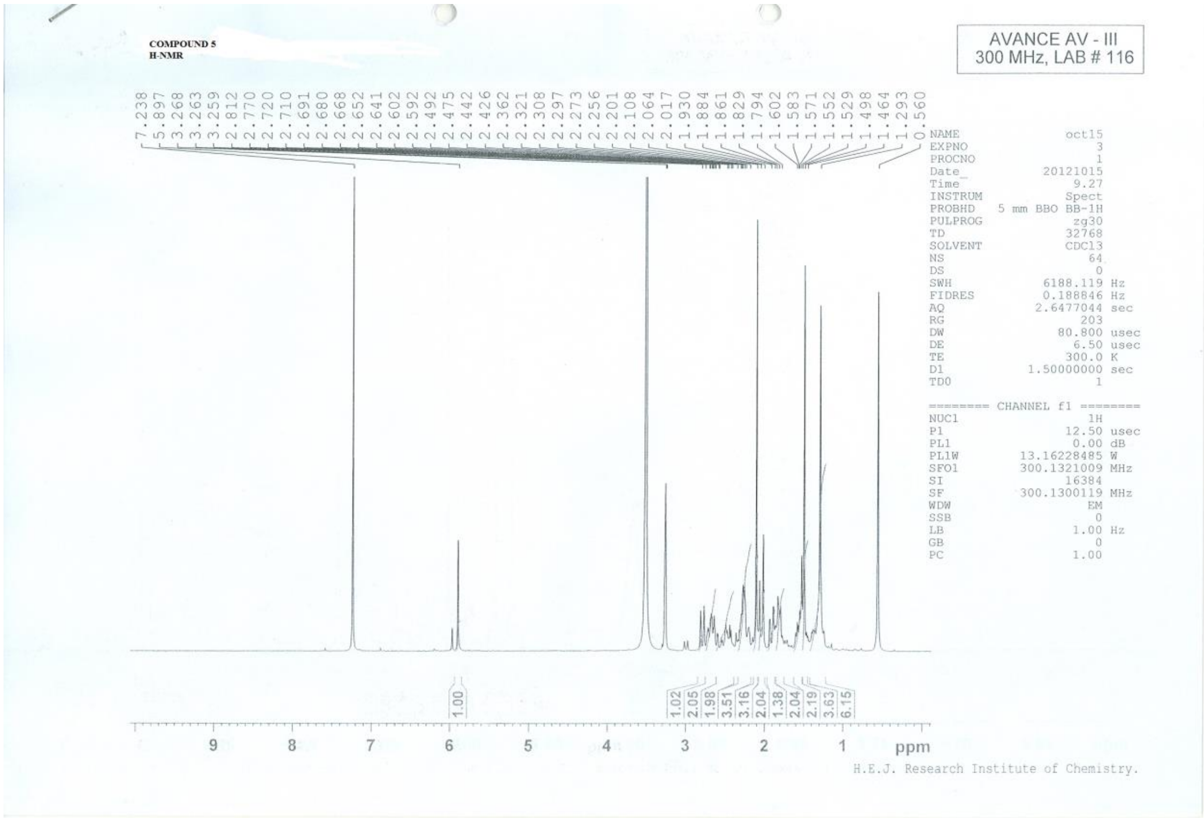
COMPOUND 5
H-NMR
AVANCE AV - III
300 MHz, LAB # 116
7.238 5.897 3.268 3.263 3.259 2.812 2.770 2.720 2.710 2.691 2.680 2.668 2.652 2.641 2.602 2.592 2.492 2.475 2.442 2.426 2.362 2.321 2.308 2.297 2.273 2.256 2.201 2.108 2.064 2.017 1.930 1.884 1.861 1.829 1.794 1.602 1.583 1.571 1.552 1.529 1.498 1.464 1.293 0.560
NAME oct15
EXPNO 3
PROCNO 1
Date_ 20121015
Time 9.27
INSTRUM Spect
PROBHD 5 mm BBO BB-1H
PULPROG zg30
TD 32768
SOLVENT CDCl3
NS 64
DS 0
SWH 6188.119 Hz
FIDRES 0.188846 Hz
AQ 2.6477044 sec
RG 203
DW 80.800 usec
DE 6.50 usec
TE 300.0 K
D1 1.50000000 sec
TD0 1
======== CHANNEL f1 ========
NUC1 1H
P1 12.50 usec
PL1 0.00 dB
PL1W 13.16228485 W
SFO1 300.1321009 MHz
SI 16384
SF 300.1300119 MHz
WDW EM
SSB 0
LB 1.00 Hz
GB 0
PC 1.00
1.00 1.02 2.05 1.98 3.51 3.16 2.04 1.38 2.04 2.19 3.63 6.15
9 8 7 6 5 4 3 2 1 ppm
H.E.J. Research Institute of Chemistry.

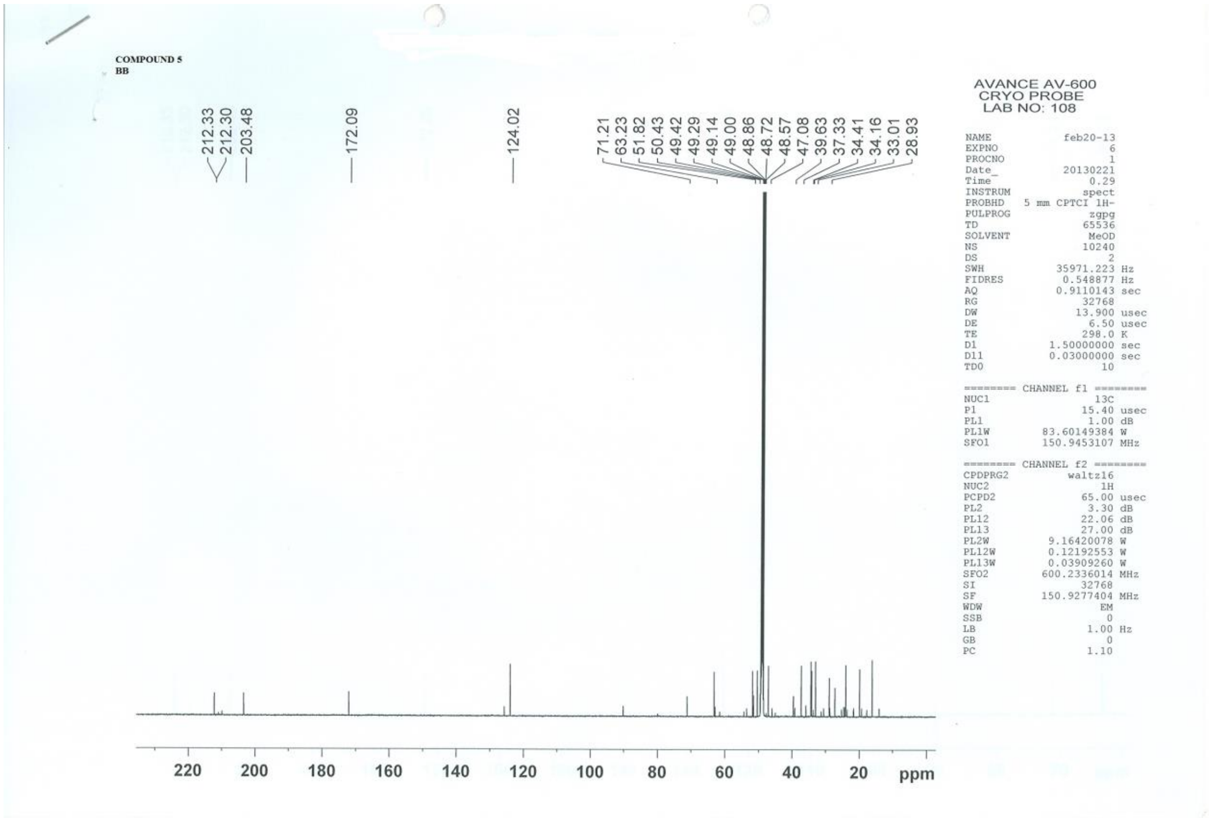
COMPOUND 5
BB
AVANCE AV-600
CRYO PROBE
LAB NO: 108
212.33 212.30 203.48 172.09 124.02 71.21 63.23 51.82 50.43 49.42 49.29 49.14 49.00 48.86 48.72 48.57 47.08 39.63 37.33 34.41 34.16 33.01 28.93
NAME feb20-13
EXPNO 6
PROCNO 1
Date_ 20130221
Time 0.29
INSTRUM spect
PROBHD 5 mm CPTCI 1H-
PULPROG zgpg
TD 65536
SOLVENT MeOD
NS 10240
DS 2
SWH 35971.223 Hz
FIDRES 0.548877 Hz
AQ 0.9110143 sec
RG 32768
DW 13.900 usec
DE 6.50 usec
TE 298.0 K
D1 1.50000000 sec
D11 0.03000000 sec
TD0 10
======== CHANNEL f1 ========
NUC1 13C
P1 15.40 usec
PL1 1.00 dB
PL1W 83.60149384 W
SFO1 150.9453107 MHz
======== CHANNEL f2 ========
CPDPRG2 waltz16
NUC2 1H
PCPD2 65.00 usec
PL2 3.30 dB
PL12 22.06 dB
PL13 27.00 dB
PL2W 9.16420078 W
PL12W 0.12192553 W
PL13W 0.03909260 W
SFO2 600.2336014 MHz
SI 32768
SF 150.9277404 MHz
WDW EM
SSB 0
LB 1.00 Hz
GB 0
PC 1.10
220 200 180 160 140 120 100 80 60 40 20 ppm

COMPOUND 5
DEPT-135

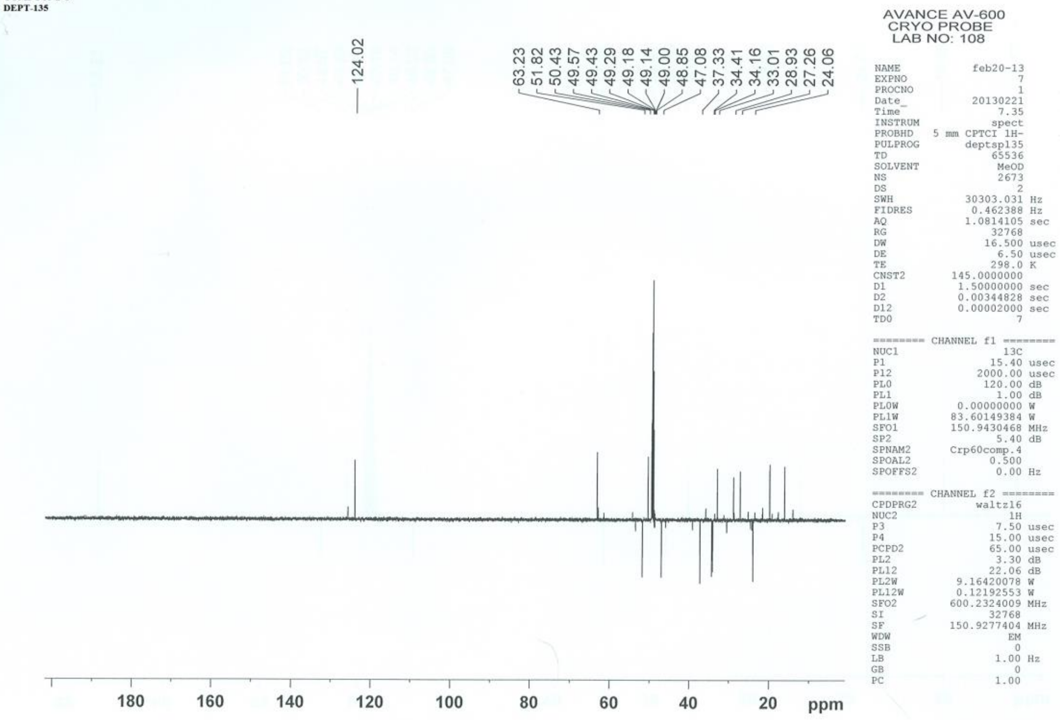

COMPOUND 5
DEPT-90

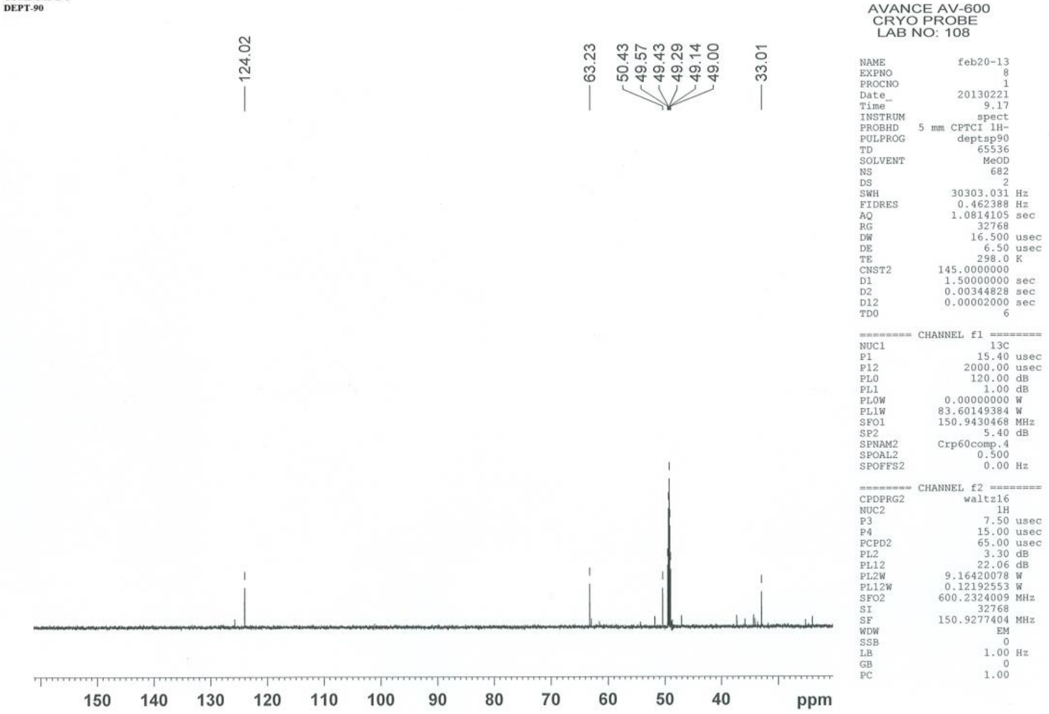

COMPOUND 5
HSQC

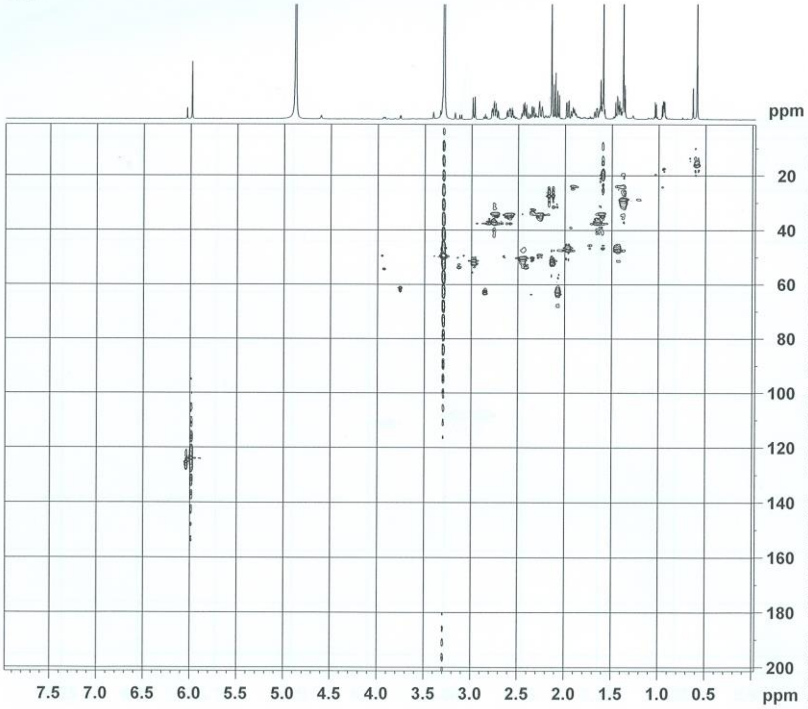

AVANCE AV-600
CRYO PROBE
LAB NO: 108

NAME	feb20-13
EXPNO	4
PROCNO	1
Date_	20130220
Time	15.34
INSTRUM	spect
PROBHD	5 mm CPTCI 1H-
PULPROG	hsqcetgpsi
TD	1024
SOLVENT	MeOD
NS	32
DS	8
SWH	4807.692 Hz
FIDRES	4.695012 Hz
AQ	0.1066500 sec
RG	36780.8
DW	104.000 usec
DE	6.50 usec
TE	298.0 K
CNST2	145.0000000
D0	0.00000300 sec
D1	1.50000000 sec
D4	0.00172414 sec
D11	0.03000000 sec
D13	0.00000400 sec
D16	0.00015000 sec
D24	0.00110000 sec
IN0	0.00001655 sec
ZGOPTNS	

======== CHANNEL f1 ========

NUC1	1H
P1	7.20 usec
P2	14.40 usec
P28	0.50 usec
PL1	3.30 dB
PL1W	9.16420078 W
SFO1	600.2324009 MHz

======== CHANNEL f2 ========

CPDPRG2	garp
NUC2	13C
P3	15.40 usec
P4	30.80 usec
PCPD2	61.00 usec
PL2	1.00 dB
PL12	13.00 dB
PL2W	83.60149384 W
PL12W	5.27489758 W
SFO2	150.9430468 MHz

====== GRADIENT CHANNEL =====

GPNAM1	SINE.100
GPNAM2	SINE.100
GPZ1	80.00 %
GPZ2	20.10 %
P16	2000.00 usec
ND0	2

COMPOUND 5
COSY

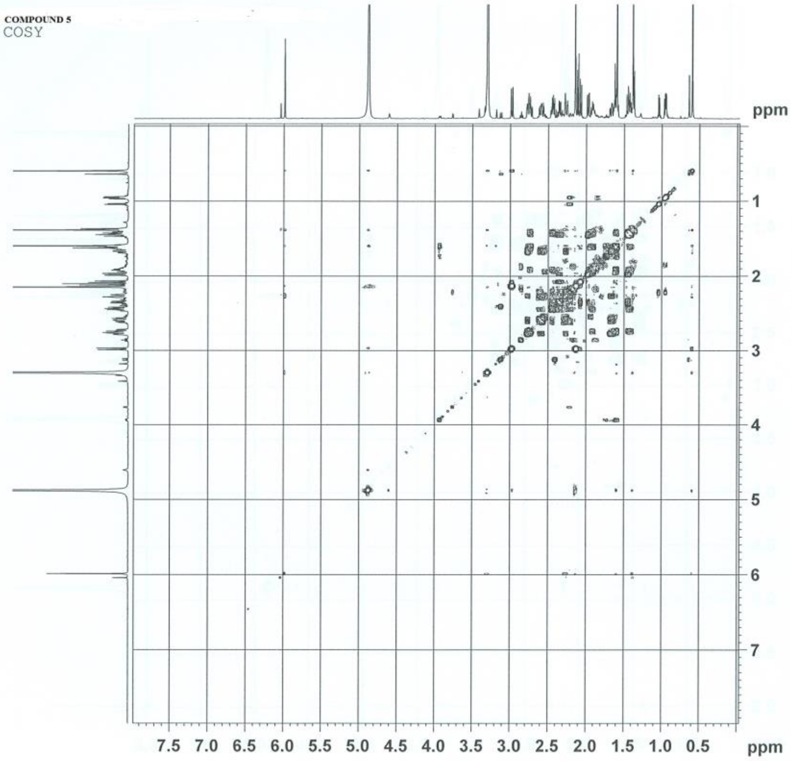

AVANCE AV-600
CRYO PROBE
LAB NO: 108

NAME	feb20-13
EXPNO	2
PROCNO	1
Date_	20130220
Time	13.10
INSTRUM	spect
PROBHD	5 mm CPTCI 1H-
PULPROG	cosydfqf
TD	2048
SOLVENT	MeOD
NS	8
DS	4
SWH	4807.692 Hz
FIDRES	2.347506 Hz
AQ	0.2131460 sec
RG	35.9
DW	104.000 usec
DE	6.50 usec
TE	298.0 K
D0	0.00000300 sec
D1	1.50000000 sec
D13	0.00000400 sec
D20	0.00000200 sec
IN0	0.00020800 sec

======== CHANNEL f1 ========

NUC1	1H
P1	7.20 usec
PL1	3.30 dB
PL1W	9.16420078 W
SFO1	600.2324009 MHz
ND0	1
TD	256
SFO1	600.2324 MHz
FIDRES	18.780046 Hz
SW	8.010 ppm
FnMODE	QF
SI	1024
SF	600.2300156 MHz
WDW	QSINE
SSB	0
LB	0.00 Hz
GB	0
PC	1.40
SI	1024
MC2	QF
SF	600.2300156 MHz
WDW	QSINE
SSB	0
LB	0.00 Hz
GB	0

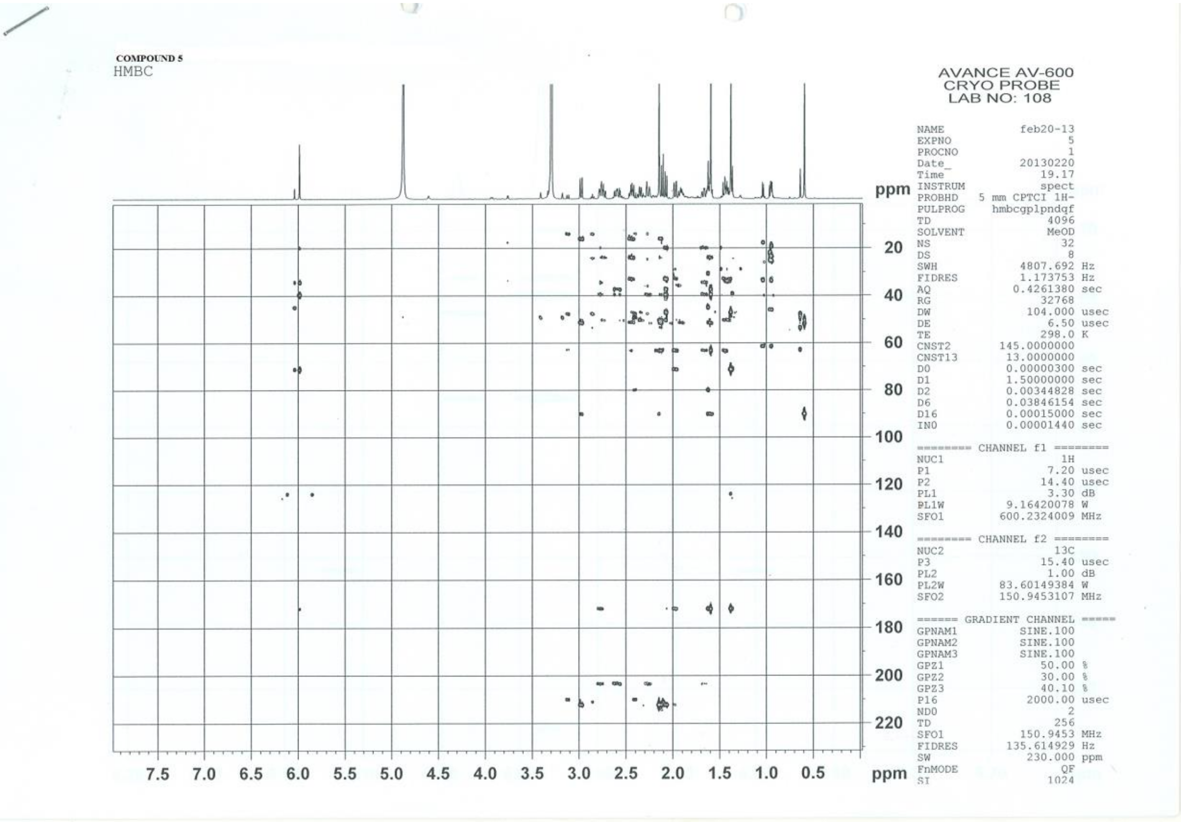
COMPOUND 5
HMBC
AVANCE AV-600
CRYO PROBE
LAB NO: 108
NAME feb20-13
EXPNO 5
PROCNO 1
Date_ 20130220
Time 19.17
INSTRUM spect
PROBHD 5 mm CPTCI 1H-
PULPROG hmbcgplpndqf
TD 4096
SOLVENT MeOD
NS 32
DS 8
SWH 4807.692 Hz
FIDRES 1.173753 Hz
AQ 0.4261380 sec
RG 32768
DW 104.000 usec
DE 6.50 usec
TE 298.0 K
CNST2 145.0000000
CNST13 13.0000000
D0 0.00000300 sec
D1 1.50000000 sec
D2 0.00344828 sec
D6 0.03846154 sec
D16 0.00015000 sec
IN0 0.00001440 sec
======== CHANNEL f1 ========
NUC1 1H
P1 7.20 usec
P2 14.40 usec
PL1 3.30 dB
PL1W 9.16420078 W
SFO1 600.2324009 MHz
======== CHANNEL f2 ========
NUC2 13C
P3 15.40 usec
PL2 1.00 dB
PL2W 83.60149384 W
SFO2 150.9453107 MHz
====== GRADIENT CHANNEL =====
GPNAM1 SINE.100
GPNAM2 SINE.100
GPNAM3 SINE.100
GPZ1 50.00 %
GPZ2 30.00 %
GPZ3 40.10 %
P16 2000.00 usec
ND0 2
TD 256
SFO1 150.9453 MHz
FIDRES 135.614929 Hz
SW 230.000 ppm
FnMODE QF
SI 1024
ppm
ppm
7.5 7.0 6.5 6.0 5.5 5.0 4.5 4.0 3.5 3.0 2.5 2.0 1.5 1.0 0.5
20 40 60 80 100 120 140 160 180 200 220

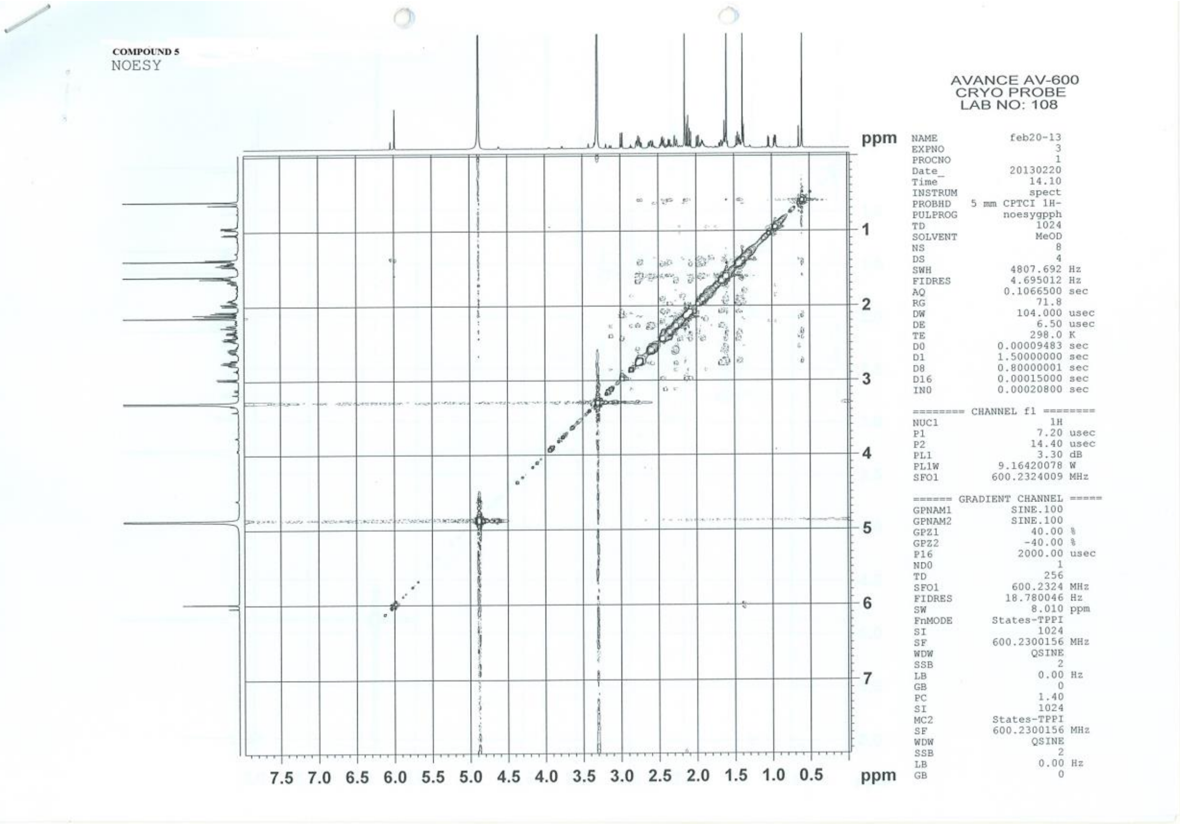
COMPOUND 5
NOESY
AVANCE AV-600
CRYO PROBE
LAB NO: 108
NAME feb20-13
EXPNO 3
PROCNO 1
Date_ 20130220
Time 14.10
INSTRUM spect
PROBHD 5 mm CPTCI 1H-
PULPROG noesygpph
TD 1024
SOLVENT MeOD
NS 8
DS 4
SWH 4807.692 Hz
FIDRES 4.695012 Hz
AQ 0.1066500 sec
RG 71.8
DW 104.000 usec
DE 6.50 usec
TE 298.0 K
D0 0.00009483 sec
D1 1.50000000 sec
D8 0.80000001 sec
D16 0.00015000 sec
IN0 0.00020800 sec
======== CHANNEL f1 ========
NUC1 1H
P1 7.20 usec
P2 14.40 usec
PL1 3.30 dB
PL1W 9.16420078 W
SFO1 600.2324009 MHz
====== GRADIENT CHANNEL =====
GPNAM1 SINE.100
GPNAM2 SINE.100
GPZ1 40.00 %
GPZ2 -40.00 %
P16 2000.00 usec
ND0 1
TD 256
SFO1 600.2324 MHz
FIDRES 18.780046 Hz
SW 8.010 ppm
FnMODE States-TPPI
SI 1024
SF 600.2300156 MHz
WDW QSINE
SSB 2
LB 0.00 Hz
GB 0
PC 1.40
SI 1024
MC2 States-TPPI
SF 600.2300156 MHz
WDW QSINE
SSB 2
LB 0.00 Hz
GB 0
ppm
ppm
7.5 7.0 6.5 6.0 5.5 5.0 4.5 4.0 3.5 3.0 2.5 2.0 1.5 1.0 0.5
1 2 3 4 5 6 7

COMPOUND 5
NOESY

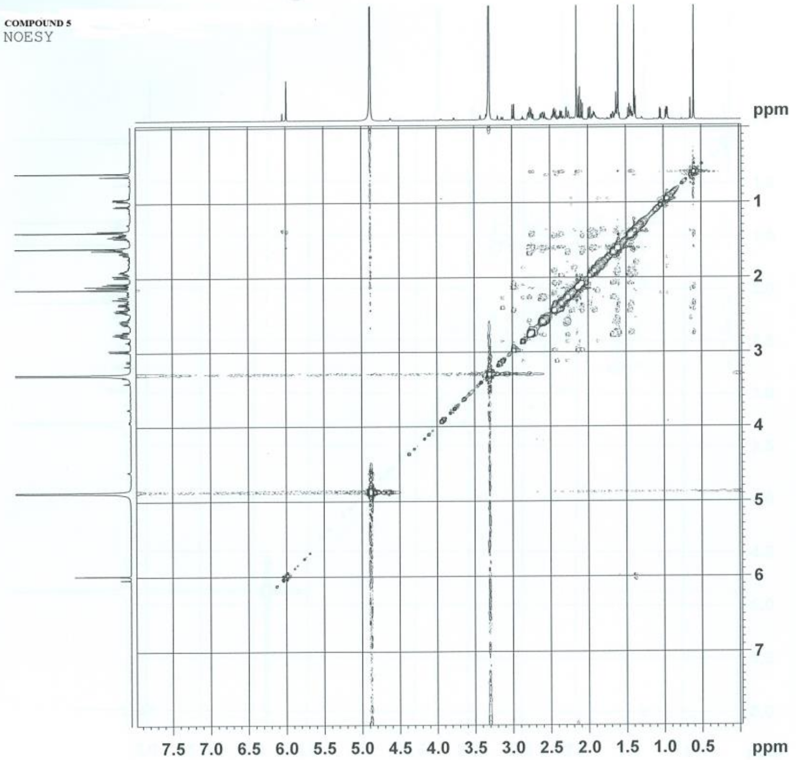

AVANCE AV-600
CRYO PROBE
LAB NO: 108

```
NAME              feb20-13
EXPNO                    3
PROCNO                   1
Date_             20130220
Time                 14.10
INSTRUM              spect
PROBHD     5 mm CPTCI 1H-
PULPROG          noesygpph
TD                    1024
SOLVENT               MeOD
NS                       8
DS                       4
SWH               4807.692 Hz
FIDRES            4.695012 Hz
AQ               0.1066500 sec
RG                    71.8
DW                 104.000 usec
DE                    6.50 usec
TE                   298.0 K
D0              0.00009483 sec
D1              1.50000000 sec
D8              0.80000001 sec
D16             0.00015000 sec
IN0             0.00020800 sec

======== CHANNEL f1 ========
NUC1                    1H
P1                    7.20 usec
P2                   14.40 usec
PL1                   3.30 dB
PL1W            9.16420078 W
SFO1          600.2324009 MHz

====== GRADIENT CHANNEL =====
GPNAM1            SINE.100
GPNAM2            SINE.100
GPZ1                 40.00 %
GPZ2                -40.00 %
P16                2000.00 usec
ND0                      1
TD                     256
SFO1              600.2324 MHz
FIDRES           18.780046 Hz
SW                   8.010 ppm
FnMODE         States-TPPI
SI                    1024
SF             600.2300156 MHz
WDW                  QSINE
SSB                      2
LB                    0.00 Hz
GB                       0
PC                    1.40
SI                    1024
MC2            States-TPPI
SF             600.2300156 MHz
WDW                  QSINE
SSB                      2
LB                    0.00 Hz
GB                       0
```

COMPOUND 5
NOESY

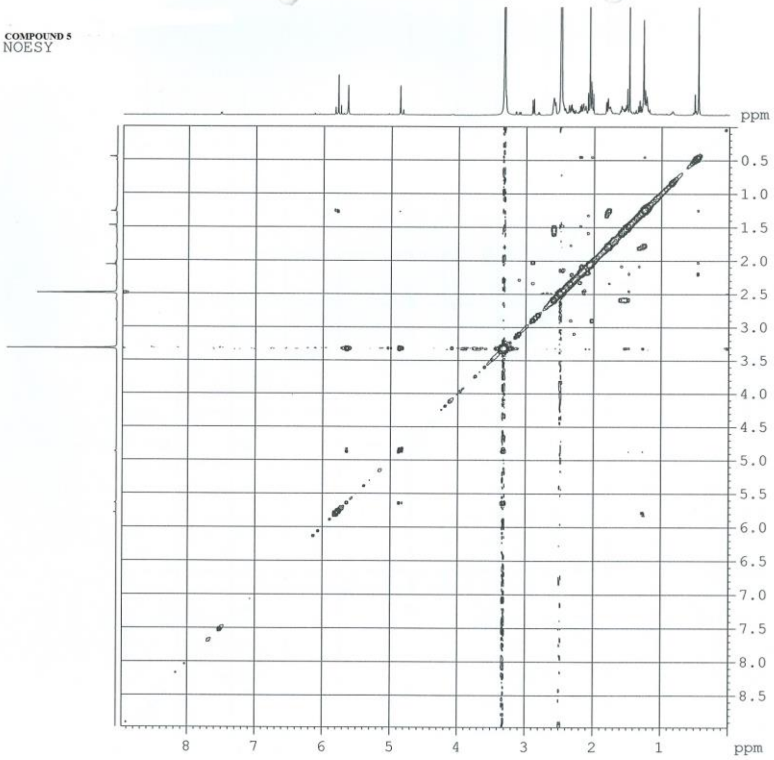

AVANCE AV-600
CRYO PROBE
LAB NO: 108

NAME	feb24-15	
EXPNO	27	
PROCNO	1	
Date_	20150224	
Time	17.22	
INSTRUM	spect	
PROBHD	5 mm CPTCI 1H-	
PULPROG	noesygpph	
TD	1024	
SOLVENT	DMSO	
NS	16	
DS	4	
SWH	5387.931	Hz
FIDRES	5.261652	Hz
AQ	0.0951700	sec
RG	71.8	
DW	92.800	usec
DE	6.50	usec
TE	298.0	K
D0	0.00008338	sec
D1	2.00000000	sec
D8	0.80000001	sec
D16	0.00015000	sec
IN0	0.00018560	sec
======== CHANNEL f1 ========		
NUC1	1H	
P1	7.40	usec
P2	14.80	usec
PL1	3.30	dB
PL1W	9.16420078	W
SFO1	600.2327010	MHz
====== GRADIENT CHANNEL =====		
GPNAM1	SINE.100	
GPNAM2	SINE.100	
GPZ1	40.00	%
GPZ2	-40.00	%
P16	2000.00	usec
ND0	1	
TD	256	
SFO1	600.2327	MHz
FIDRES	21.046606	Hz
SW	8.976	ppm
FnMODE	States-TPPI	
SI	1024	
SF	600.2300088	MHz
WDW	QSINE	
SSB	2	
LB	0.00	Hz
GB	0	
PC	1.40	
SI	1024	
MC2	States-TPPI	
SF	600.2300088	MHz
WDW	QSINE	
SSB	2	
LB	0.00	Hz
GB	0	

Compound 5
NOESY **(DMSO)**

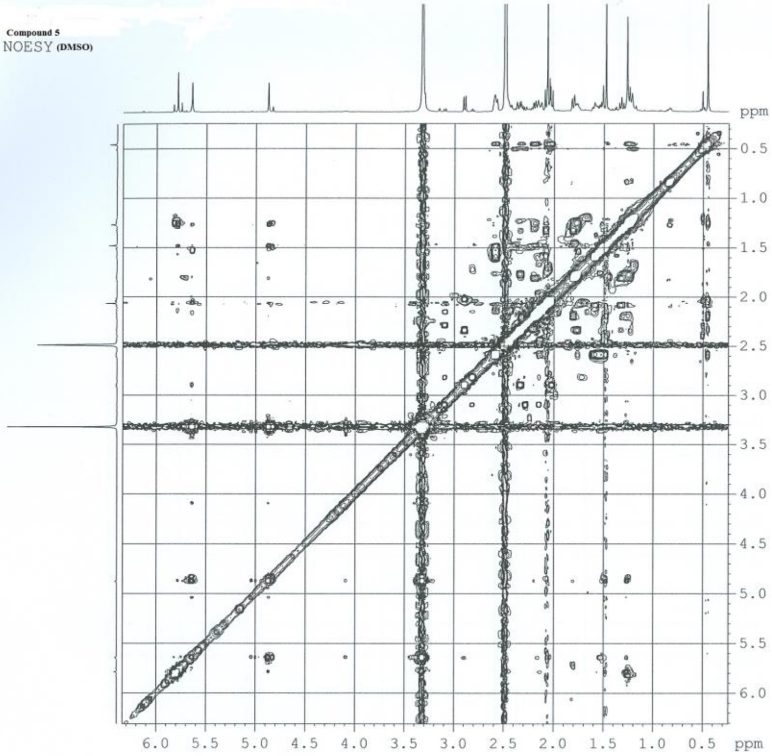

Supplement: S5 File — (PDF) [file pone.0153951.s005.pdf]
