# Supplementary material for: New Anti-Inflammatory Metabolites by Microbial Transformation of Medrysone
Source: PLoS One. 2016 Apr 22;11(4):e0153951. doi: 10.1371/journal.pone.0153951 (PMC4841542; doi:10.1371/journal.pone.0153951)
Supplement: S6 File — (PDF) [file pone.0153951.s006.pdf]

COMPOUND 6
ESI-MS

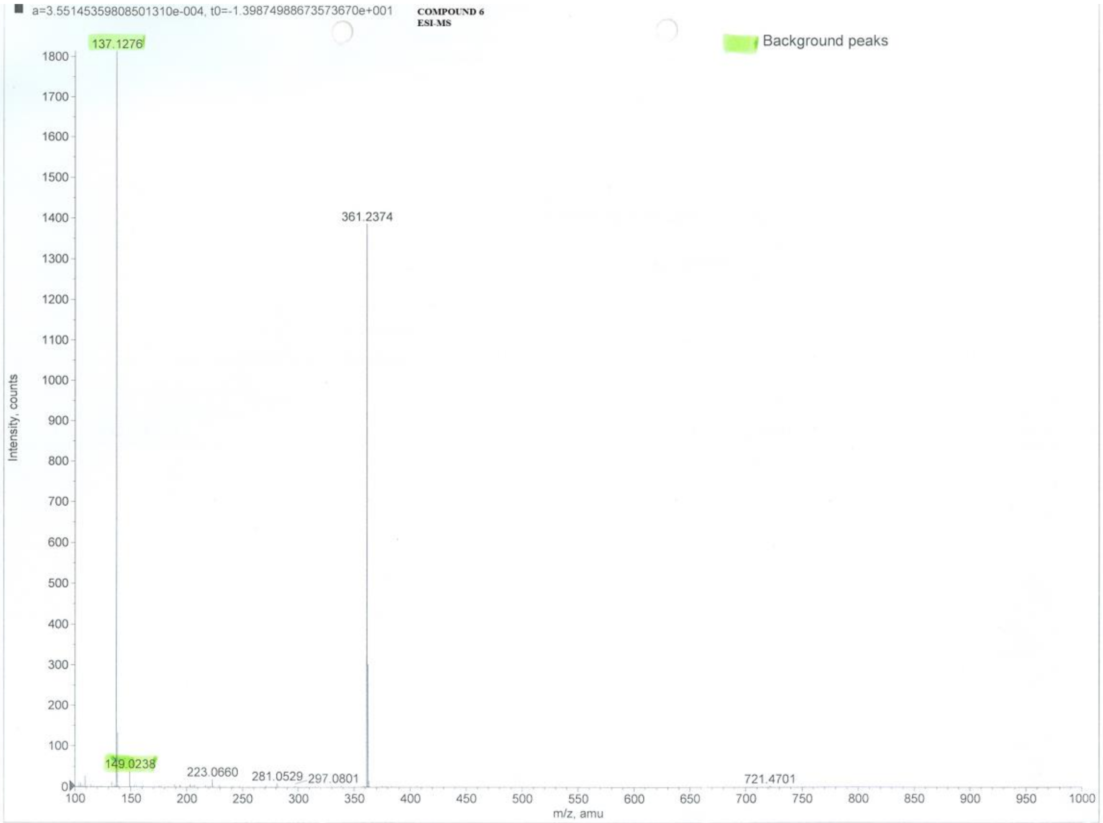

COMPOUND 6
HRESI-MS

Target m/z: +361.2382 amu
Tolerance: +10.0000 ppm
Result type: Elemental
Max num of results: 100
Min DBE: -0.5000 Max DBE: +50.0000
Electron state: OddAndEven
Num of charges: 0
Add water: N/A
Add proton: N/A
File Name: MEDCB-4a 25-2-13.wiff

	Elements	Min Number	Max Number
1	C	0	30
2	H	0	50
3	O	0	4

	Formula	Calculated m/z (amu)	mDa Error	PPM Error	DBE
1	C22 H33 O4	361.2378	0.3151	0.8723	6.5

COMPOUND 6

1H

AVANCE AV-600
CRYO PROBE
LAB NO: 108

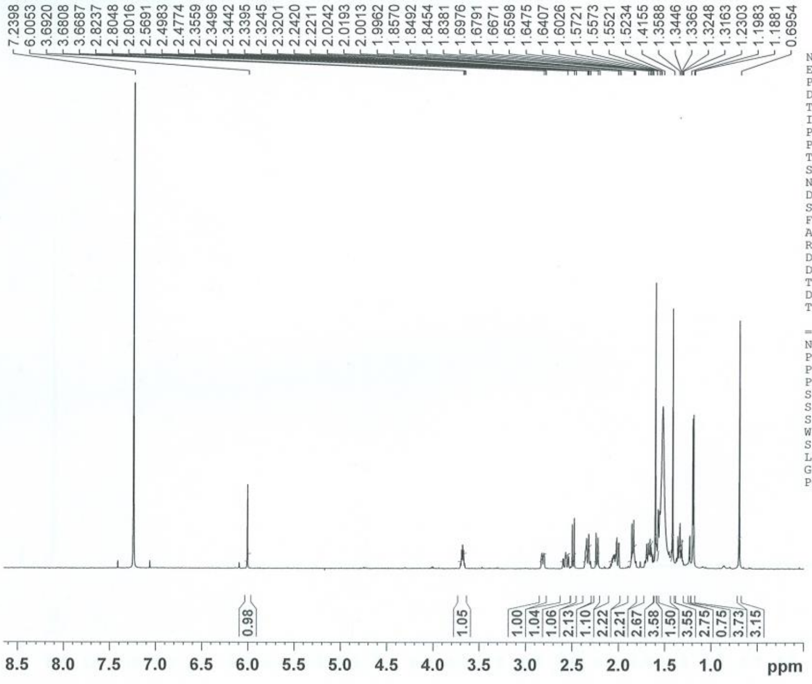

NAME	dec-16-12
EXPNO	1
PROCNO	1
Date_	20121216
Time	10.32
INSTRUM	spect
PROBHD	5 mm CPTCI 1H-
PULPROG	zg30
TD	32768
SOLVENT	CDCl3
NS	16
DS	0
SWH	12019.230 Hz
FIDRES	0.366798 Hz
AQ	1.3632404 sec
RG	9
DW	41.600 usec
DE	6.50 usec
TE	298.0 K
D1	2.00000000 sec
TD0	1

======== CHANNEL f1 ========

NUC1	1H
P1	7.20 usec
PL1	3.30 dB
PL1W	9.16420078 W
SFO1	600.2354021 MHz
SI	16384
SF	600.2300251 MHz
WDW	EM
SSB	0
LB	0.30 Hz
GB	0
PC	0.50

COMPOUND 6
BB

AVANCE AV-600
CRYO PROBE
LAB NO: 108

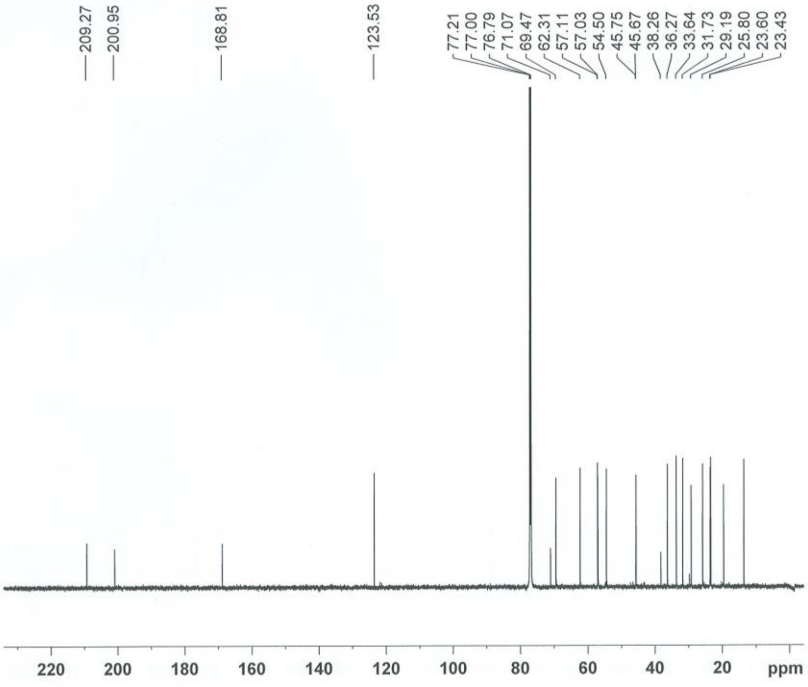

NAME	dec-16-12
EXPNO	6
PROCNO	1
Date_	20121216
Time	21.47
INSTRUM	spect
PROBHD	5 mm CPTCI 1H-
PULPROG	zgpg
TD	65536
SOLVENT	CDCl3
NS	12288
DS	2
SWH	35971.223 Hz
FIDRES	0.548877 Hz
AQ	0.9110143 sec
RG	32768
DW	13.900 usec
DE	6.50 usec
TE	294.5 K
D1	1.50000000 sec
D11	0.03000000 sec
TD0	12

======== CHANNEL f1 ========

NUC1	13C
P1	15.40 usec
PL1	1.00 dB
PL1W	83.60149384 W
SFO1	150.9453107 MHz

======== CHANNEL f2 ========

CPDPRG2	waltz16
NUC2	1H
PCPD2	65.00 usec
PL2	3.30 dB
PL12	22.06 dB
PL13	27.00 dB
PL2W	9.16420078 W
PL12W	0.12192553 W
PL13W	0.03909260 W
SFO2	600.2336014 MHz
SI	32768
SF	150.9279594 MHz
WDW	EM
SSB	0
LB	1.00 Hz
GB	0
PC	1.10

COMPOUND 6
DEPT-135

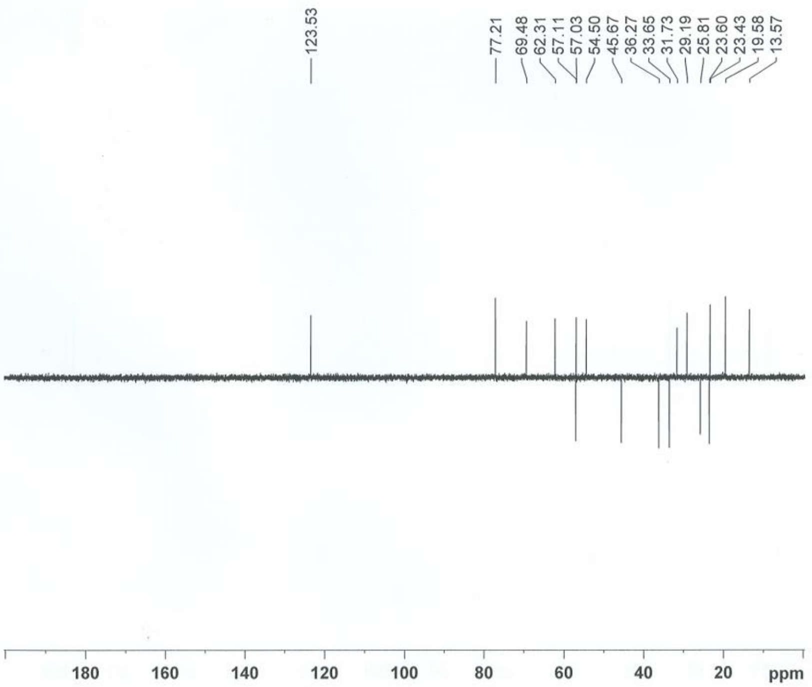

AVANCE AV-600
CRYO PROBE
LAB NO: 108

```
NAME            dec-16-12
EXPNO                   7
PROCNO                  1
Date_            20121217
Time                 6.18
INSTRUM             spect
PROBHD    5 mm CPTCI 1H-
PULPROG        deptsp135
TD                  65536
SOLVENT             CDCl3
NS                   2486
DS                      2
SWH             30303.031 Hz
FIDRES           0.462388 Hz
AQ              1.0814105 sec
RG                  32768
DW                 16.500 usec
DE                   6.50 usec
TE                  294.3 K
CNST2         145.0000000
D1             1.50000000 sec
D2             0.00344828 sec
D12            0.00002000 sec
TD0                     6

======== CHANNEL f1 ========
NUC1                  13C
P1                  15.40 usec
P12               2000.00 usec
PL0                120.00 dB
PL1                  1.00 dB
PL0W           0.00000000 W
PL1W          83.60149384 W
SFO1          150.9430468 MHz
SP2                  5.40 dB
SPNAM2        Crp60comp.4
SPOAL2              0.500
SPOFFS2              0.00 Hz

======== CHANNEL f2 ========
CPDPRG2           waltz16
NUC2                   1H
P3                   7.50 usec
P4                  15.00 usec
PCPD2               65.00 usec
PL2                  3.30 dB
PL12                22.06 dB
PL2W           9.16420078 W
PL12W          0.12192553 W
SFO2          600.2324009 MHz
SI                  32768
SF            150.9279594 MHz
WDW                    EM
SSB                     0
LB                   1.00 Hz
GB                      0
PC                   1.00
```

COMPOUND 6
DEPT-90

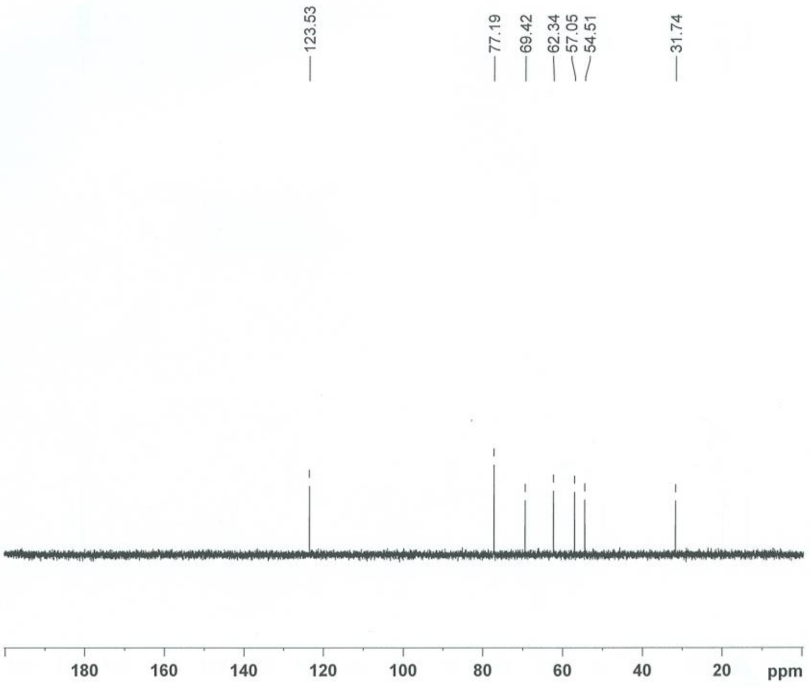

AVANCE AV-600
CRYO PROBE
LAB NO: 108

```
NAME            dec-16-12
EXPNO                   8
PROCNO                  1
Date_            20121217
Time                 8.00
INSTRUM             spect
PROBHD    5 mm CPTCI 1H-
PULPROG         deptsp90
TD                  65536
SOLVENT             CDCl3
NS                    864
DS                      2
SWH             30303.031 Hz
FIDRES           0.462388 Hz
AQ              1.0814105 sec
RG                  32768
DW                 16.500 usec
DE                   6.50 usec
TE                  298.0 K
CNST2         145.0000000
D1             1.50000000 sec
D2             0.00344828 sec
D12            0.00002000 sec
TD0                     4

======== CHANNEL f1 ========
NUC1                  13C
P1                  15.40 usec
P12               2000.00 usec
PL0                120.00 dB
PL1                  1.00 dB
PL0W           0.00000000 W
PL1W          83.60149384 W
SFO1          150.9430468 MHz
SP2                  5.40 dB
SPNAM2        Crp60comp.4
SPOAL2              0.500
SPOFFS2              0.00 Hz

======== CHANNEL f2 ========
CPDPRG2           waltz16
NUC2                   1H
P3                   7.50 usec
P4                  15.00 usec
PCPD2               65.00 usec
PL2                  3.30 dB
PL12                22.06 dB
PL2W           9.16420078 W
PL12W          0.12192553 W
SFO2          600.2324009 MHz
SI                  32768
SF            150.9279594 MHz
WDW                    EM
SSB                     0
LB                   1.00 Hz
GB                      0
PC                   1.00
```

COMPOUND 6
HSQC

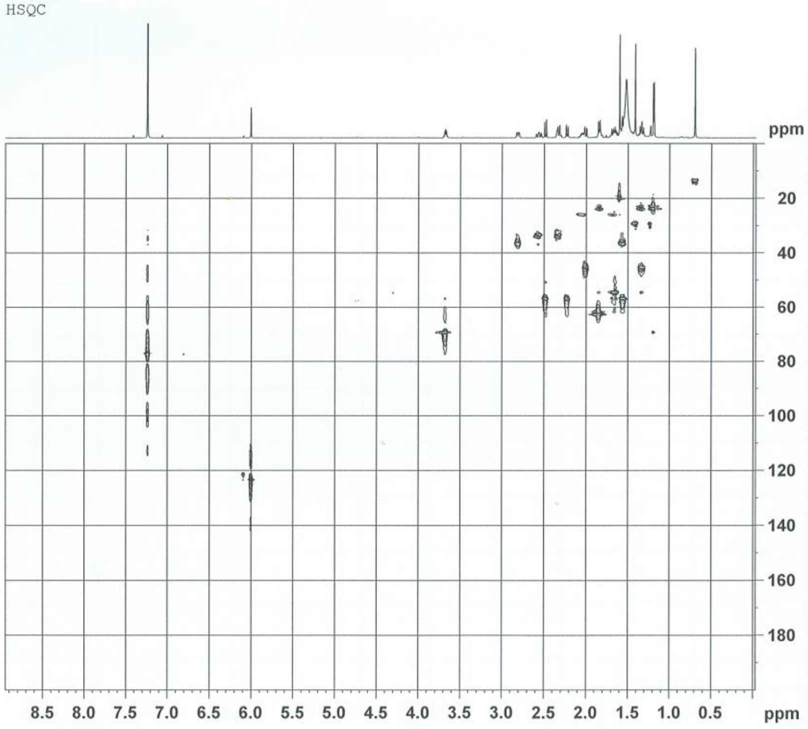

AVANCE AV-600
CRYO PROBE
LAB NO: 108

NAME	dec-16-12
EXPNO	4
PROCNO	1
Date_	20121216
Time	13.00
INSTRUM	spect
PROBHD	5 mm CPTCI 1H-
PULPROG	hsqcetgpsi
TD	1024
SOLVENT	CDCl3
NS	32
DS	8
SWH	5387.931 Hz
FIDRES	5.261652 Hz
AQ	0.0951700 sec
RG	46341
DW	92.800 usec
DE	6.50 usec
TE	298.0 K
CNST2	145.0000000
D0	0.00000300 sec
D1	1.50000000 sec
D4	0.00172414 sec
D11	0.03000000 sec
D13	0.00000400 sec
D16	0.00015000 sec
D24	0.00110000 sec
IN0	0.00001655 sec
ZGOPTNS	

======== CHANNEL f1 ========

NUC1	1H
P1	7.20 usec
P2	14.40 usec
P28	0.50 usec
PL1	3.30 dB
PL1W	9.16420078 W
SFO1	600.2327010 MHz

======== CHANNEL f2 ========

CPDPRG2	garp
NUC2	13C
P3	15.40 usec
P4	30.80 usec
PCPD2	61.00 usec
PL2	1.00 dB
PL12	13.00 dB
PL2W	83.60149384 W
PL12W	5.27489758 W
SFO2	150.9430468 MHz

====== GRADIENT CHANNEL =====

GPNAM1	SINE.100
GPNAM2	SINE.100
GPZ1	80.00 %
GPZ2	20.10 %
P16	2000.00 usec
ND0	2

COMPOUND 6
COSY

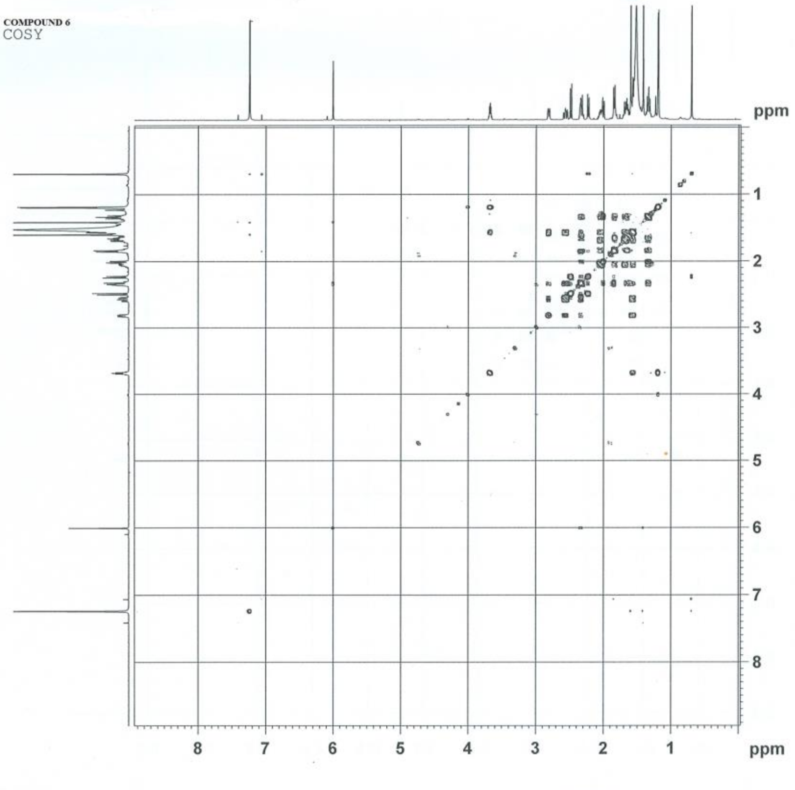

AVANCE AV-600
CRYO PROBE
LAB NO: 108

NAME	dec-16-12
EXPNO	2
PROCNO	1
Date_	20121216
Time	10.38
INSTRUM	spect
PROBHD	5 mm CPTCI 1H-
PULPROG	cosydfqf
TD	2048
SOLVENT	CDCl3
NS	8
DS	4
SWH	5387.931 Hz
FIDRES	2.630826 Hz
AQ	0.1901972 sec
RG	35.9
DW	92.800 usec
DE	6.50 usec
TE	298.0 K
D0	0.00000300 sec
D1	1.50000000 sec
D13	0.00000400 sec
D20	0.00000200 sec
IN0	0.00018560 sec

======== CHANNEL f1 ========

NUC1	1H
P1	7.20 usec
PL1	3.30 dB
PL1W	9.16420078 W
SFO1	600.2327010 MHz
ND0	1
TD	256
SFO1	600.2327 MHz
FIDRES	21.046606 Hz
SW	8.976 ppm
FnMODE	QF
SI	1024
SF	600.2300251 MHz
WDW	QSINE
SSB	0
LB	0.00 Hz
GB	0
PC	1.40
SI	1024
MC2	QF
SF	600.2300251 MHz
WDW	QSINE
SSB	0
LB	0.00 Hz
GB	0

COMPOUND 6
HMBC

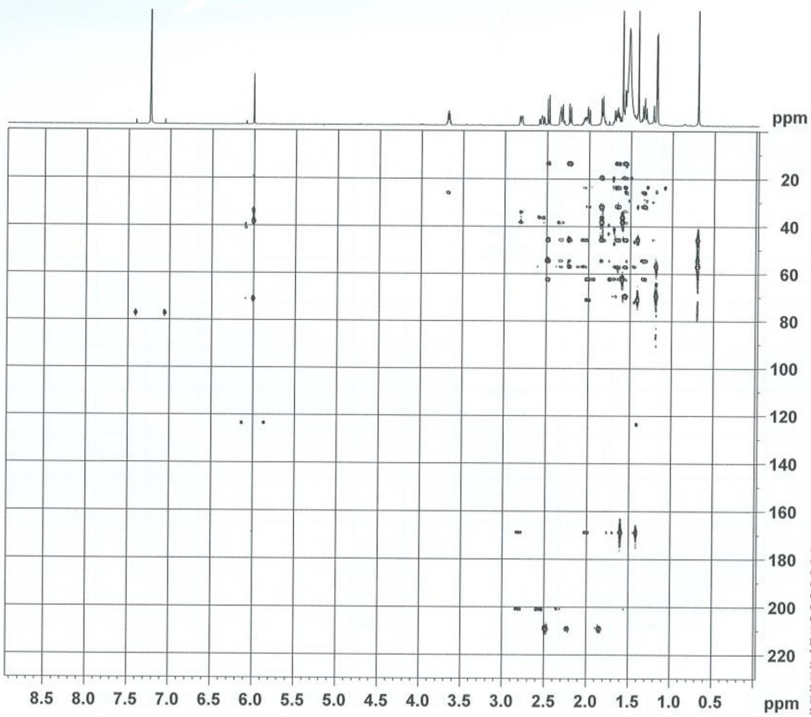

AVANCE AV-600
CRYO PROBE
LAB NO: 108

```
NAME          dec-16-12
EXPNO                 5
PROCNO                1
Date_          20121216
Time              16.42
INSTRUM           spect
PROBHD   5 mm CPTCI 1H-
PULPROG     hmbcgplpndqf
TD                 4096
SOLVENT           CDCl3
NS                   32
DS                    8
SWH            5387.931 Hz
FIDRES         1.315413 Hz
AQ            0.3802516 sec
RG                46341
DW               92.800 usec
DE                 6.50 usec
TE                298.0 K
CNST2       145.0000000
CNST13       13.0000000
D0           0.00000300 sec
D1           1.50000000 sec
D2           0.00344828 sec
D6           0.03846154 sec
D16          0.00015000 sec
IN0          0.00001440 sec

======== CHANNEL f1 ========
NUC1                 1H
P1                 7.20 usec
P2                14.40 usec
PL1                3.30 dB
PL1W         9.16420078 W
SFO1        600.2327010 MHz

======== CHANNEL f2 ========
NUC2                13C
P3                15.40 usec
PL2                1.00 dB
PL2W        83.60149384 W
SFO2        150.9453107 MHz

====== GRADIENT CHANNEL =====
GPNAM1         SINE.100
GPNAM2         SINE.100
GPNAM3         SINE.100
GPZ1              50.00 %
GPZ2              30.00 %
GPZ3              40.10 %
P16             2000.00 usec
ND0                   2
TD                  256
SFO1           150.9453 MHz
FIDRES       135.614929 Hz
SW              230.000 ppm
FnMODE               QF
SI                 1024
```

COMPOUND 6
NOESY

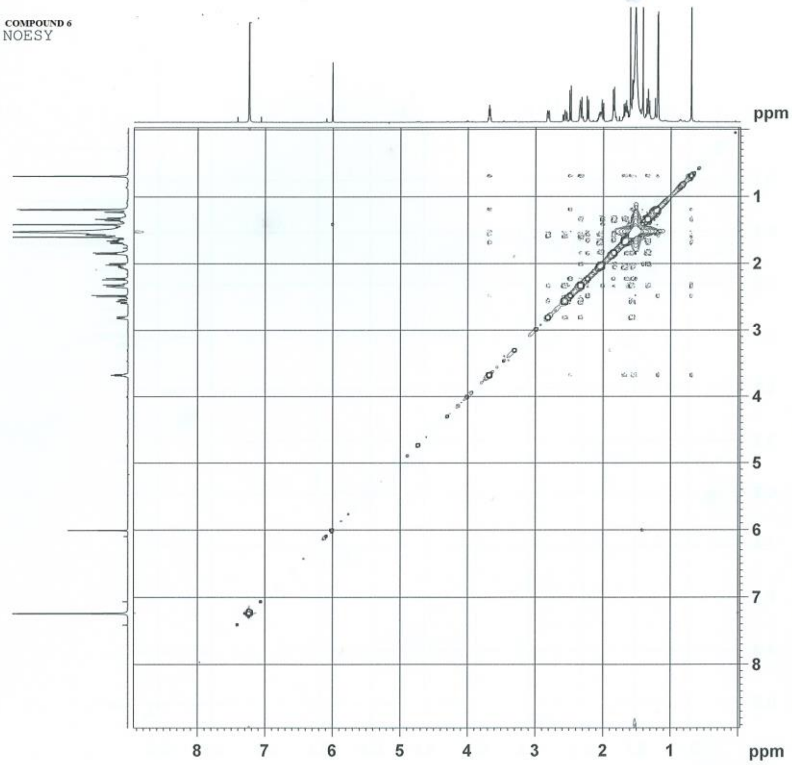

AVANCE AV-600
CRYO PROBE
LAB NO: 108

```
NAME          dec-16-12
EXPNO                 3
PROCNO                1
Date_          20121216
Time              11.37
INSTRUM           spect
PROBHD   5 mm CPTCI 1H-
PULPROG       noesygpph
TD                 1024
SOLVENT           CDCl3
NS                    8
DS                    4
SWH            5387.931 Hz
FIDRES         5.261652 Hz
AQ            0.0951700 sec
RG                 71.8
DW               92.800 usec
DE                 6.50 usec
TE                298.0 K
D0           0.00008363 sec
D1           1.50000000 sec
D8           0.80000001 sec
D16          0.00015000 sec
IN0          0.00018560 sec

======== CHANNEL f1 ========
NUC1                 1H
P1                 7.20 usec
P2                14.40 usec
PL1                3.30 dB
PL1W         9.16420078 W
SFO1        600.2327010 MHz

====== GRADIENT CHANNEL =====
GPNAM1         SINE.100
GPNAM2         SINE.100
GPZ1              40.00 %
GPZ2             -40.00 %
P16             2000.00 usec
ND0                   1
TD                  256
SFO1           600.2327 MHz
FIDRES        21.046606 Hz
SW                8.976 ppm
FnMODE      States-TPPI
SI                 1024
SF          600.2300251 MHz
WDW               QSINE
SSB                   2
LB                 0.00 Hz
GB                    0
PC                 1.40
SI                 1024
MC2         States-TPPI
SF          600.2300251 MHz
WDW               QSINE
SSB                   2
LB                 0.00 Hz
GB                    0
```
