# Supplementary material for: New Anti-Inflammatory Metabolites by Microbial Transformation of Medrysone
Source: PLoS One. 2016 Apr 22;11(4):e0153951. doi: 10.1371/journal.pone.0153951 (PMC4841542; doi:10.1371/journal.pone.0153951)
Supplement: S7 File — (PDF) [file pone.0153951.s007.pdf]

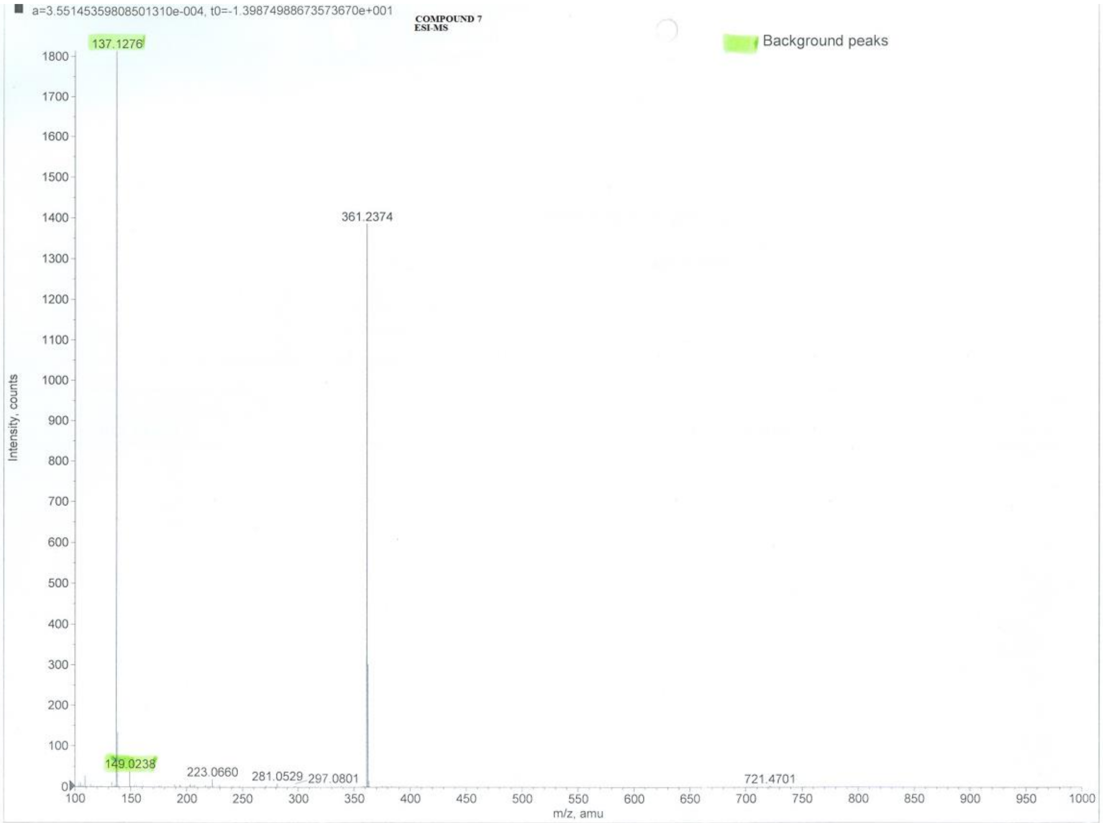

COMPOUND 7
HRESI-MS

Target m/z: +361.2374 amu
Tolerance: +10.0000 ppm
Result type: Elemental
Max num of results: 100
Min DBE: -0.5000 Max DBE: +50.0000
Electron state: OddAndEven
Num of charges: 0
Add water: N/A
Add proton: N/A
File Name: MEDCB-4 25-2-13.wiff

	Elements	Min Number	Max Number
1	C	0	30
2	H	0	50
3	O	0	4

	Formula	Calculated m/z (amu)	mDa Error	PPM Error	DBE
1	C22 H33 O4	361.2378	-0.4848	-1.3422	6.5

COMPOUND 7
H-NMR

AVANCE AV - III
300 MHz, LAB # 116

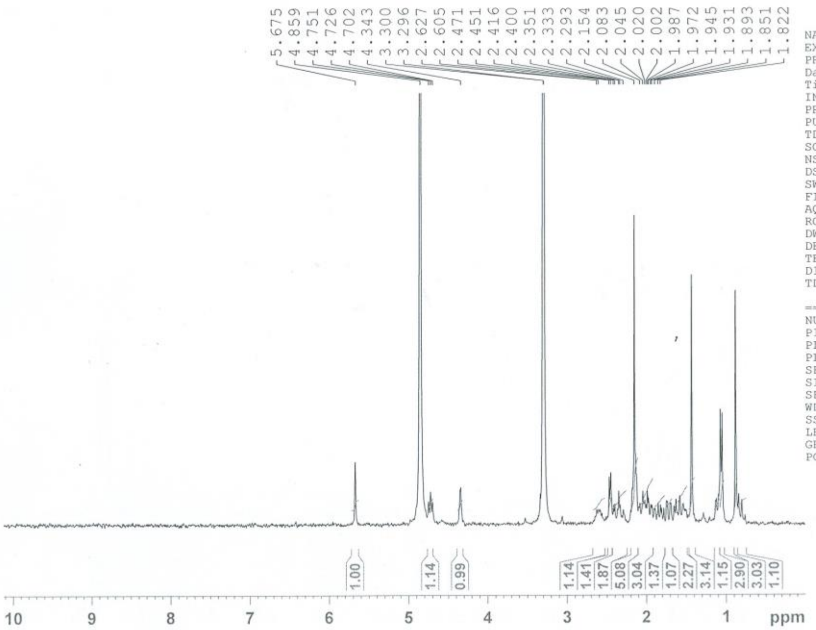

NAME	Nov08
EXPNO	1
PROCNO	1
Date_	20121108
Time	10.49
INSTRUM	Spect
PROBHD	5 mm BBO BB-1H
PULPROG	zg30
TD	32768
SOLVENT	MeOD
NS	32
DS	0
SWH	6188.119 Hz
FIDRES	0.188846 Hz
AQ	2.6477044 sec
RG	203
DW	80.800 usec
DE	6.50 usec
TE	300.0 K
D1	1.50000000 sec
TD0	1

======== CHANNEL f1 ========

NUC1	1H
P1	12.50 usec
PL1	0.00 dB
PL1W	13.16228485 W
SFO1	300.1321009 MHz
SI	16384
SF	300.1300072 MHz
WDW	EM
SSB	0
LB	1.00 Hz
GB	0
PC	1.00

H.E.J. Research Institute of Chemistry.

COMPOUND 7
BB

AVANCE AV-600
CRYO PROBE
LAB NO: 108

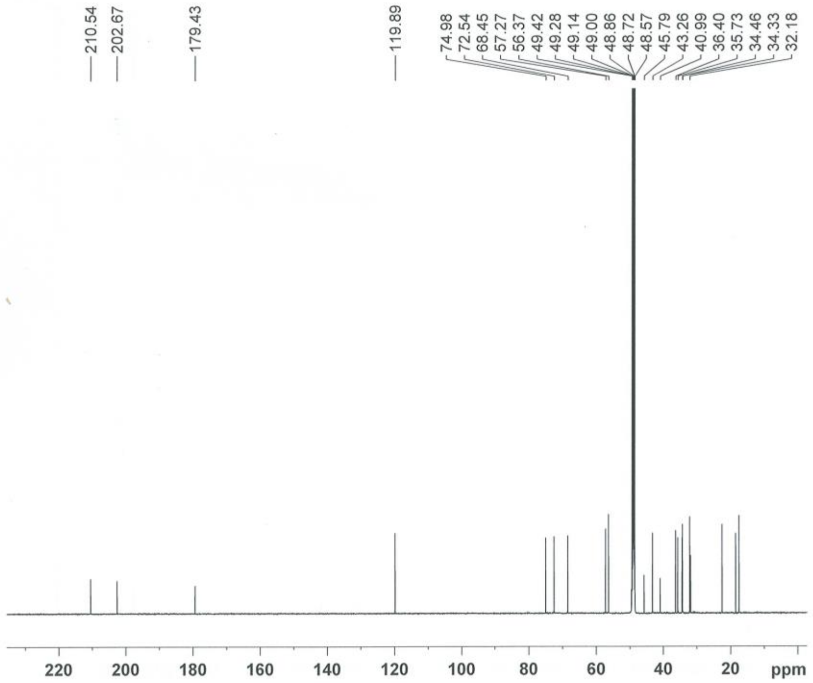

NAME	nov-15-12
EXPNO	11
PROCNO	1
Date_	20121116
Time	3.14
INSTRUM	spect
PROBHD	5 mm CPTCI 1H-
PULPROG	zgpg
TD	65536
SOLVENT	MeOD
NS	8192
DS	2
SWH	35971.223 Hz
FIDRES	0.548877 Hz
AQ	0.9110143 sec
RG	32768
DW	13.900 usec
DE	6.50 usec
TE	298.0 K
D1	1.50000000 sec
D11	0.03000000 sec
TD0	8

======== CHANNEL f1 ========

NUC1	13C
P1	15.40 usec
PL1	1.00 dB
PL1W	83.60149384 W
SFO1	150.9453107 MHz

======== CHANNEL f2 ========

CPDPRG2	waltz16
NUC2	1H
PCPD2	65.00 usec
PL2	3.30 dB
PL12	22.06 dB
PL13	27.00 dB
PL2W	9.16420078 W
PL12W	0.12192553 W
PL13W	0.03909260 W
SFO2	600.2336014 MHz
SI	32768
SF	150.9277406 MHz
WDW	EM
SSB	0
LB	1.00 Hz
GB	0
PC	1.10

COMPOUND 6
DEPT-135

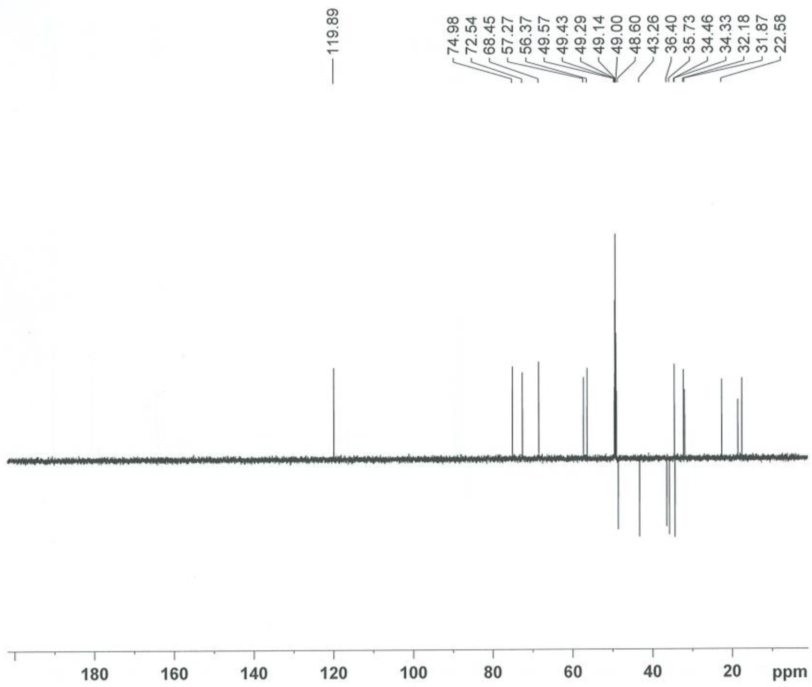

AVANCE AV-600
CRYO PROBE
LAB NO: 108

```
NAME          nov-15-12
EXPNO                12
PROCNO                1
Date_          20121116
Time               8.34
INSTRUM           spect
PROBHD   5 mm CPTCI 1H-
PULPROG      deptsp135
TD                65536
SOLVENT            MeOD
NS                  560
DS                    2
SWH           30303.031 Hz
FIDRES         0.462388 Hz
AQ            1.0814105 sec
RG                32768
DW               16.500 usec
DE                 6.50 usec
TE                298.0 K
CNST2       145.0000000
D1           1.50000000 sec
D2           0.00344828 sec
D12          0.00002000 sec
TD0                   6

======== CHANNEL f1 ========
NUC1                13C
P1                16.00 usec
P12             2000.00 usec
PL0              120.00 dB
PL1                2.00 dB
PL0W         0.00000000 W
PL1W        66.40702820 W
SFO1        150.9430468 MHz
SP2                1.99 dB
SPNAM2      Crp60comp.4
SPOAL2            0.500
SPOFFS2            0.00 Hz

======== CHANNEL f2 ========
CPDPRG2         waltz16
NUC2                 1H
P3                 7.50 usec
P4                15.00 usec
PCPD2             65.00 usec
PL2                3.30 dB
PL12              22.06 dB
PL2W         9.16420078 W
PL12W        0.12192553 W
SFO2        600.2324009 MHz
SI                32768
SF          150.9277406 MHz
WDW                  EM
SSB                   0
LB                 1.00 Hz
GB                    0
PC                 1.00
```

COMPOUND 7
DEPT-90

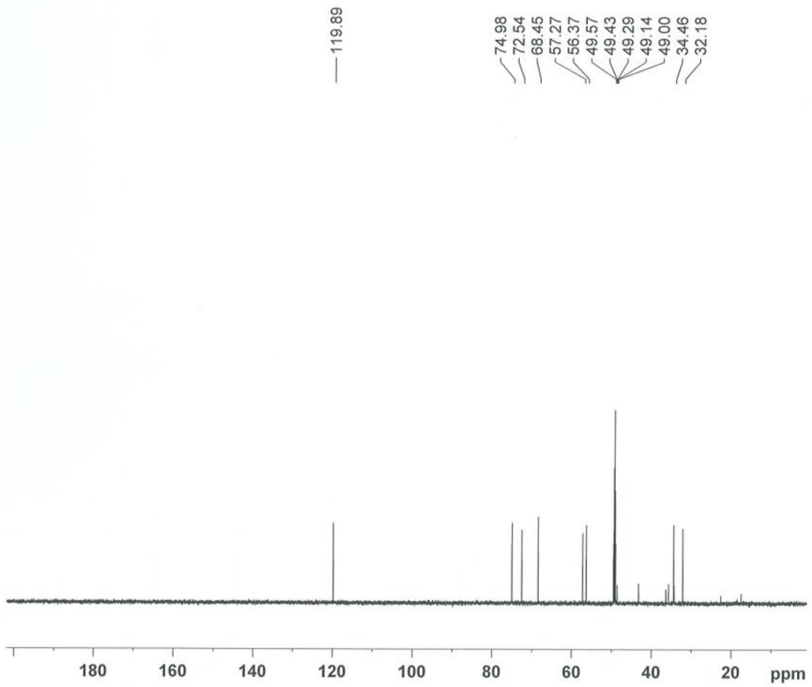

AVANCE AV-600
CRYO PROBE
LAB NO: 108

```
NAME          nov-15-12
EXPNO                13
PROCNO                1
Date_          20121116
Time               9.02
INSTRUM           spect
PROBHD   5 mm CPTCI 1H-
PULPROG       deptsp90
TD                65536
SOLVENT            MeOD
NS                  621
DS                    2
SWH           30303.031 Hz
FIDRES         0.462388 Hz
AQ            1.0814105 sec
RG                32768
DW               16.500 usec
DE                 6.50 usec
TE                298.0 K
CNST2       145.0000000
D1           1.50000000 sec
D2           0.00344828 sec
D12          0.00002000 sec
TD0                   4

======== CHANNEL f1 ========
NUC1                13C
P1                16.00 usec
P12             2000.00 usec
PL0              120.00 dB
PL1                2.00 dB
PL0W         0.00000000 W
PL1W        66.40702820 W
SFO1        150.9430468 MHz
SP2                1.99 dB
SPNAM2      Crp60comp.4
SPOAL2            0.500
SPOFFS2            0.00 Hz

======== CHANNEL f2 ========
CPDPRG2         waltz16
NUC2                 1H
P3                 7.50 usec
P4                15.00 usec
PCPD2             65.00 usec
PL2                3.30 dB
PL12              22.06 dB
PL2W         9.16420078 W
PL12W        0.12192553 W
SFO2        600.2324009 MHz
SI                32768
SF          150.9277406 MHz
WDW                  EM
SSB                   0
LB                 1.00 Hz
GB                    0
PC                 1.40
```

COMPOUND 7

HSQC

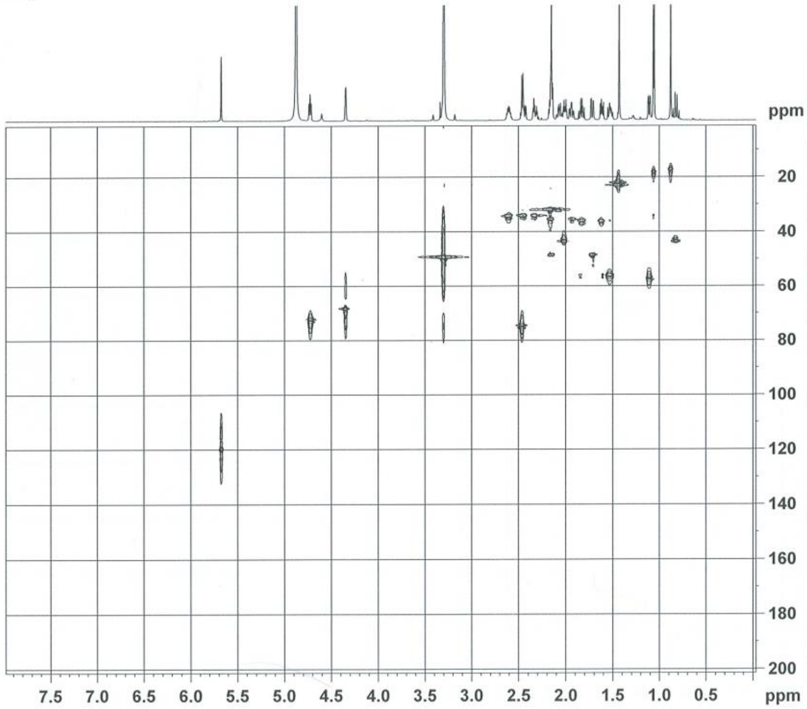

AVANCE AV-600
CRYO PROBE
LAB NO: 108

NAME	nov-15-12
EXPNO	9
PROCNO	1
Date_	20121115
Time	18.19
INSTRUM	spect
PROBHD	5 mm CPTCI 1H-
PULPROG	hsqcetgpsi
TD	1024
SOLVENT	MeOD
NS	32
DS	8
SWH	4807.692 Hz
FIDRES	4.695012 Hz
AQ	0.1066500 sec
RG	26008
DW	104.000 usec
DE	6.50 usec
TE	298.0 K
CNST2	145.0000000
D0	0.00000300 sec
D1	1.50000000 sec
D4	0.00172414 sec
D11	0.03000000 sec
D13	0.00000400 sec
D16	0.00015000 sec
D24	0.00110000 sec
IN0	0.00001655 sec
ZGOPTNS	
======== CHANNEL f1 ========	
NUC1	1H
P1	7.20 usec
P2	14.40 usec
P28	0.50 usec
PL1	3.30 dB
PL1W	9.16420078 W
SFO1	600.2324009 MHz
======== CHANNEL f2 ========	
CPDPRG2	garp
NUC2	13C
P3	15.40 usec
P4	30.80 usec
PCPD2	61.00 usec
PL2	1.00 dB
PL12	13.00 dB
PL2W	83.60149384 W
PL12W	5.27489758 W
SFO2	150.9430468 MHz
====== GRADIENT CHANNEL =====	
GPNAM1	SINE.100
GPNAM2	SINE.100
GPZ1	80.00 %
GPZ2	20.10 %
P16	2000.00 usec
ND0	2

COMPOUND 7

COSY

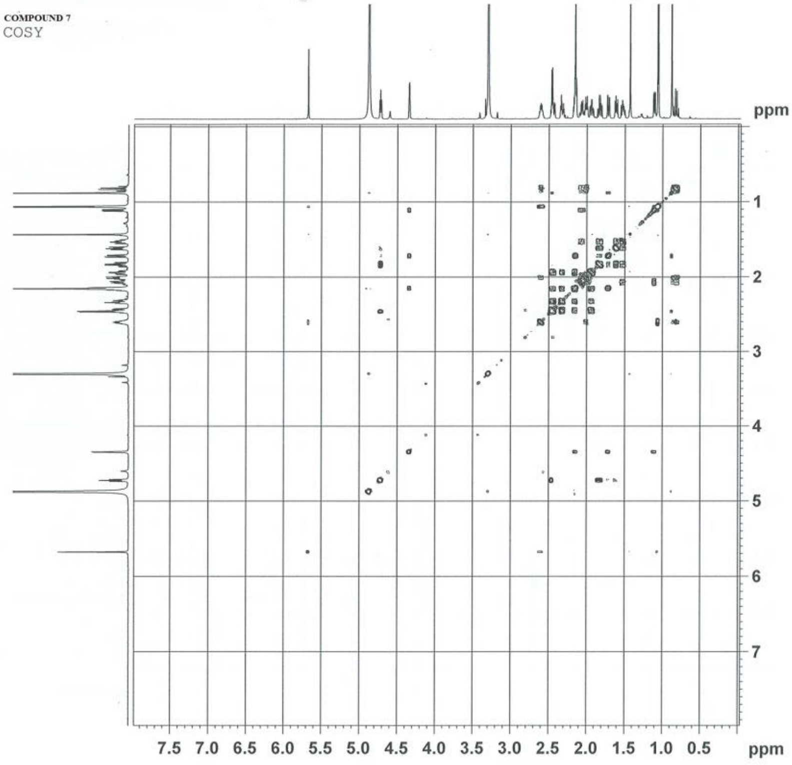

AVANCE AV-600
CRYO PROBE
LAB NO: 108

NAME	nov-15-12
EXPNO	7
PROCNO	1
Date_	20121115
Time	15.55
INSTRUM	spect
PROBHD	5 mm CPTCI 1H-
PULPROG	cosydfqf
TD	2048
SOLVENT	MeOD
NS	8
DS	4
SWH	4807.692 Hz
FIDRES	2.347506 Hz
AQ	0.2131460 sec
RG	32
DW	104.000 usec
DE	6.50 usec
TE	298.1 K
D0	0.00000300 sec
D1	1.50000000 sec
D13	0.00000400 sec
D20	0.00000200 sec
IN0	0.00020800 sec
======== CHANNEL f1 ========	
NUC1	1H
P1	7.20 usec
PL1	3.30 dB
PL1W	9.16420078 W
SFO1	600.2324009 MHz
ND0	1
TD	256
SFO1	600.2324 MHz
FIDRES	18.780046 Hz
SW	8.010 ppm
FnMODE	QF
SI	1024
SF	600.2300156 MHz
WDW	QSINE
SSB	0
LB	0.00 Hz
GB	0
PC	1.40
SI	1024
MC2	QF
SF	600.2300156 MHz
WDW	QSINE
SSB	0
LB	0.00 Hz
GB	0

COMPOUND 7
HMBC

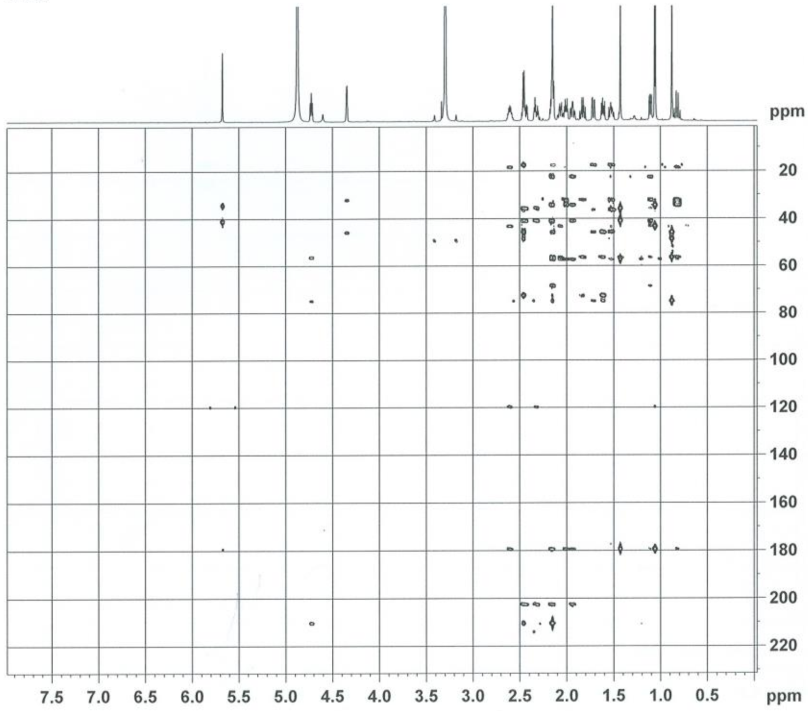

AVANCE AV-600
CRYO PROBE
LAB NO: 108

NAME	nov-15-12
EXPNO	10
PROCNO	1
Date_	20121115
Time	22.02
INSTRUM	spect
PROBHD	5 mm CPTCI 1H-
PULPROG	hmbcgplpndqf
TD	4096
SOLVENT	MeOD
NS	32
DS	8
SWH	4807.692 Hz
FIDRES	1.173753 Hz
AQ	0.4261380 sec
RG	36780.8
DW	104.000 usec
DE	6.50 usec
TE	298.0 K
CNST2	145.0000000
CNST13	13.0000000
D0	0.00000300 sec
D1	1.50000000 sec
D2	0.00344828 sec
D6	0.03846154 sec
D16	0.00015000 sec
IN0	0.00001440 sec
======== CHANNEL f1 ========	
NUC1	1H
P1	7.20 usec
P2	14.40 usec
PL1	3.30 dB
PL1W	9.16420078 W
SFO1	600.2324009 MHz
======== CHANNEL f2 ========	
NUC2	13C
P3	15.40 usec
PL2	1.00 dB
PL2W	83.60149384 W
SFO2	150.9453107 MHz
====== GRADIENT CHANNEL =====	
GPNAM1	SINE.100
GPNAM2	SINE.100
GPNAM3	SINE.100
GPZ1	50.00 %
GPZ2	30.00 %
GPZ3	40.10 %
P16	2000.00 usec
ND0	2
TD	256
SFO1	150.9453 MHz
FIDRES	135.614929 Hz
SW	230.000 ppm
FnMODE	QF
SI	1024

COMPOUND 7
NOESY

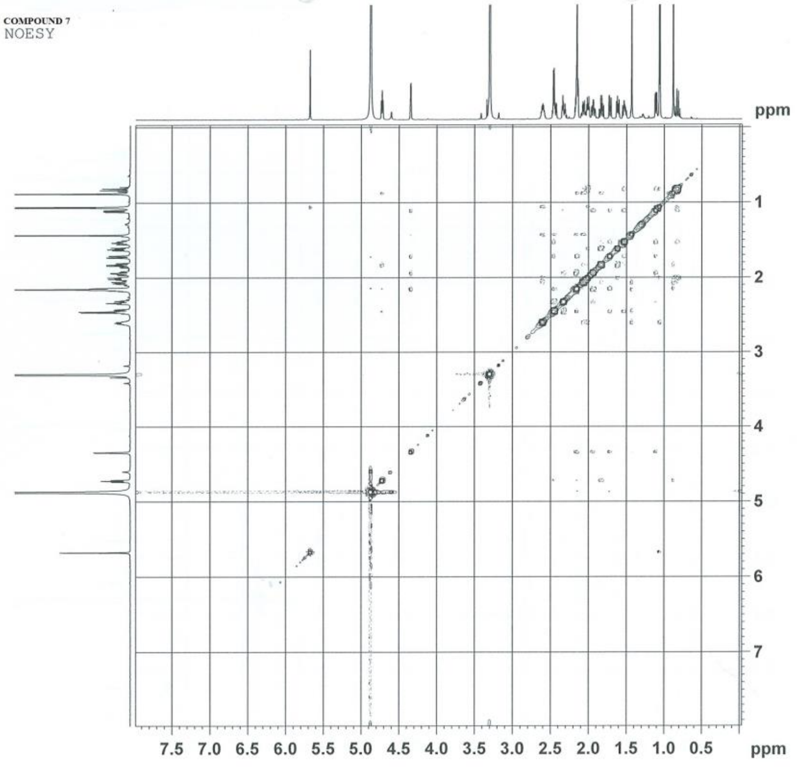

AVANCE AV-600
CRYO PROBE
LAB NO: 108

NAME	nov-15-12
EXPNO	8
PROCNO	1
Date_	20121115
Time	16.55
INSTRUM	spect
PROBHD	5 mm CPTCI 1H-
PULPROG	noesygpph
TD	1024
SOLVENT	MeOD
NS	8
DS	4
SWH	4807.692 Hz
FIDRES	4.695012 Hz
AQ	0.1066500 sec
RG	57
DW	104.000 usec
DE	6.50 usec
TE	298.0 K
D0	0.00009483 sec
D1	1.50000000 sec
D8	0.80000001 sec
D16	0.00015000 sec
IN0	0.00020800 sec
======== CHANNEL f1 ========	
NUC1	1H
P1	7.20 usec
P2	14.40 usec
PL1	3.30 dB
PL1W	9.16420078 W
SFO1	600.2324009 MHz
====== GRADIENT CHANNEL =====	
GPNAM1	SINE.100
GPNAM2	SINE.100
GPZ1	40.00 %
GPZ2	-40.00 %
P16	2000.00 usec
ND0	1
TD	256
SFO1	600.2324 MHz
FIDRES	18.780046 Hz
SW	8.010 ppm
FnMODE	States-TPPI
SI	1024
SF	600.2300156 MHz
WDW	QSINE
SSB	2
LB	0.00 Hz
GB	0
PC	1.40
SI	1024
MC2	States-TPPI
SF	600.2300156 MHz
WDW	QSINE
SSB	2
LB	0.00 Hz
GB	0
