# Supplementary material for: New Anti-Inflammatory Metabolites by Microbial Transformation of Medrysone
Source: PLoS One. 2016 Apr 22;11(4):e0153951. doi: 10.1371/journal.pone.0153951 (PMC4841542; doi:10.1371/journal.pone.0153951)
Supplement: S8 File — (PDF) [file pone.0153951.s008.pdf]

50|52

COMPOUND 8
EI-MS

Instrument : MAT312

SCAN GRAPH. Flagging=M/z.
Scan 13-6:42. Entries=263. 100% Int.=13589.

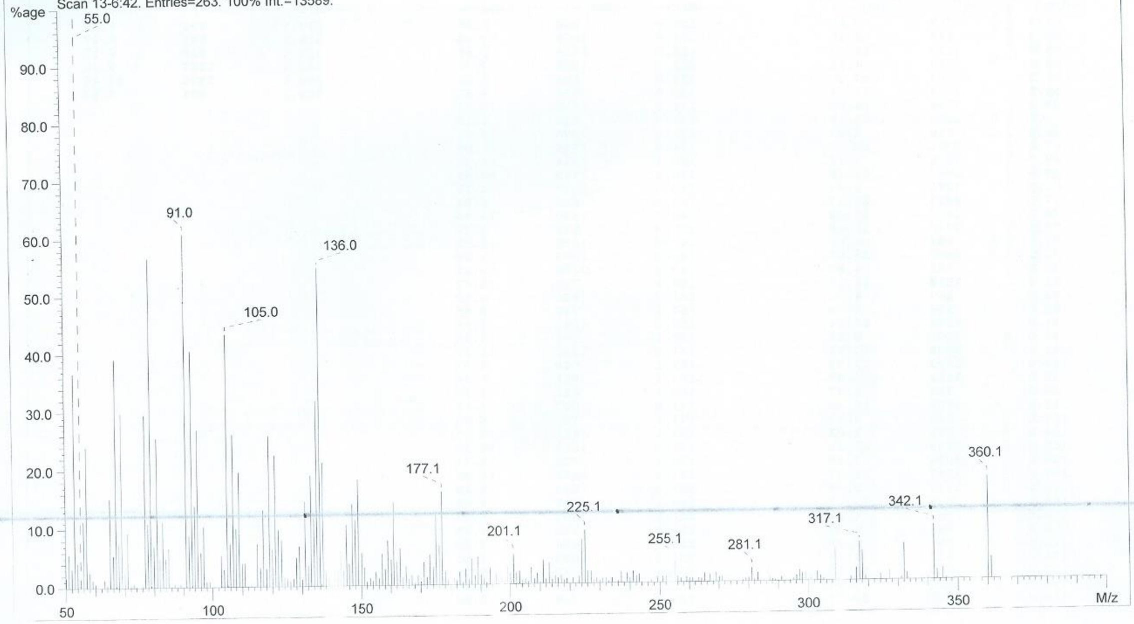

COMPOUND 6
HREI-MS

Mass	Relative Intensity	Theoretical Mass	Delta [mmu]	RDB	Composition
354.9792	1.6	354.9873	-8.1	19.5	$C_{20} H_{3} O_{7}$
360.2295	8.0	360.2295	0.0	7.0	$C_{22} H_{32} O_{4}$
361.2343	1.9	361.2373	-3.1	6.5	$C_{22} H_{33} O_{4}$
366.9792	1.0	366.9873	-8.1	20.5	$C_{21} H_{3} O_{7}$

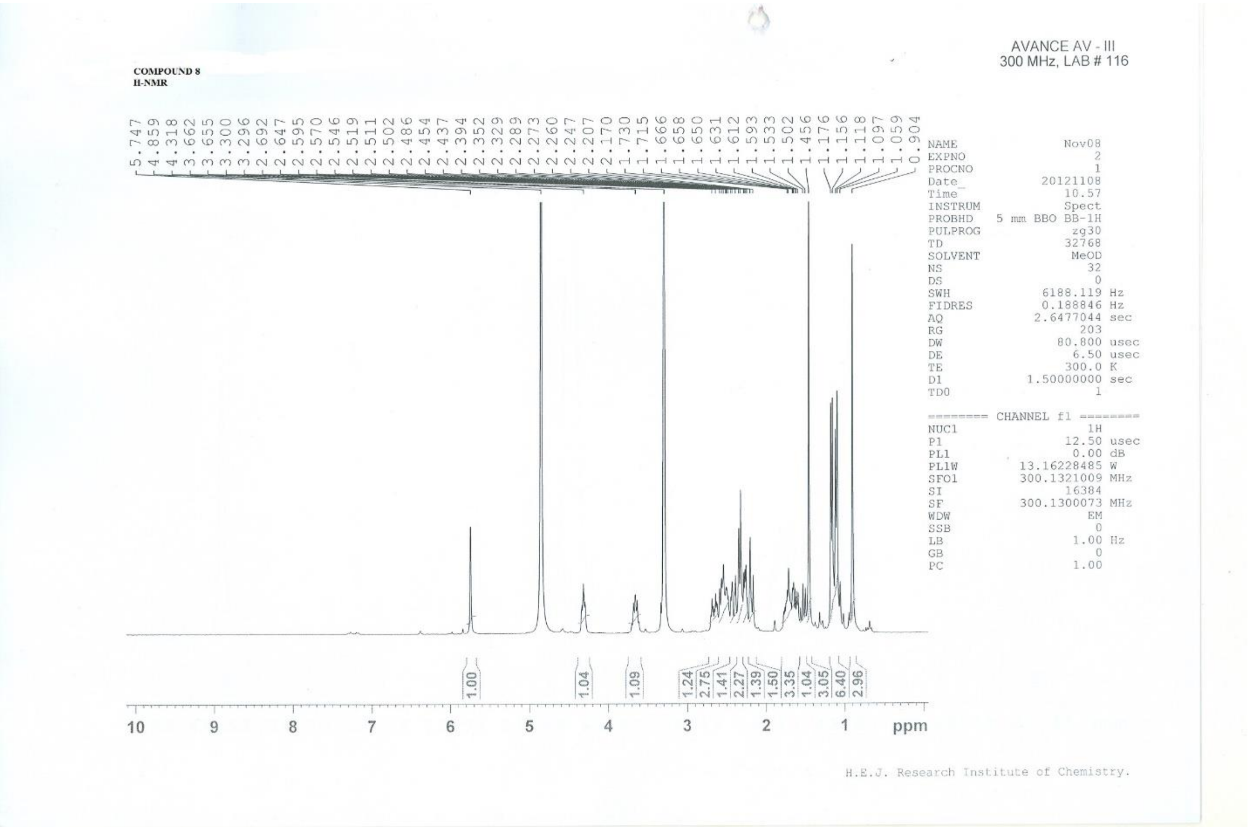

COMPOUND 8
H-NMR
AVANCE AV - III
300 MHz, LAB # 116
5.747 4.859 4.318 3.662 3.655 3.300 3.296 2.692 2.647 2.595 2.570 2.546 2.519 2.511 2.502 2.486 2.454 2.437 2.394 2.352 2.329 2.289 2.273 2.260 2.247 2.207 2.170 1.730 1.715 1.666 1.658 1.650 1.631 1.612 1.593 1.533 1.502 1.456 1.176 1.156 1.118 1.097 1.059 0.904
NAME Nov08
EXPNO 2
PROCNO 1
Date_ 20121108
Time 10.57
INSTRUM Spect
PROBHD 5 mm BBO BB-1H
PULPROG zg30
TD 32768
SOLVENT MeOD
NS 32
DS 0
SWH 6188.119 Hz
FIDRES 0.188846 Hz
AQ 2.6477044 sec
RG 203
DW 80.800 usec
DE 6.50 usec
TE 300.0 K
D1 1.50000000 sec
TD0 1
======== CHANNEL f1 ========
NUC1 1H
P1 12.50 usec
PL1 0.00 dB
PL1W 13.16228485 W
SFO1 300.1321009 MHz
SI 16384
SF 300.1300073 MHz
WDW EM
SSB 0
LB 1.00 Hz
GB 0
PC 1.00
1.00 1.04 1.09 1.24 2.75 1.41 2.27 1.39 1.50 3.35 1.04 3.05 6.40 2.96
10 9 8 7 6 5 4 3 2 1 ppm
H.E.J. Research Institute of Chemistry.

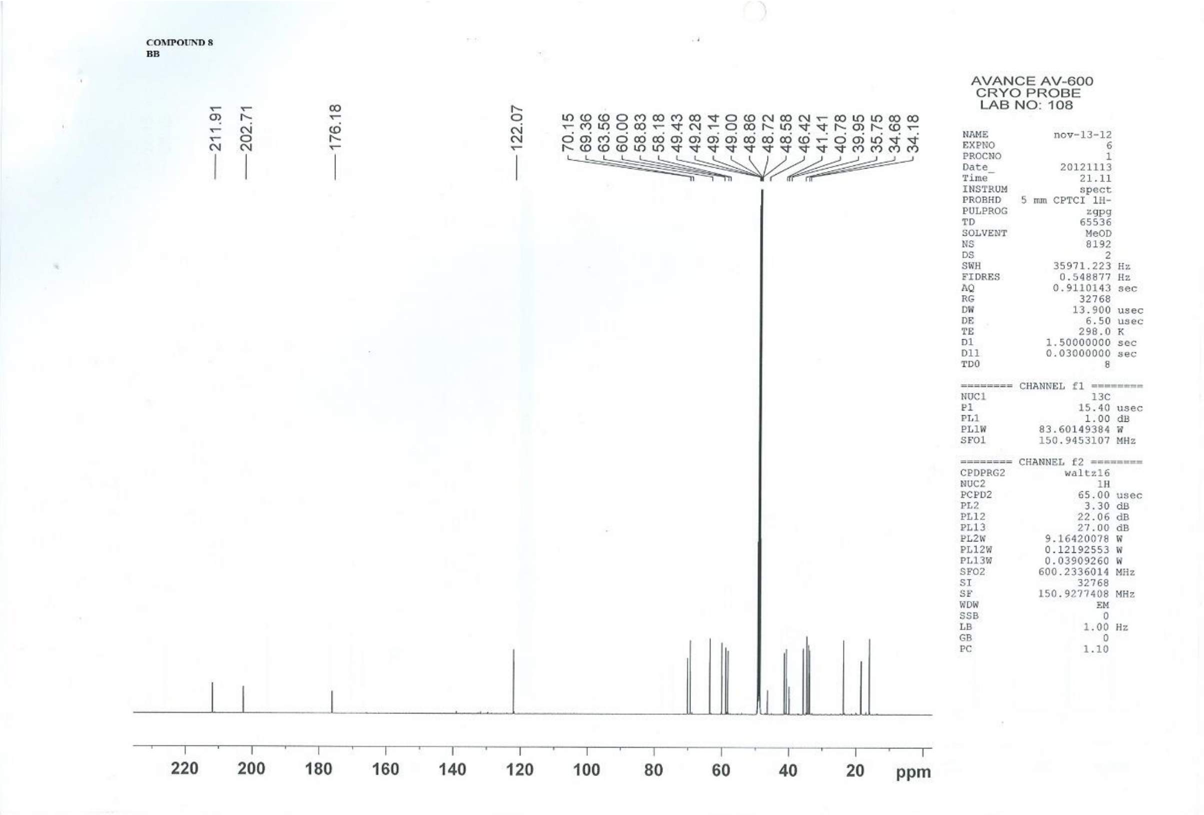

COMPOUND 8
BB
AVANCE AV-600
CRYO PROBE
LAB NO: 108
211.91 202.71 176.18 122.07 70.15 69.36 63.56 60.00 58.83 58.18 49.43 49.28 49.14 49.00 48.86 48.72 48.58 46.42 41.41 40.78 39.95 35.75 34.68 34.18
NAME nov-13-12
EXPNO 6
PROCNO 1
Date_ 20121113
Time 21.11
INSTRUM spect
PROBHD 5 mm CPTCI 1H-
PULPROG zgpg
TD 65536
SOLVENT MeOD
NS 8192
DS 2
SWH 35971.223 Hz
FIDRES 0.548877 Hz
AQ 0.9110143 sec
RG 32768
DW 13.900 usec
DE 6.50 usec
TE 298.0 K
D1 1.50000000 sec
D11 0.03000000 sec
TD0 8
======== CHANNEL f1 ========
NUC1 13C
P1 15.40 usec
PL1 1.00 dB
PL1W 83.60149384 W
SFO1 150.9453107 MHz
======== CHANNEL f2 ========
CPDPRG2 waltz16
NUC2 1H
PCPD2 65.00 usec
PL2 3.30 dB
PL12 22.06 dB
PL13 27.00 dB
PL2W 9.16420078 W
PL12W 0.12192553 W
PL13W 0.03909260 W
SFO2 600.2336014 MHz
SI 32768
SF 150.9277408 MHz
WDW EM
SSB 0
LB 1.00 Hz
GB 0
PC 1.10
220 200 180 160 140 120 100 80 60 40 20 ppm

COMPOUND 8
DEPT-135

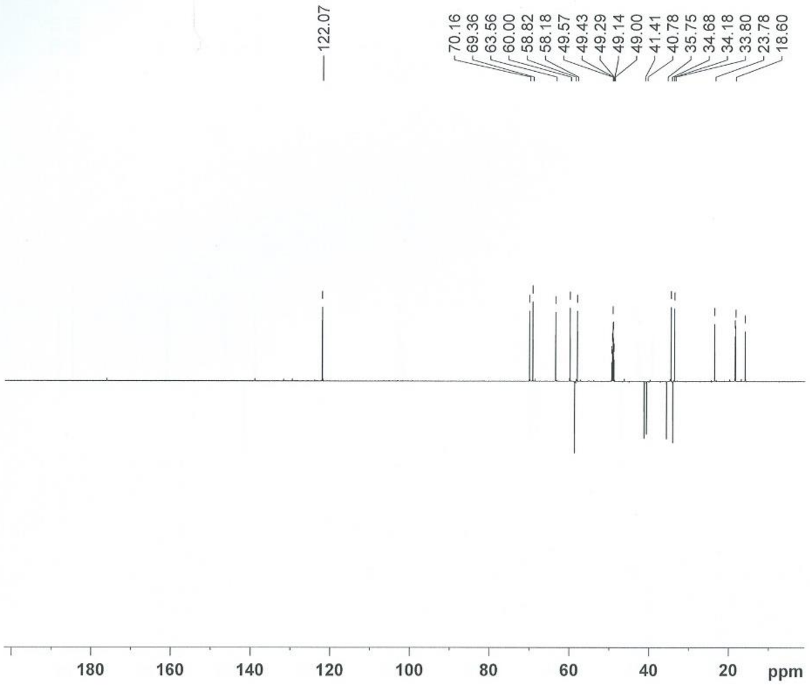

AVANCE AV-600
CRYO PROBE
LAB NO: 108

NAME	nov-13-12
EXPNO	7
PROCNO	1
Date_	20121114
Time	2.52
INSTRUM	spect
PROBHD	5 mm CPTCI 1H-
PULPROG	deptsp135
TD	65536
SOLVENT	MeOD
NS	6144
DS	2
SWH	30303.031 Hz
FIDRES	0.462388 Hz
AQ	1.0814105 sec
RG	32768
DW	16.500 usec
DE	6.50 usec
TE	298.0 K
CNST2	145.0000000
D1	1.50000000 sec
D2	0.00344828 sec
D12	0.00002000 sec
TD0	6
======== CHANNEL f1 ========	
NUC1	13C
P1	16.00 usec
P12	2000.00 usec
PL0	120.00 dB
PL1	2.00 dB
PL0W	0.00000000 W
PL1W	66.40702820 W
SFO1	150.9430468 MHz
SP2	1.99 dB
SPNAM2	Crp60comp.4
SPOAL2	0.500
SPOFFS2	0.00 Hz
======== CHANNEL f2 ========	
CPDPRG2	waltz16
NUC2	1H
P3	7.50 usec
P4	15.00 usec
PCPD2	65.00 usec
PL2	3.30 dB
PL12	22.06 dB
PL2W	9.16420078 W
PL12W	0.12192553 W
SFO2	600.2324009 MHz
SI	32768
SF	150.9277408 MHz
WDW	EM
SSB	0
LB	1.00 Hz
GB	0
PC	1.00

COMPOUND 8
DEPT-90

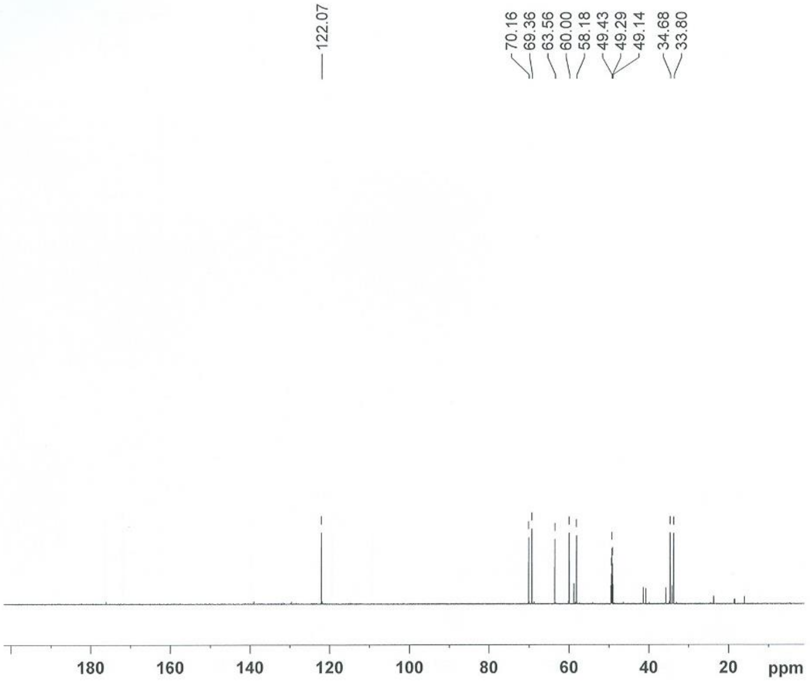

AVANCE AV-600
CRYO PROBE
LAB NO: 108

NAME	nov-13-12
EXPNO	8
PROCNO	1
Date_	20121114
Time	7.22
INSTRUM	spect
PROBHD	5 mm CPTCI 1H-
PULPROG	deptsp90
TD	65536
SOLVENT	MeOD
NS	1166
DS	2
SWH	30303.031 Hz
FIDRES	0.462388 Hz
AQ	1.0814105 sec
RG	32768
DW	16.500 usec
DE	6.50 usec
TE	298.0 K
CNST2	145.0000000
D1	1.50000000 sec
D2	0.00344828 sec
D12	0.00002000 sec
TD0	4
======== CHANNEL f1 ========	
NUC1	13C
P1	16.00 usec
P12	2000.00 usec
PL0	120.00 dB
PL1	2.00 dB
PL0W	0.00000000 W
PL1W	66.40702820 W
SFO1	150.9430468 MHz
SP2	1.99 dB
SPNAM2	Crp60comp.4
SPOAL2	0.500
SPOFFS2	0.00 Hz
======== CHANNEL f2 ========	
CPDPRG2	waltz16
NUC2	1H
P3	7.50 usec
P4	15.00 usec
PCPD2	65.00 usec
PL2	3.30 dB
PL12	22.06 dB
PL2W	9.16420078 W
PL12W	0.12192553 W
SFO2	600.2324009 MHz
SI	32768
SF	150.9277408 MHz
WDW	EM
SSB	0
LB	1.00 Hz
GB	0
PC	1.40

COMPOUND 8
HSQC

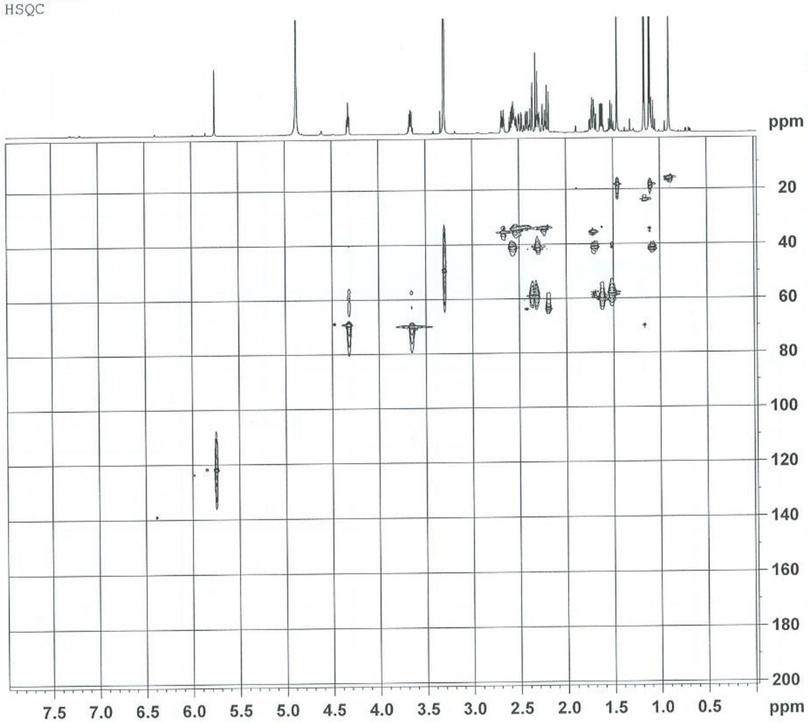

AVANCE AV-600
CRYO PROBE
LAB NO: 108

```
NAME          nov-13-12
EXPNO                 4
PROCNO                1
Date_          20121113
Time              12.14
INSTRUM           spect
PROBHD   5 mm CPTCI 1H-
PULPROG      hsqcetgpsi
TD                 1024
SOLVENT            MeOD
NS                   32
DS                    8
SWH            4807.692 Hz
FIDRES         4.695012 Hz
AQ            0.1066500 sec
RG                26008
DW              104.000 usec
DE                 6.50 usec
TE                298.0 K
CNST2       145.0000000
D0           0.00000300 sec
D1           1.50000000 sec
D4           0.00172414 sec
D11          0.03000000 sec
D13          0.00000400 sec
D16          0.00015000 sec
D24          0.00110000 sec
IN0          0.00001655 sec
ZGOPTNS

======== CHANNEL f1 ========
NUC1                 1H
P1                 7.20 usec
P2                14.40 usec
P28                0.50 usec
PL1                3.30 dB
PL1W         9.16420078 W
SFO1        600.2324009 MHz

======== CHANNEL f2 ========
CPDPRG2            garp
NUC2                13C
P3                15.40 usec
P4                30.80 usec
PCPD2             61.00 usec
PL2                1.00 dB
PL12              13.00 dB
PL2W        83.60149384 W
PL12W        5.27489758 W
SFO2        150.9430468 MHz

====== GRADIENT CHANNEL =====
GPNAM1         SINE.100
GPNAM2         SINE.100
GPZ1              80.00 %
GPZ2              20.10 %
P16             2000.00 usec
ND0                   2
```

COMPOUND 8
COSY

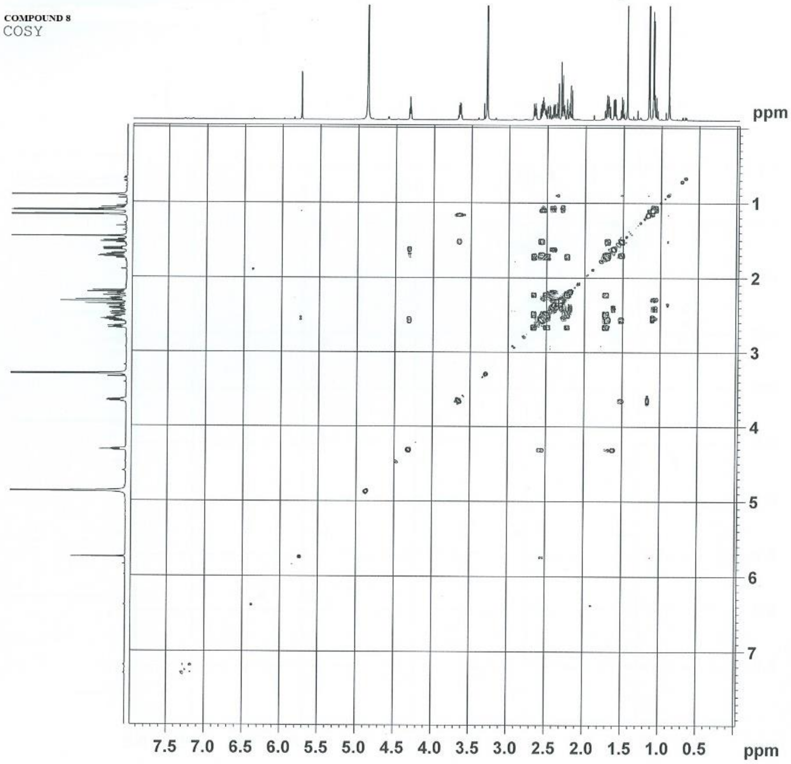

AVANCE AV-600
CRYO PROBE
LAB NO: 108

```
NAME          nov-13-12
EXPNO                 2
PROCNO                1
Date_          20121113
Time               9.51
INSTRUM           spect
PROBHD   5 mm CPTCI 1H-
PULPROG        cosydfqf
TD                 2048
SOLVENT            MeOD
NS                    8
DS                    4
SWH            4807.692 Hz
FIDRES         2.347506 Hz
AQ            0.2131460 sec
RG                   32
DW              104.000 usec
DE                 6.50 usec
TE                298.0 K
D0           0.00000300 sec
D1           1.50000000 sec
D13          0.00000400 sec
D20          0.00000200 sec
IN0          0.00020800 sec

======== CHANNEL f1 ========
NUC1                 1H
P1                 7.20 usec
PL1                3.30 dB
PL1W         9.16420078 W
SFO1        600.2324009 MHz
ND0                   1
TD                  256
SFO1           600.2324 MHz
FIDRES        18.780046 Hz
SW                8.010 ppm
FnMODE               QF
SI                 1024
SF          600.2300156 MHz
WDW               QSINE
SSB                   0
LB                 0.00 Hz
GB                    0
PC                 1.40
SI                 1024
MC2                  QF
SF          600.2300156 MHz
WDW               QSINE
SSB                   0
LB                 0.00 Hz
GB                    0
```

COMPOUND 8

HMBC

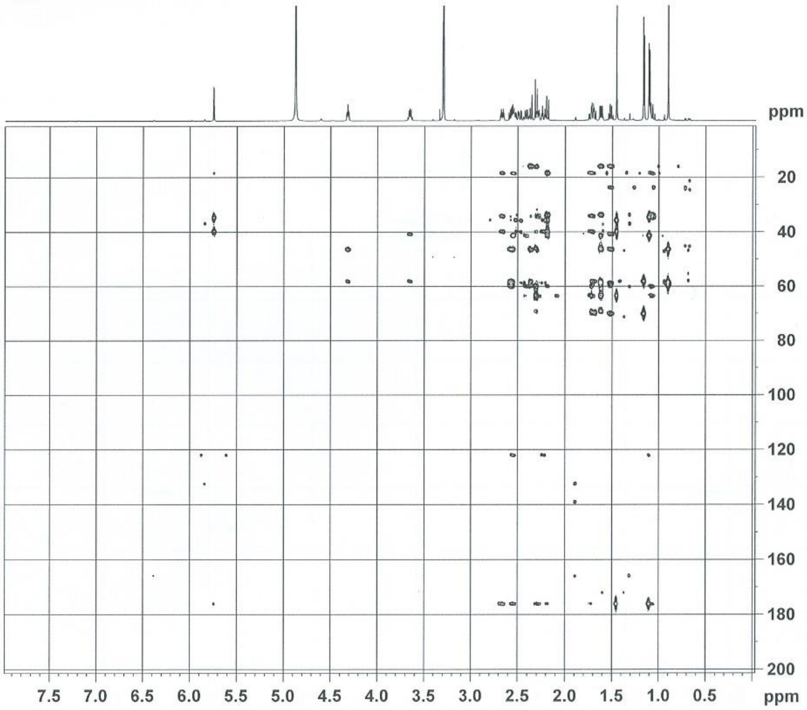

AVANCE AV-600
CRYO PROBE
LAB NO: 108

```
NAME           nov-13-12
EXPNO                  5
PROCNO                 1
Date_           20121113
Time               15.58
INSTRUM            spect
PROBHD   5 mm CPTCI 1H-
PULPROG   hmbcgplpndqf
TD                  4096
SOLVENT             MeOD
NS                    32
DS                     8
SWH             4807.692 Hz
FIDRES          1.173753 Hz
AQ             0.4261380 sec
RG               36780.8
DW               104.000 usec
DE                  6.50 usec
TE                 298.0 K
CNST2        145.0000000
CNST13        13.0000000
D0            0.00000300 sec
D1            1.50000000 sec
D2            0.00344828 sec
D6            0.03846154 sec
D16           0.00015000 sec
IN0           0.00001440 sec

======== CHANNEL f1 ========
NUC1                  1H
P1                  7.20 usec
P2                 14.40 usec
PL1                 3.30 dB
PL1W          9.16420078 W
SFO1        600.2324009 MHz

======== CHANNEL f2 ========
NUC2                 13C
P3                 15.40 usec
PL2                 1.00 dB
PL2W         83.60149384 W
SFO2        150.9453107 MHz

===== GRADIENT CHANNEL =====
GPNAM1         SINE.100
GPNAM2         SINE.100
GPNAM3         SINE.100
GPZ1             50.00 %
GPZ2             30.00 %
GPZ3             40.10 %
P16            2000.00 usec
ND0                   2
TD                  256
SFO1           150.9453 MHz
FIDRES       135.614929 Hz
SW              230.000 ppm
FnMODE               QF
SI                 1024
```

COMPOUND 8

NOESY

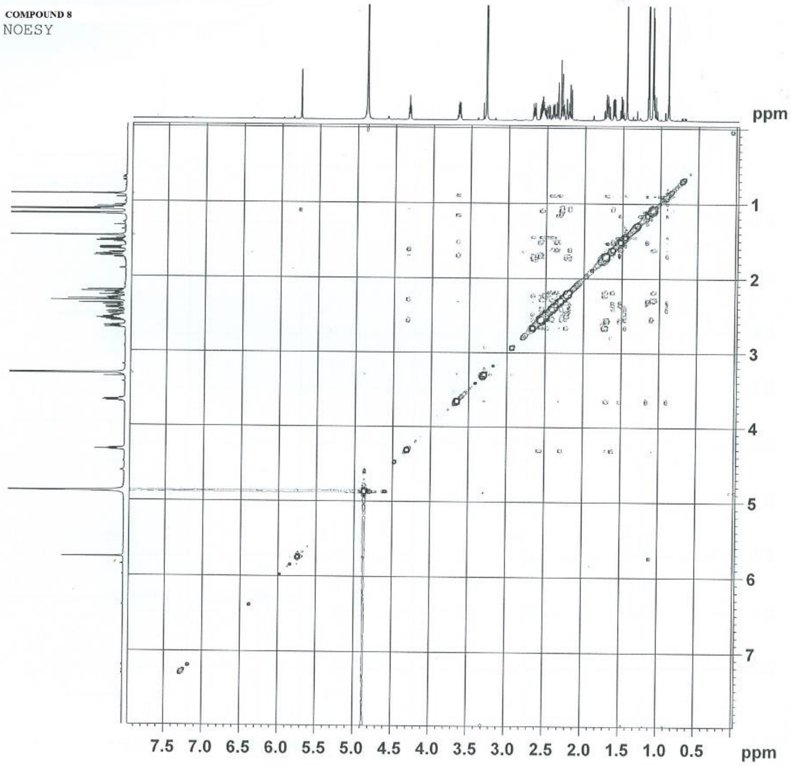

AVANCE AV-600
CRYO PROBE
LAB NO: 108

```
NAME           nov-13-12
EXPNO                  3
PROCNO                 1
Date_           20121113
Time               10.51
INSTRUM            spect
PROBHD   5 mm CPTCI 1H-
PULPROG      noesygpph
TD                  1024
SOLVENT             MeOD
NS                     8
DS                     4
SWH             4807.692 Hz
FIDRES          4.695012 Hz
AQ             0.1066500 sec
RG                    64
DW               104.000 usec
DE                  6.50 usec
TE                 298.0 K
D0            0.00009483 sec
D1            1.50000000 sec
D8            0.80000001 sec
D16           0.00015000 sec
IN0           0.00020800 sec

======== CHANNEL f1 ========
NUC1                  1H
P1                  7.20 usec
P2                 14.40 usec
PL1                 3.30 dB
PL1W          9.16420078 W
SFO1        600.2324009 MHz

===== GRADIENT CHANNEL =====
GPNAM1         SINE.100
GPNAM2         SINE.100
GPZ1             40.00 %
GPZ2            -40.00 %
P16            2000.00 usec
ND0                   1
TD                  256
SFO1           600.2324 MHz
FIDRES        18.780046 Hz
SW                8.010 ppm
FnMODE      States-TPPI
SI                 1024
SF          600.2300156 MHz
WDW               QSINE
SSB                   2
LB                 0.00 Hz
GB                    0
PC                 1.40
SI                 1024
MC2         States-TPPI
SF          600.2300156 MHz
WDW               QSINE
SSB                   2
LB                 0.00 Hz
GB                    0
```
